# Illumina error correction near highly repetitive DNA regions improves de novo genome assembly

**DOI:** 10.1186/s12859-019-2906-2

**Published:** 2019-06-03

**Authors:** Mahdi Heydari, Giles Miclotte, Yves Van de Peer, Jan Fostier

**Affiliations:** 1 Department of Information Technology, Ghent University-imec, IDLab, Ghent, B-9052 Belgium; 2 Bioinformatics Institute Ghent, Ghent, B-9052 Belgium; 3 Center for Plant Systems Biology, VIB, Ghent, B-9052 Belgium; 4 Department of Plant Biotechnology and Bioinformatics, Ghent University, Ghent, B-9052 Belgium; 5 Department of Genetics, Genome Research Institute, University of Pretoria, Pretoria, South Africa

**Keywords:** Illumina sequencing data, De novo genome assembly, Error correction, De Bruijn Graph, Community detection

## Abstract

**Background:**

Several standalone error correction tools have been proposed to correct sequencing errors in Illumina data in order to facilitate de novo genome assembly. However, in a recent survey, we showed that state-of-the-art assemblers often did not benefit from this pre-correction step. We found that many error correction tools introduce new errors in reads that overlap highly repetitive DNA regions such as low-complexity patterns or short homopolymers, ultimately leading to a more fragmented assembly.

**Results:**

We propose BrownieCorrector, an error correction tool for Illumina sequencing data that focuses on the correction of only those reads that overlap short DNA patterns that are highly repetitive in the genome. BrownieCorrector extracts all reads that contain such a pattern and clusters them into different groups using a community detection algorithm that takes into account both the sequence similarity between overlapping reads and their respective paired-end reads. Each cluster holds reads that originate from the same genomic region and hence each cluster can be corrected individually, thus providing a consistent correction for all reads within that cluster.

**Conclusions:**

BrownieCorrector is benchmarked using six real Illumina datasets for different eukaryotic genomes. The prior use of BrownieCorrector improves assembly results over the use of uncorrected reads in all cases. In comparison with other error correction tools, BrownieCorrector leads to the best assembly results in most cases even though less than 2% of the reads within a dataset are corrected. Additionally, we investigate the impact of error correction on hybrid assembly where the corrected Illumina reads are supplemented with PacBio data. Our results confirm that BrownieCorrector improves the quality of hybrid genome assembly as well. BrownieCorrector is written in standard C++11 and released under GPL license. BrownieCorrector relies on multithreading to take advantage of multi-core/multi-CPU systems. The source code is available at https://github.com/biointec/browniecorrector.

**Electronic supplementary material:**

The online version of this article (10.1186/s12859-019-2906-2) contains supplementary material, which is available to authorized users.

## Introduction

Illumina platforms generate accurate sequencing data with high throughput at a low financial cost. It is estimated that more than 90% of sequencing data worldwide are generated by Illumina platforms. These data are characterized by a relatively short read length (100-300 bp) and low error rate (1-2% errors). Despite this relatively high accuracy, Illumina data suffers from different kinds of biases, most notably a higher number of sequencing errors towards the end of the reads. The most common errors are substitutions whereas insertions and deletions are less common and particularly occur in homopolymers [1]. Phenomena like crosstalk, phasing, fading or T accumulation can be major sources of errors in Illumina sequencing machines [2].

Due to its cost-efficiency and high accuracy, Illumina data is frequently used for de novo genome assembly, sometimes complemented by data generated through other platforms (e.g. Pacific Biosciences, Oxford Nanopore). Short-read assemblers typically rely on the de Bruijn graph (DBG) data structure in which overlap between reads is established in a computationally efficient manner through the identification of shared *k*-mers. Yet, the presence of sequencing errors challenges de novo genome assembly tools: sequencing errors result in erroneous nodes and arcs in the DBG, often classified as ‘tips’ (dead ends), ‘bubbles’ (parallel paths) and ‘chimeric connections’ (spurious connections) [3]. As a single sequencing error leads to up to *k* erroneous *k*-mers in the DBG, true nodes in the DBG are vastly outnumbered by erroneous nodes. These artifacts highly complicate the task of identifying the path in the graph that represents the original genomic sequence.

Trimming tools are sometimes used as a primary solution to exclude parts of the input data with a lower quality score. However, this further reduces the read length and aggravates the coverage bias. Additionally, indels are often not associated with a low quality score [4], rendering it difficult to remove them by trimming reads. Recently, a number of standalone error correction (EC) tools have been proposed which aim to identify and correct errors in sequencing data: ACE [5], BayesHammer [6], BFC [7], BLESS [8], BLESS 2 [9], Blue [10], Fiona [11], Karect [12], Lighter [13], Musket [14], Pollux [15], Quake [16], QuorUM [17], RACER [18], RECKONER [19], SGA-EC [20] and Trowel [21]. The key idea is that the prior application of EC tools to raw Illumina data provides a cleaner input dataset to the assemblers and subsequently leads to improved assemblies.

However, in a recent survey [22], we showed that state-of-the-art assemblers such as SPAdes [23] and Discovar [24] did not benefit much from this pre-correction step. In fact, the prior use of EC tools was often found to deteriorate assembly results. Most of the EC tools successfully detect and correct a large fraction of sequencing errors, however, most of these errors are harmless to the assembly process as they are properly handled by the assembly tools as well. Specifically, the vast majority of sequencing errors lead to short spurious dead ends or short parallel paths which are easily identified and removed from the DBG based on graph topology and coverage considerations. On the other hand, in certain genomic contexts, EC tools have difficulties identifying sequencing errors and might even introduce new errors. In turn, this may result in misassemblies or assembly breakpoints, leading to shorter contigs/scaffolds. In [22], we reported that misassemblies and breakpoints often occur in two regions: (i) genomic regions with low read coverage where the EC tools incorrectly transform true *k*-mers into similar *k*-mers with higher coverage and (ii), the direct vicinity of short, highly repetitive patterns such as homopolymers. We found that EC tools often modify reads that overlap such pattern in an inconsistent manner.

We introduce BrownieCorrector, an EC tool for Illumina sequencing data that focuses solely on the correction of (paired-end) reads that overlap highly repetitive patterns. BrownieCorrector performs four steps: (i) selection of a repetitive *k*-mer, (ii) read extraction, (iii) read clustering and (iv) per-cluster read error correction. Initially, it selects a highly repetitive *k*-mer such as a short poly(A/T) pattern and identifies all paired-end reads for which one of the paired reads contains that *k*-mer. Next, using a community detection algorithm, it clusters the read pairs such that each cluster contains read pairs that overlap with the same genomic region. As a similarity score for the clustering algorithm, BrownieCorrector computes the overlap alignment score (a variation of the Needleman-Wunsch alignment score [25]) between different read pairs. The read clustering problem is expressed as a community detection problem in graph theory [26]. The Louvain community detection algorithm [27] is applied to an undirected weighted graph whose nodes represent paired-end reads while an edge between two nodes denotes their similarity score. Edges exist only in case the similarity score exceeds a threshold. Hence, the graph is generally sparse. In order to have a robust clustering, BrownieCorrector repeats the community detection process multiple times with different initialization conditions and identifies stable community cores in the network [28]. These cores contain read pairs that were often clustered together in the different runs of the community detection algorithm. The reads are corrected for each cluster separately. From the (paired-end) reads in a particular cluster, BrownieCorrector first constructs the associated DBG. It then performs typical graph cleaning procedures such as tip clipping and bubble detection to remove erroneous nodes and arcs, taking into account both the graph topology and the *k*-mer frequency (i.e., the number of reads that support each node/arc). Finally, the reads in a cluster are aligned back to the cleaned DBG using BrownieAligner [29]. A similar approach has been already employed for the correction of long reads in LoRDEC [30] and Jabba [31] which has been shown to work effectively even for those errors prone sequencing technologies. This way, sequencing errors are identified and corrected in a consistent manner for all reads within a cluster. This procedure is repeated for all clusters individually.

Correcting smaller groups of reads in each cluster independently from other clusters has a number of advantages over tools that try and correct the entire dataset: first, a small *k*-mer size (for example *k*=15) can be used to construct the DBG of each cluster. This allows to establish overlap between individual reads with increased sensitivity without suffering from chimeric connections. This is particularly relevant for low-coverage regions. Second, as each cluster is expected to contain reads from a single genomic region, reads are corrected in a consistent manner.

Note that only a small fraction of pairs are corrected using BrownieCorrector. Read pairs that do not contain highly repetitive *k*-mer are not modified. The rationale is that state-of-the-art genome de novo assembly tools handle such reads very well. To the best of our knowledge, BrownieCorrector is the first EC tool that uses the paired-read read information in the error correction process, whereas other error correction tools correct reads or even *k*-mers individually.

## Methods

### Error correction tools

The performance of BrownieCorrector is compared with the state-of-the-art EC tools which are all published in 2015 or later: ACE, BLESS 2, BFC, Karect and RECKONER. All tools were run on a machine with four Intel(R) Xeon(R) E5-2698 v3 @ 2.30 GHz CPUs (64 cores in total) and 256 GB of memory. All tools support multi-threading and were run with 64 threads. BLESS 2 failed to finish with 64 cores in some data sets, hence we used 32 cores to get the corrected reads. For all results the default or recommended parameters are used. Parameters and settings are provided in Additional file 1: 1. Elapsed (wall clock) time and peak resident memory were measured with the GNU *time* command.

### Evaluation tools

SPAdes is a universal de novo genome assembler which removes erroneous *k*-mers through the identification of bubbles and tips in multisized DBGs. In a recent comprehensive study [22], SPAdes is compared to DISCOVAR [24], IDBA [32] and Velvet [3], and it was shown that SPAdes produces longer and more accurate contigs/scaffolds than other assemblers, both with and without pre-correcting reads. SPAdes works with Illumina single-end, paired-end and mate-pair read data and can effectively be used for hybrid assembly where reads from other platforms such as Ion Torrent, PacBio, Oxford Nanopore are also provided. Therefore, in this study, SPAdes is used to evaluate the impact of error correction on de novo genome assembly results. SPAdes is provided with a standalone EC tool (BayesHammer) that can apply error correction to the input reads prior to the actual assembly process. All assembly results in this work were obtained without the use of BayesHammer by providing the –only-assembler flag to SPAdes in all cases. Note however that the assembly module within SPAdes also applies error correction procedures directly on the de Bruijn graphs. The resulting assemblies were evaluated using QUAST [33]. In order to determine *k*-mer frequencies Jellyfish [34] is used. To align reads to the reference genome BWA [35] is used.

### Data

Tools are evaluated on six real Illumina eukaryotic datasets for which a high-quality reference genome is available: human chromosomes 14 and 21, two different datasets for fruit fly (*Drosophila melanogaster*), one nematode (*Caenorhabditis elegans*) and one plant organism (*Arabidopsis thaliana*) (see Table 1). Genome sizes range from 45.2 Mbp (Homo sapiens chr. 21) to 135 Mbp (*A. thaliana*) while read coverage varies between 29× and 67×. All datasets have fixed read lengths. In addition, two publicly available PacBio datasets for *D. melanogaster* and *A. thaliana* are used to evaluate the impact of EC tools on hybrid assembly (See Additional file 1: 2.1 and 2.2).

**Table 1:** Real datasets used for the evaluation of the error correction tools

Abbr.	Organism	Reference ID	Reference	Platform	Insert size	Insert size	Cov.	Number of	Read length	Ref.	Dataset ID
			size		mean	STD		Reads	mean		
R1	*Homo sapiens* chr. 21	HG19	40 Mbp	Illumina	312	14	33 ×	13 486 136	100 bp	[10, 22]	Ill. Data library
R2	*Homo sapiens* chr. 14	HG14	104 Mbp	Illumina	158	17	35 ×	36 504 800	101 bp	[12]	GAGE
R3	*Caenorhabditis elegans*	WS222	97 Mbp	Illumina	173	16	58 ×	57 721 732	101 bp	[5, 22, 37]	SRR543736
R4	*Drosophila melanogaster*	Release 5	116 Mbp	Illumina	281	92	52 ×	63 014 762	100 bp	[5, 22, 37]	SRR823377
R5	*Drosophila melanogaster*	Release 5	116 Mbp	Illumina	598	39	64 ×	75 938 276	101 bp	[5, 37]	SRR988075
R6	*Arabidopsis thaliana*	TAIR10	116 Mbp	Illumina	477	18	72 ×	93 429 346	90 bp	[38]	SRR1174256
P1	*Drosophila melanogaster*	Release 5	116 Mbp	PacBio	n/a	n/a	10 ×	169 923	7374 bp	[39]	SRR1204466
P2	*Arabidopsis thaliana*	TAIR10	116 Mbp	PacBio	n/a	n/a	13 ×	187 292	8298 bp	[39]	SRR1284707

Note the absence of bacterial datasets. As also observed in [22], error correction often does not have a significant impact on the assembly quality for such small genomes.

### Targeted error correction

**Fig. 1:**
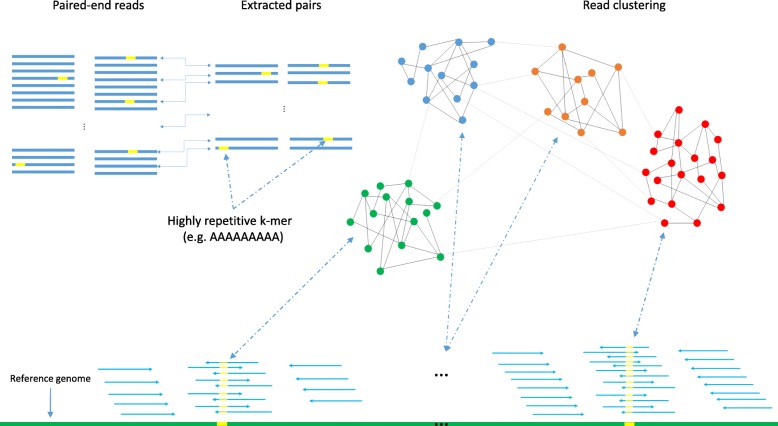
Overview of the first three steps of BrownieCorrector’s pipeline. Read pairs for which one read contains a highly repetitive *k*-mer are extracted and clustered based on the sequence similarity between different read pairs. Each cluster is expected to contain reads that were derived from a single genomic regions

The targeted error correction pipeline has four main steps (Fig. 1). The first step is the *k*-mer selection procedure. Our experimental investigation shows that most of the breakpoints in the assembled contigs occur in the direct vicinity of low-complexity *k*-mers such as poly(A/T) or poly(C/G) (see Additional file 1: 3). There are two main reasons for this. Firstly, these *k*-mers are highly repetitive in the datasets. For example, the poly(A/T) 15-mer has the highest frequency among all 15-mers in 3 out of 6 datasets (see Additional file 1: 3). Such highly repetitive *k*-mers form hubs in the DBG through which a vast number of reads pass. They represent the central node in a densely connected subgraph of the DBG for which the resolution of the true path is very complex. Secondly, it has been observed in Illumina sequencing data that GC-rich or GC-depleted regions such as homopolymers are more prone to sequencing errors, especially insertions and deletions [36]. Fig. 1 in Additional file 1 shows that the average quality scores of reads that contain a poly(A/T) or poly(C/G) pattern are much lower than average. As such, those reads generally contain more sequencing errors than average. Particularly, in dataset D2, the average quality score of bases in reads that contain a poly(A/T) pattern is 20, whereas the average quality score for regular reads is 31. This means that a base of a read that contains a poly(A/T) sequence is about 10 times more likely to be erroneous than average. Therefore, it is very difficult for the assembler to establish a connection between reads in these regions which explains why the produced contigs by SPAdes often end with a poly(A/T) *k*-mer (see Table 2 in Additional file 1: 3). Reads with other kinds of low-complexity repeats appear less susceptible for an excessive number of sequencing errors. In this paper we correct only read pairs for which one of the reads contains a poly(A/T) 15-mer or longer. The effect of our proposed error correction procedure for other examples of low-complexity *k*-mers also has been investigated and the corresponding results are reported in Additional file 1.

**Fig. 2:**
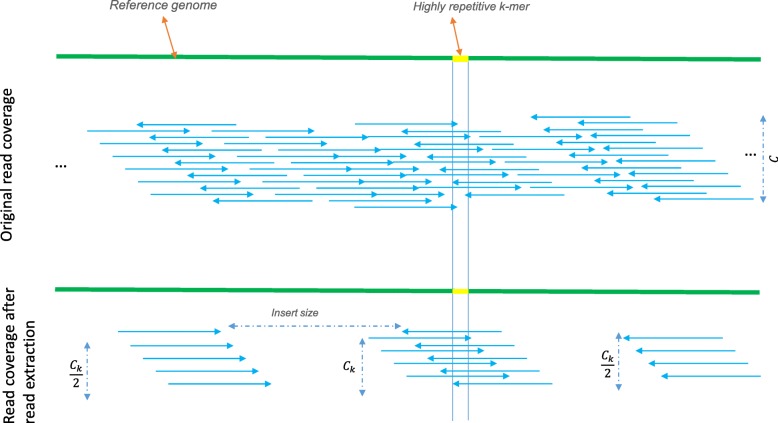
While *C* is the initial coverage (top), the expected number of reads that fully cover a selected *k*-mer is *C*_*k*_. Depending on the insert size and the insert size variability, the left and right flanking regions that are covered by the paired reads have a coverage of *C*_*k*_/2 or lower

The second step in the pipeline is the read extraction. Reads that contain a specific *k*-mer can easily be extracted in a single pass over the dataset. The expected number of reads that fully cover a *k*-mer occurrence in the genome can be computed as follows. Let *C* denote the coverage, i.e., the average number of reads that covers any base of that *k*-mer occurrence. Some of these reads will not cover the entire *k*-mer or might contain sequencing errors and hence they will not be extracted in this step. The expected number of extracted reads *C*_*k*_ that fully overlap a *k*-mer in the genome is given by $C_{k}=\frac {l-k+1}{l}C(1-e)^{k} $ where *l* is the read length and *e* denotes the error rate (see Additional file 1: 4). Reads are extracted in pairs and since these paired reads can occur on either side of the *k*-mer, the expected number of reads covering the flanking regions is *C*_*k*_/2. Due to fragment length (insert size) variability, these paired reads might be more spread out over the flanking regions. Figure 2 provides a schematic representation of the coverage distribution after read extraction where the expected number of pairs in one cluster is *C*_*k*_.

The third step in the pipeline is read pair clustering. The idea is to partition the read pairs into distinct clusters in such a way that all read pairs within a cluster originate from the same genomic region. The expected number of read pairs in each cluster is *C*_*k*_. BrownieCorrector uses the Louvain community detection algorithm, a very fast and memory efficient hierarchical graph clustering algorithm [27]. It is based on the greedy maximization of modularity and can handle large-scale networks (*N*>10^8^) [40]. The Louvain community detection algorithm takes as input a graph where nodes represent read pairs and where arcs between nodes represent the similarity score between read pairs. This similarity score is obtained by computing the pairwise overlap alignment score. The overlap alignment score represents the highest alignment score between a prefix of one sequence and the suffix of another, hence, trailing and leading gaps in the alignment are not penalized. Note that not only the sequence similarity between the two reads that contain the repetitive *k*-mer is taken in account, but also potential overlap between their respective paired reads. We found the information contained in the paired reads to be valuable to obtain robust and homogeneous clusters.

Computing the overlap alignment score between all pairs of reads has a quadratic time dependency on the number of read pairs and can hence be time-consuming for a large number of pairs. In BrownieCorrector, the read alignment score is only computed between read pairs that share at least one non-repeated *k*-mer, i.e., a *k*-mer for which the coverage is about *C*_*k*_. This heuristic avoids the computation of alignment scores between read pairs with apparent low sequence similarity. This also means that the input graph for the community clustering algorithm is generally very sparse.

The Louvain community detection algorithm outputs clusters for which the nodes in each cluster are densely connected while having only relatively few connections between nodes that belong to different clusters. The algorithm is non-deterministic which means that in every run, it may output different clusters. In order to reduce the impact of non-deterministic behavior of the algorithm and improve the robustness of the clusters, BrownieCorrector repeats the clustering procedure several times. The stable core communities are then established as explained in [28].

**Fig. 3:**
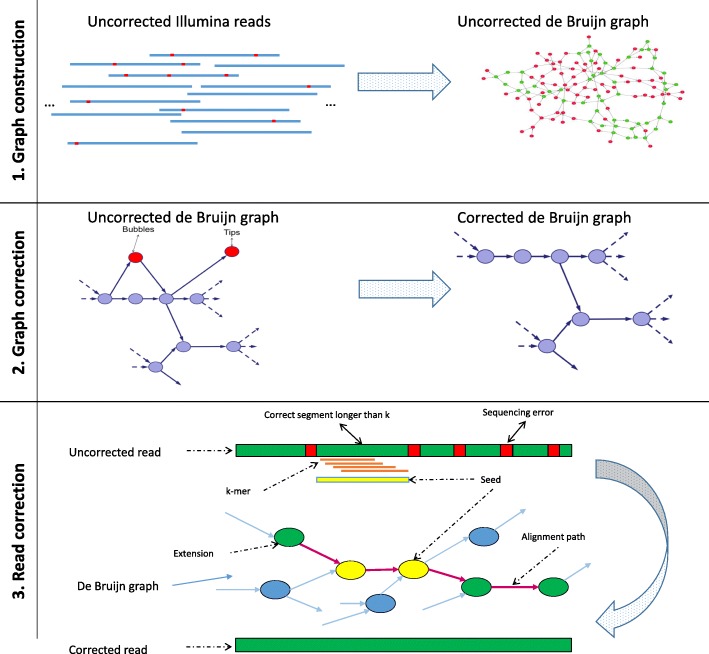
The final step of the BrownieCorrector pipeline: (1) de Bruijn Graph is built from the uncorrected reads in a cluster. Uncorrected reads contain sequencing errors which result in the appearance of erroneous *k*-mers and subsequently erroneous nodes/arcs in the graph; (2) erroneous nodes (colored in red) are detected and removed from the graph based on coverage and graph topology. Such erroneous nodes often appear as tips or bubbles; (3) reads are aligned individually to the corrected graph and mismatches and indels in the reads are detected and fixed with the correct path in the graph

The final step of the pipeline involves correcting the reads for each cluster independently. This step has three stages (see Fig. 3): i) construction of the DBG from input sequences; ii) correction of the DBG based on topology and coverage considerations; iii) correction of the input reads by aligning them to the corrected DBG.

**Fig. 4:**
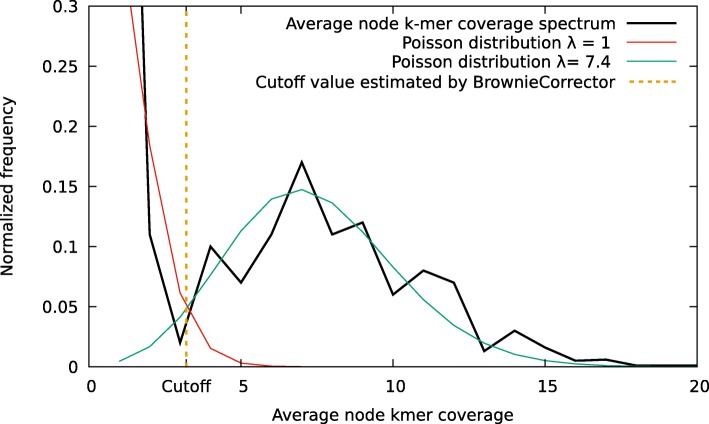
Real example of a *k*-mer frequency spectrum that is a superposition of two distributions corresponding to real and erroneous *k*-mers, respectively. A model of two Poisson distributions is fit to the data using the expectation-maximization algorithm. The coverage cutoff is established at the intersection of the two distributions

i) Reads are first assembled in a DBG. Given a user-specified value for *k*, all *k*-mers are extracted from the reads and a DBG is constructed. To reduce memory requirements, *k*-mers are encoded by 2*k* bits and stored in a memory-efficient hash map with only 2 bits overhead per entry. Overlap between *k*-mers is encoded by 8 bits: 4 bits to indicate if the *k*-mers can be left-extended with A, C, T or G and similarly 4 bits to represent right overlap. Linear paths in the graph are contracted to bigger nodes (unitigs) and various statistics such as length (number of *k*-mers in a node), average *k*-mer coverage (average number of reads that cover a *k*-mer in the node) are computed for each node.

ii) Whereas *k*-mer spectrum-based EC methods such as Quake identify errors based on (relative) *k*-mer abundances, erroneous nodes in the DBG are identified by BrownieCorrector based on graph topology and coverage considerations, as conceptually described by Zerbino and Birney [3]. For example, a true *k*-mer with a low abundance might be incorrectly classified as erroneous when judging solely on *k*-mer spectrum. By taking into account the context in which the *k-mer* occurs, it could, for example, become clear that this *k*-mer is part of a linear path in the DBG and that no parallel path exists with higher coverage. As such, the *k*-mer can be correctly classified as a true *k*-mer. Vice versa, an erroneous *k*-mer with a higher abundance can be detected because of topology considerations: either because the *k*-mer is part of a dead-end in the graph (a tip) or because it forms a path parallel to the correct sequence path. BrownieCorrector adopts a conservative, multi-round approach, avoiding the removal of true nodes as much as possible. A tip or a bubble is labeled as an erroneous node and will be removed if its length is less than the *maxErrorNodeLen* value and its average node *k*-mer coverage is less than the *cutoff* value. The value of *maxErrorNodeLen* is set to *avgReadLen*−*k* where *avgReadLen* is the average read length and *k* is the *k*-mer size. The histogram of average node *k*-mer coverage for all the nodes in DBG shows a mixture of two distributions: one that represents erroneous nodes and one that represents correct nodes (see Fig. 4). Using the expectation-maximization algorithm, a mixture of two Poisson distributions is fit: a distribution of erroneous nodes with mean *λ*_*e*_ and a distribution of correct nodes with mean *λ*_*c*_. BrownieCorrector computes the *k*-mer *cutoff* value at the intersection point of the two distributions.

iii) The original reads are aligned back to the corrected DBG using a seed-and-extend paradigm. In case a read contains at least one true *k*-mer, this *k*-mer is used as a seed that uniquely maps the read to a certain node in the DBG. A depth-first search on the graph is performed to align both ends of the read beyond the seed(s). Pairwise alignments are used to find the optimal alignment path. Branch-and-bound conditions are used to limit the search space. We refer to [29] for a more detailed description of the read-to-graph alignment procedure.

## Results

### Ability of EC tools to improve genome assembly

Table 2 shows the contig and scaffold NGA50 values for nine datasets and the different EC tools. The NGA50 denotes the characteristic length of the assembled contigs/scaffolds that can be contiguously aligned to the reference genome. These contigs/scaffolds thus contain no major structural assembly flaws and a higher NGA50 hence implies a better quality assembly. The first six columns show the assembly results for the Illumina datasets (D1 =R1,…, D6 =R6), while the last three columns refer to the hybrid assembly of Illumina and PacBio datasets (D7 =R4+P1, D8 =R5+P1 and D9 =R6+P2).

**Table 2:** NGA50 of respectively contigs (top) and scaffolds (bottom) assembled by SPAdes before and after error correction

Tools	D1	D2	D3	D4	D5	D6	D7	D8	D9
	Contig NGA50								
Uncorrected	10 876	5 451	6 325	50 833	35 924	40 802	80 752	85 003	65 138
ACE	11 375	8 475	3 116	29 126	20 032	34 273	55 391	65 163	62 161
BFC	11 672	9 488	6 307	49 089	27 365	40 910	77 526	78 985	64 709
BLESS2	9 183	7 737	2 969	25 133	17 133	29 968	61 609	60 574	55 639
BrownieCorrector	**13 334**	**11 015**	**6 328**	52 152	**38 670**	**45 400**	83 397	**88 877**	**71 788**
Karect	12 507	10 103	6 295	**54 106**	29 286	41 391	**85 226**	85 881	68 873
Reckoner	9 154	6 440	6 281	41 977	26 296	39 605	58 176	71 724	56 734
	Scaffold NGA50								
Uncorrected	11 377	5 668	6 419	60 714	59 591	41 833	96 381	109 785	84 659
ACE	12 135	8 597	3 143	35 425	40 860	39 895	62 981	93 602	83 138
BFC	12 294	9 698	6 392	59 124	54 093	41 818	91 577	110 748	82 101
BLESS2	10 034	7 909	3 012	34 856	36 316	38 431	73 377	86 526	74 447
BrownieCorrector	**14 155**	**11 570**	**6 420**	61 474	**65 174**	**46 678**	96 385	118 192	**96 916**
Karect	13 528	10 298	6 377	**63 400**	59 526	42 256	**101 753**	**124 215**	90 661
Reckoner	9 670	6 509	6 354	47 781	50 834	40 779	67 061	99 419	71 646

The best result for each dataset is shown in bold

Overall, BrownieCorrector shows the best performance and has the highest contig/scaffold NGA50 in 13 out of 18 cases while Karect has the highest NGA50 in the 5 remaining cases. In those cases, BrownieCorrector is second best. Pre-correcting reads with BrownieCorrector leads to improved assembly results in for all datasets. The other EC tools (ACE, BLESS 2, BFC and Reckoner) show mixed results. D2 is the only dataset for which all EC tools improve the contig/scaffold NGA50 over the use of uncorrected data. For datasets D3 and D5, all EC tools except BrownieCorrector deteriorate the assembly results. For some EC tools this leads to significantly shorter contigs/scaffolds. In 12 out of 18 cases, the contig and scaffold NGA50 values obtained from uncorrected data are among the top 3 highest values (though often below those of BrownieCorrector and Karect). It shows that SPAdes, which uses advanced paired and multi-sized de Bruijn graphs, uses accurate built-in error correction algorithms in the assembly process as well.

The results indicate that BrownieCorrector and Karect are the only reliable EC tools that perform consistently across different datasets. Table 3 shows the contig/scaffold NGA50 when applying both BrownieCorrector and Karect to the Illumina data. The idea is that BrownieCorrector first corrects only reads with a highly repetitive *k*-mer and that Karect corrects the other reads. We observe that the combined effect of the two error correction tools further raises the assembly quality except for the cases where Karect already performs poorly. This indicates that both tools are complementary to some degree. The improvements in NGA50 over the use of uncorrected data (averaged over all datasets) shows that the combined use of BrownieCorrector and Karect leads to the highest positive impact on the quality of contigs/scaffolds (+21%/+25%) while BrownieCorrector (+18%/+19%), Karect (+11%/+15%), and BFC (+5%/+7%) are the second, third and fourth best tool, respectively. On the other hand, BLESS2 (-25%/-19%), ACE (-17%/-14%) and Reckoner(-11%/-10%) deteriorate the quality of assembly on average (see Additional file 1: 5.1).

**Table 3:** NGA50 of respectively contigs (top) and scaffolds (bottom) assembled by SPAdes after error correction by both BrownieCorrector and Karect

Tools	D1	D2	D3	D4	D5	D6	D7	D8	D9
	Contig NGA50								
BrownieCorrector	13 334	11 015	**6 328**	52 152	**38 670**	45 400	83 397	**88 877**	71 788
Karect	12 507	10 103	6 295	54 106	29 286	41 391	85 226	85 881	68 873
Karect+BrownieCorrector	**13 526**	**12 409**	6 297	**56 046**	30 557	**45 423**	**89 065**	87 822	**74 620**
	Scaffold NGA50								
BrownieCorrector	14 155	11 570	**6 420**	61 474	**65 174**	**46 678**	96 385	118 192	96 916
Karect	13 528	10 298	6 377	63 400	59 526	42 256	101 753	124 215	90 661
Karect+BrownieCorrector	**14 613**	**12 795**	6 380	**65 857**	62 706	46 332	**103 872**	**126 449**	**104 037**

The best result for each dataset is shown in bold

We additionally investigated the impact of error correction by using other highly repetitive *k*-mers. This time a poly(C/G) pattern was utilized to extract the reads. The results show that correcting these reads with BrownieCorrector has a smaller impact on the assembly quality except for dataset D3 in which the NGA50 of both contigs and scaffolds is higher than the values in Table 2 (see Additional file 1: 5.2). This can be explained by the fact that for most datasets the poly(C/G) *k*-mer is much less frequent than poly(A/T) pattern and hence SPAdes benefits less from the error correction of those reads. The correction of reads with a poly(AC/GT) 15-mer does not lead to improved assemblies, even though a poly(AC/GT) 15-mer is more frequent than a poly(C/G). This is because reads that contain these poly (AC/GT) *k*-mers do not suffer from the error bias that can be observed in reads containing a poly(A/T) or poly(C/G) pattern. Finally, we examined the impact of the number of iterations in the stable core detection procedure on the final assembly result in D1. The default value for this parameter is 20, which is compared to 1 (when the stable core detection is disabled), 5, 10 and 30. The result shows that using the stable core improves the accuracy, however, BrownieCorrector is robust and performs good as well for other values (see Additional file 1: 5.3). The detailed Quast reports for all datasets and EC tools for both contigs and scaffolds are provided in Additional file 1: 5.4 and 5.5.

### Time and space requirements

**Fig. 5:**
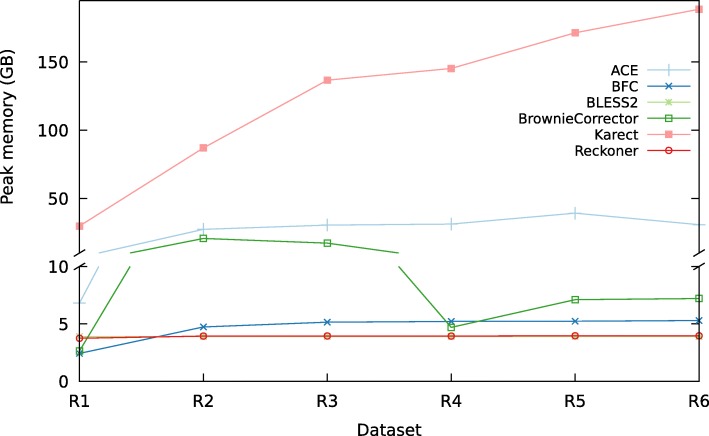
Peak memory usage. Peak memory usage of the EC tools

**Fig. 6:**
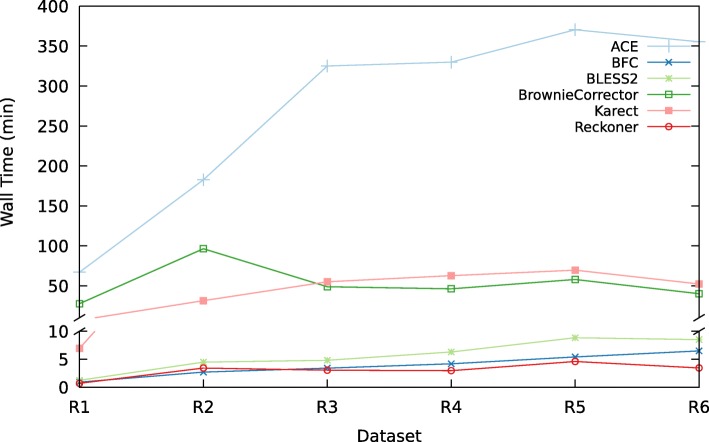
Runtime. Runtime of the EC tools

Figure 5 shows the memory usage of the EC tools (see Additional file 1: 5.6 for detailed tables). Reckoner, BLESS 2, and BFC are the most memory-efficient tools; memory usage of ACE and BrownieCorrector is comparable and Karect has the highest memory requirements. Figure 6 compares the runtime of the different EC tools for each dataset. Reckoner, BLESS 2, and BFC are the fastest tools whereas ACE, Karect, and BrownieCorrector are somewhat slower. Generally speaking, Reckoner, BLESS 2 and BFC are fast and memory efficient.

## Discussion

Although BrownieCorrector corrects only a small fraction of the reads (less than 2%, see Additional file 1: 5.2), results show that it performs well for a diverse set of organisms and even for relatively low coverage data (33×). The only parameter that can negatively affect the performance of BrownieCorrector is a larger standard deviation of fragment length (insert size). In that case, there is less overlap between paired reads and the identification of homogeneous clusters is more challenging. For example, BrownieCorrector performs worse than Karect in datasets D4 and D7 which is due to the fact that the standard deviation for the R4 Illumina dataset is 92, which is relatively high compared to the other datasets.

**Fig. 7:**
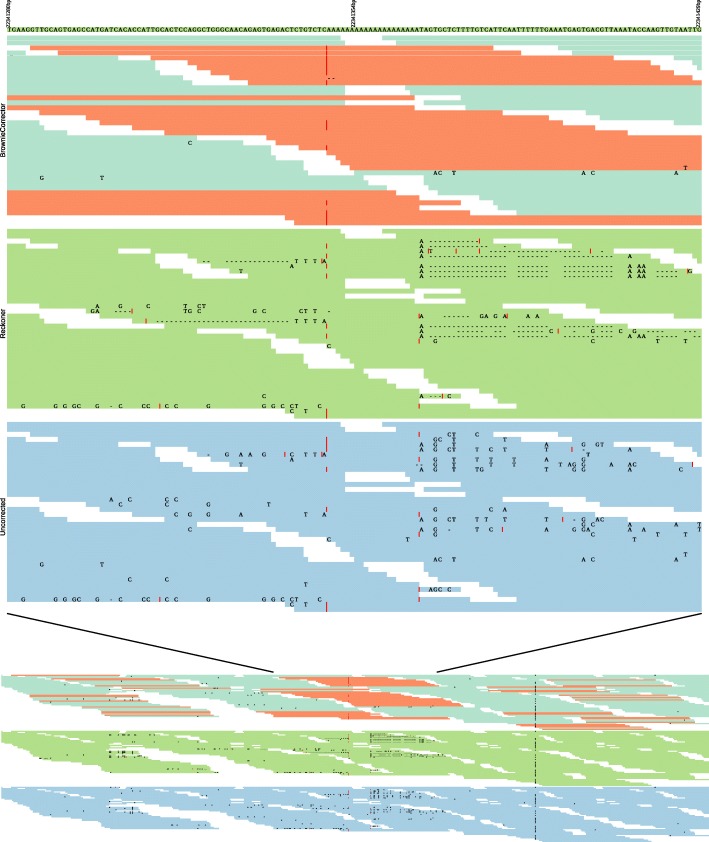
Alignment of BrownieCorrector-corrected, Reckoner-corrected and uncorrected paired reads in the neighborhood of a contig breakpoint: the first track contains part of the reference genome, which is assembled into a single contig from BrownieCorrector-corrected data but breaks into two contigs using Reckoner-corrected or uncorrected data. The second track (BrownieCorrector) shows the alignment of the BrownieCorrector-corrected reads. The only the reads in orange are corrected by BrownieCorrector. The third track (Reckoner) shows the alignment of the Reckoner-corrected reads. The fourth track (Uncorrected) shows the alignment of uncorrected reads. Mismatches in the sequencing data are indicated with letters whereas an insertion is shown with a ∣ sign and a deletion is shown with a - sign

The main advantage of BrownieCorrector over other tools lies in its use of paired-end read information. Figure 7 shows a specific case for dataset D1 where the use of BrownieCorrector resolves a breakpoint near a poly(A/T) pattern that occurs when using uncorrected or Reckoner-corrected data. To create this figure, all uncorrected reads were aligned to the reference genome using BWA and the read pairs that overlap the breakpoint were extracted. Next, the corresponding reads corrected by both BrownieCorrector and Reckoner were obtained. BrownieCorrector corrects only the reads that contain a poly(A/T) 15-mer (shown in orange). Although the average error rate in Illumina sequencing data is around (1-2%), we observe a much higher error rate in the vicinity of the poly(A/T) 15-mer. This is already confirmed by the low average quality scores of reads that contain poly(A/T) patterns (see Table 2 in Additional file 1). This high error rate renders SPAdes unable to correctly bridge the breakpoint. Also EC tools that do not exploit the paired-read information are likely to correct these highly erroneous reads in an inconsistent manner as exemplified for Reckoner. In contrast, using the paired reads, BrownieCorrector can still correctly cluster and correct these low-quality reads.

## Conclusions

We propose BrownieCorrector, a targeted error correction tool that corrects Illumina sequencing errors in paired-end reads that contain highly-repetitive patterns such as short homopolymers. Such reads form densely connected subnetworks in the de Bruijn graph, which, in the presence of sequencing errors, are difficult to resolve, ultimately leading to a fragmented assembly. BrownieCorrector uses the entire read sequence as well as the paired-end read information to cluster read pairs in homogeneous groups, where the paired-end reads in each group originate from the same genomic region. Reads in each cluster are corrected independently such that a consistent correction is achieved for all reads within each cluster. Despite the fact that BrownieCorrector corrects only a small fraction of the input reads, results indicate it outperforms other error correction tools in terms of contiguity of the assembled contigs and scaffolds. This observation lends support to the idea that error correction tools should focus their efforts on the correction of ‘difficult’ sequencing errors. Indeed, the utility of error correction tools lies in their ability to improve the quality of downstream applications. We believe that for future EC tools, it is ultimately more beneficial to try and correct problematic regions really well, rather than designing a method that performs well across the entire genome but fails to produce consistent corrections for certain regions. By limiting the application of these algorithms, which perhaps need more CPU cycles, to these specific regions, the computational cost can still be kept under control. Such algorithms likely need to exploit the paired-end read information to ensure a consistent error correction.

We also investigated the impact of BrownieCorrector in a hybrid genome assembly setup where Illumina sequencing data is combined with PacBio data. Our results show that the use of BrownieCorrector-corrected Illumina reads along with PacBio data leads to better assembly results in this case as well. One of the advantages of BrownieCorrector’s pipeline is its modularity where each step can be replaced by a method of choice. For example, the Louvain community detection algorithm can easily be replaced by another clustering algorithm, other EC tools can be used to correct clusters or different metrics can be used to infer the similarity score between pairs of reads. We believe this flexibility allows the pipeline to further evolve in the future.

## Additional file

Additional file 1 Targeted Error Correction Improves the Assembly Results. (PDF 1261 kb)

## Abbreviations

bp: Base pair
DBG: De Bruijn graph
EC: Error correction
GB: Gigabyte
GHz: Gigahertz
Mbp: Megabase pair
