# Supplementary material for: Illumina error correction near highly repetitive DNA regions improves de novo genome assembly
Source: BMC Bioinformatics. 2019 Jun 3;20:298. doi: 10.1186/s12859-019-2906-2 (PMC6545690; doi:10.1186/s12859-019-2906-2)
Supplement: Supplementary file 1 — Targeted Error Correction Improves the Assembly Results. (PDF 1261 kb) [file 12859_2019_2906_MOESM1_ESM.pdf]

Illumina error correction near highly repetitive DNA regions improves de novo genome assembly

Mahdi Heydari[1,2], Giles Miclotte[1,2],
Yves Van de Peer[2,3,4,5], and Jan Fostier[1,2]

[1]Department of Information Technology, Ghent University-imec, IDLab, Ghent, Belgium
[2]Bioinformatics Institute Ghent, Ghent, Belgium
[3]Center for Plant Systems Biology, VIB, Ghent, Belgium
[4]Department of Plant Biotechnology and Bioinformatics, Ghent University, Ghent, Belgium
[5]Department of Genetics, Genome Research Institute, University of Pretoria, Pretoria, South Africa

Contact: jan.fostier@ugent.be

Contents

1 Parameter settings **4**
- 1.1 ACE . . . 4
- 1.2 BFC . . . 4
- 1.3 BLESS2 . . . 4
- 1.4 Browniecorrector . . . 4
- 1.5 Karect . . . 4
- 1.6 RECKONER . . . 5
- 1.7 SPAdes . . . 5
- 1.8 Quast . . . 5

2 Data preparation **5**
- 2.1 Illumina real data . . . 5
- 2.2 Pacbio real data . . . 6

3 *k*-mer selection **6**

4 *k*-mer coverage **10**

5 Results **10**
- 5.1 Average improvement ratio of NGA50 . . . 10
- 5.2 Choice of highly repetitive *k*-mer . . . 10
- 5.3 Choice of the number of iterations . . . 11
- 5.4 Full Quast report (contigs) . . . 15
  - 5.4.1 D1 . . . 16
  - 5.4.2 D2 . . . 18
  - 5.4.3 D3 . . . 20
  - 5.4.4 D4 . . . 22
  - 5.4.5 D5 . . . 24
  - 5.4.6 D6 . . . 26
  - 5.4.7 D7 . . . 28
  - 5.4.8 D8 . . . 30
  - 5.4.9 D9 . . . 32
- 5.5 Full Quast report (scaffolds) . . . 34
  - 5.5.1 D1 . . . 35
  - 5.5.2 D2 . . . 37
  - 5.5.3 D3 . . . 39
  - 5.5.4 D4 . . . 41
  - 5.5.5 D5 . . . 43
  - 5.5.6 D6 . . . 45
  - 5.5.7 D7 . . . 47
  - 5.5.8 D8 . . . 49
  - 5.5.9 D9 . . . 51

5.6 Runtime and memory usage 53

1 Parameter settings

Tools were executed with 64 threads. BLESS2 fails to finish with 64 threads in some datasets; therefore, only 32 cores were used by this tool. For all tables and figures in the main paper and in the supplementary data the parameters' default or recommended values were selected for each tool. Below, the command line parameters are specified for each tool individually:

1.1 ACE [1]

```
$ size=$(stat -c%s genome.fasta)
$ ./ace $size $inputreads aceOut/aceCorrected
```

1.2 BFC v. r181 [2]

```
$ size=$(stat -c%s genome.fasta)
$ ./bfc -s $size -k 33 -t 64 $inputreads >bfcOut/bfcCorrected
```

1.3 BLESS2 v. 1.02 [3]

```
$ ./bless -read $inputreads -prefix blessOut/blessCorrected
-kmerlength 31 -smpthread 32
```

1.4 Browniecorrector v. 1.0 [4]

Arguments need to be provided in the following order: the first argument is the data library (note that if there are multiple libraries, each library must be corrected individually); The second argument is the *cov* which is the depth of coverage in that library; the last argument is the working directory.

```
$  ./bashScripts/runPipeLine.sh $inputreads $cov $workdir
```

1.5 Karect v. 1.0 [5]

```
$ ./karect -correct -inputfile=$inputreads -matchtype=hamming
-celltype=diploid -resultdir=karectOut -kmer=9 -memory=32 -threads=32
```

[1]https://github.com/Sheikhizadeh/ACE.git
[2]https://github.com/lh3/bfc.git
[3]https://sourceforge.net/projects/bless-ec
[4]https://github.com/biointec/browniecorrector.git
[5]https://github.com/aminallam/karect.git

1.6 RECKONER v. 1.1.1 [6]

```
$ size=$(stat -c%s genome.fasta)
$ reckoner -prefix reckonerOUt -threads 64 -read $inputreads -genome $size
```

1.7 SPAdes v. 4.12[7]

In order to see the impact of EC tools on assembly results, we used SPAdes to assemble both corrected and uncorrected data. The following command was used to run the SPAdes.

```
spades.py -t 32 --only-assembler --12 reads.fastq -o outputDir
```

1.8 Quast v. 4.6.3[8]

Quast provides comprehensive information on the assembly quality. The following command was used to run the Quast.

```
./quast.py asmDir/contigs.fa -R genome.fasta -o quastReport --plots-format
pdf --labels "toolName"
```

2 Data preparation

2.1 Illumina real data

1. R1 (*Homo sapiens* Chr. 21)
   Download[9] the row data from :Human_NA19240.7z
   ```
   $ 7z e Human_NA19240.7z
   $ shuffleSequences_fastq.pl NA19240_HiSeq_100_chr21_R1_paired.fq NA19240_HiSeq_100_chr21_R2_paired.fq
   reads.fastq
   ```
   Download the reference genome from hs_ref_GRCh38.p12_chr21.fa.gz

2. R2 (*Homo sapiens* Chr. 14)
   Download the row data from frag_1.fastq.gz, and frag_2.fastq.gz
   ```
   $ gzip -d frag_1.fastq.gz
   $ gzip -d frag_2.fastq.gz
   $ shuffleSequences_fastq.pl frag_1.fastq frag_2.fastq reads.fastq
   ```
   Download the reference genome from genome.fasta

3. R3 (*Caenorhabditis elegans*)
   Download the row data from : SRR543736.sra
   ```
   $ fastq-dump --split-files SRR543736.sra
   ```

[6]https://github.com/refresh-bio/RECKONER.git
[7]http://cab.spbu.ru/software/spades/
[8]http://quast.sourceforge.net/quast
[9]In case you have any difficulties of downloading the files, you can open this document with "document viewer" application, copy the link and paste it in your browser.

```
$ shuffleSequences_fastq.pl SRR543736_1.fastq SRR543736_2.fastq reads.fastq
```
Download the reference genome from c_elegans.WS222.genomic.fa.gz

4. R4 (*Drosophila melanogaster*) Download the row data from SRR823377.sra
```
$ fastq-dump --split-files SRR823377.sra
$ shuffleSequences_fastq.pl SRR823377_1.fastq SRR823377_2.fastq reads.fastq
```
Download the reference genome via the following links: NT_033777.fna, NT_033778.fna, NT_033779.fna, NT_037436.fna, NC_004353.fna and NC_004354.fna. Then concatenate them all together:
```
$ cat NT_033777.fna NT_033779.fna NT_033778.fna NT_037436.fna NC_004353.fna NC_004354.fna > genome.fasta
```

5. R5 (*Drosophila melanogaster*) Download the row data from SRR988075.sra
```
$ fastq-dump --split-files SRR988075.sra
$ shuffleSequences_fastq.pl SRR988075_1.fastq SRR988075_2.fastq reads.fastq
```
The reference genoem is the same as R4.

6. R6 (*Arabidopsis thaliana*) Download the row data from SRR988075.sra
```
$ fastq-dump --split-files SRR1174256.sra
$ shuffleSequences_fastq.pl SRR1174256_1.fastq SRR1174256_2.fastq reads.fastq
```
Download the reference genome from GCF_000001735.4_TAIR10.1_genomic.fna.gz

2.2 Pacbio real data

1. P1 (*Drosophila melanogaster*)
Download the row data from SRR1204466.sra
```
$ fastq-dump --split-files SRR1204466.sra
$ cat SRR1204466*.fastq >pacbio.reads.fastq
```

2. P2 (*Arabidopsis thaliana*)
Download the row data from SRR1284707.sra
```
$ fastq-dump --split-files SRR1284707.sra
$ cat SRR1284707*.fastq >pacbio.reads.fastq
```

3 *k*-mer selection

Table 1 reperesents the most frequent k-mers in each dataset. For example a poly-(A/T) 15-mer is the most frequent 15-mer in 3 datasets. We used jellyfish to count the frequency of 15-mers in the datasets as follows:
```
$ jellyfish count -m 15 -s 100M -t 64 -C reads.fastq
$ jellyfish dump mer_counts.jf -L $threshold > kmerFile.high.fasta
```
The threshold values are chosen appropriately based on the dataset size to find the top-5 most frequent 15-mers.

Fig 1 shows the average quality score of bases in reads for different polymers and a group of randomly sampled reads. This picture shows that reads contain a poly (A/T) or (C/G) have a lower quality hence they are more erroneous.

Table 1: The top-5 most frequent 15-mers in each dataset.

Rank	15-mer	Frequency
**R1**		
1	AAAAAAAAAAAAAAA	843835
2	ACACACACACACACA	265661
3	AATGGAATGGAATGG	134236
4	AAAGTGCTGGGATTA	134174
5	CATTCCATTCCATTC	133799
**R2**		
1	AAAAAAAAAAAAAAA	2199579
2	ACACACACACACACA	703103
3	GCCTGTAATCCCAGC	459711
4	AGCACTTTGGGAGGC	432208
5	AAAGTGCTGGGATTA	431474
**R3**		
1	AAAAAAAAAAAAAAA	1105265
2	ATATTTTACTCTCTG	954488
3	ACTCTCTGTGGCTTC	755111
4	AAGCCACAGAGAGTA	754801
5	CCACAGAGAGTAAAA	746402
**R4**		
1	AATAACATAGAATAA	5590029
2	GAATAACATAGAATA	5549407
3	ATAACATAGAATAAC	5503200
4	CATAGAATAACATAG	5201994
5	AAGAGAAGAGAAGAG	5135751
**R5**		
1	AAGAGAAGAGAAGAG	16685329
2	ATAGAATAACATAGA	12988779
3	AACACAACACAACAC	12987799
4	AATAACATAGAATAA	9427421
5	ATAACATAGAATAAC	9277417
**R6**		
1	ACTCCAAAACACTAA	1831402
2	CCATGAAAGCTTTGA	1804271
3	AAAAAAAAAAAAAAA	1804010
4	CTCCAAAACACTAAC	1802058
5	TATGATTGAGTATAA	1794787

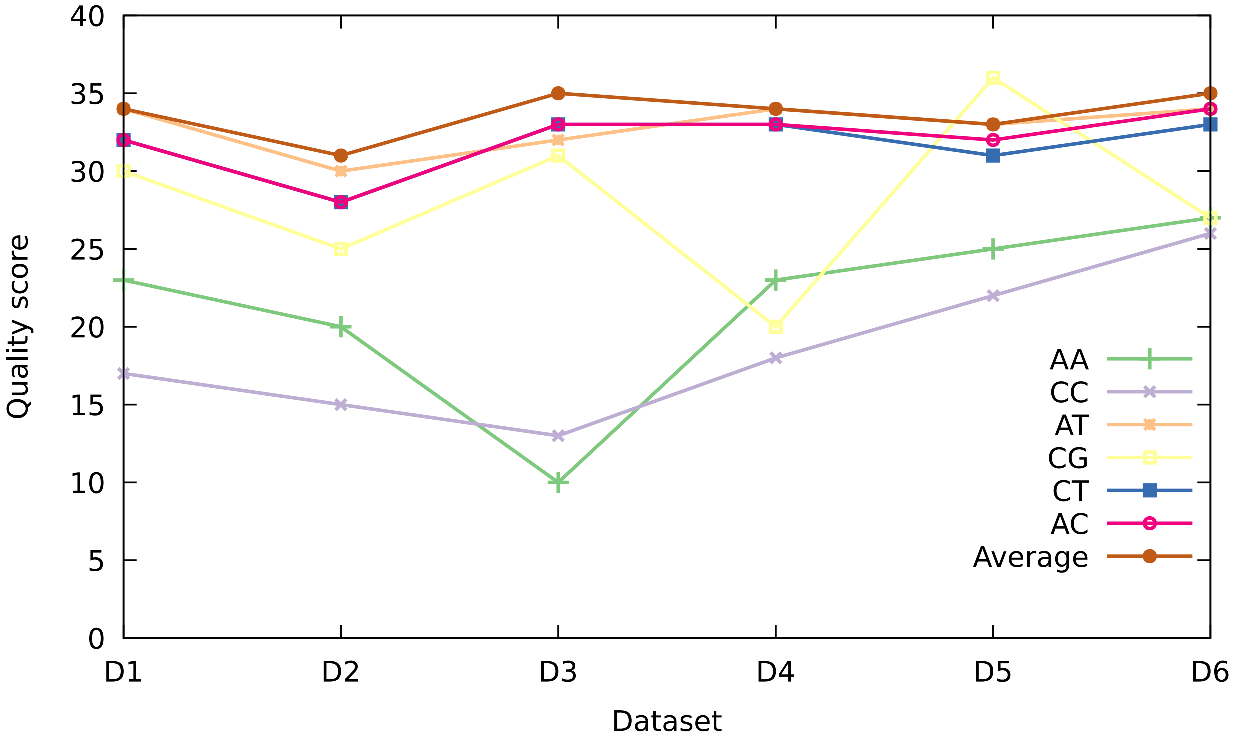

Figure 1: The average quality score of bases in reads for different polymers and a group of randomly sampled reads.

Our experimental investigations show that most of the breakpoints in the assembly results occur in the direct vicinity of highly repetitive k-mers. For example, the top 5 most frequent 15-mers in the first or last 100 bp of the assembled contigs ($>$ 1000bp) with SPAdes in 6 different datasets are listed in table 2:

Table 2: The top-5 most frequent 15-*mers* in the beginning or end of assembled contigs in different dataset.

Rank	15-mer	Frequency
	R1	
1	TTTTTTTTTTTTTTT	1853
2	TTTTTTTTTTTTTTG	1319
3	AAAAAAAAAAAAAAG	1164
4	AGCACTTTGGGAGGC	933
5	CCAGCACTTTGGGAG	891
	R2	
1	TTTTTTTTTTTTTTT	5609
2	TTTTTTTTTTTTTTG	4124
3	AGCACTTTGGGAGGC	3825
4	AATCCCAGCACTTTG	3664
5	AAAGTGCTGGGATTA	3658
	R3	
1	GGGGGGGGGGGGGGG	1276
2	GGGGGGGGGGGGGGA	617
3	CCCCCCCCCCCCCCA	596
4	TTTTTTTTTTTTTTT	500
5	TTCCCCCCCCCCCCC	387
	R4	
1	GGGGGGGGGGGGGGG	904
2	TTTTTTTTTTTTTTT	496
3	CCCCCCCCCCCCCCA	494
4	ATATATATATATATA	397
5	AGGGGGGGGGGGGGG	363
	R5	
1	TTTTTTTTTTTTTTT	1093
2	GGGGGGGGGGGGGGG	758
3	TTTTTTTTTTTTTTG	720
4	CCCCCCCCCCCCCCA	434
5	AAAAAAAAAAAAAAT	324
	R6	
1	TTTTTTTTTTTTTTT	1285
2	TTTTTTTTTTTTTTG	607
3	AAAAAAAAAAAAAAG	506
4	ATATATATATATATA	484
5	AAAAAAAAAAAAAAT	470

4 k-mer coverage

Assuming a genomic region and a randomly selected k-mer from this region, the average number of reads that initially cover any base of that k-mer is the initial coverage (C). The expected number of extracted reads from that region that contain that specific k-mer (C_k) is given by the following formula: $C_k = \frac{l-k+1}{l}C(1-e)^k$
where l is the read length and e is the error rate.
Proof:
It has been shown in [1], in the absence of errors the expected coverage of reads in a region of size k is $\frac{l-k+1}{l}C$. However, in the presence of errors, some of these reads fail to cover that region perfectly (i.e without any mismatch) due to the sequencing error. Let us assume the errors occur independently from each other, then the probability that all the bases of a read in an interval of size k are error-free is $(1-e)^k$. Therefore, the expected number of reads that cover a region of k is:
$C_k = \frac{l-k+1}{l}C(1-e)^k$.

5 Results

5.1 Average improvement ratio of NGA50

Table 2 and 3 in the main paper show the exact values of NGA50 for contigs and scaffods after and before the error correction. Table 3 shows the improvement rate of NGA50 for both contigs and scaffolds upon the uncorrected data for different datasets. The average improvement rate (AVG column) shows that jointly using of BrownieCorrector and Karect leads to the highest positive impact on the quality of contigs/scaffolds (+21%/+25%) whereas BrownieCorrector with (+18%/+19%), Karect with (+11%/+15%), and BFC with (+5%/+7%) are the second, third and forth best tools. On the other hand, BLESS2 (-25%/-19%), ACE (-17%/-14%), and Reckoner(-11%/-10%) deteriorate the quality of assembly on average.

5.2 Choice of highly repetitive k-mer

In order to see the performance of BrownieCorrector, we run the benchmark with two homopolymers poly-(A/T) and poly-(C/G). The results for poly-(A/T) are reported in the main paper. Table 4 compares the number of reads in each dataset that contains specific k-mers and respectively corrected versus the total number of reads in that dataset.

Table 5 and 6 show the NGA50 of contigs and scaffolds when reads that contain respectively a 15-mer poly (C/G) and a 15-mer poly (AC/GT) are corrected by BrownieCorrector. Table 5 indicates that correcting reads that contain a poly (C/G) often has a lower impact on the quality of the assembly (except D3 which yields in a higher NGA50). This is due to the fact that a poly C is less occurred than a poly A in all the datasets and the assembler can itself handle the associated complexity. Table 6 indicates that correcting reads that contain a poly (AC/GT) has no positive impact on the quality of the assembly and sometimes the results are slightly worse. This is due to the fact that even though poly (AC/GT) is frequent, but the quality of reads that contain a poly

Table 3: The improvement rate of NGA50 values for contigs and scaffolds upon the uncorrected data for different EC tools

Tools	D1	D2	D3	D4	D5	D6	D7	D8	D9	AVG
	Contig NGA50 improvement rate(%)									
ACE	5	55	-51	-43	-44	-16	-31	-23	-5	-17
BFC	7	74	0	-3	-24	0	-4	-7	-1	5
BLESS2	-16	42	-53	-51	-52	-27	-24	-29	-15	-25
BrownieCorrector	23	102	0	3	8	11	3	5	10	18
Karect	15	85	0	6	-18	1	6	1	6	11
Reckoner	-16	18	-1	-17	-27	-3	-28	-16	-13	-11
BrownieCorrector+Karect	24	128	0	10	-15	11	10	3	15	21
	Scaffold NGA50 improvement rate(%)									
ACE	7	52	-51	-42	-31	-5	-35	-15	-2	-14
BFC	8	71	0	-3	-9	0	-5	1	-3	7
BLESS2	-12	40	-53	-43	-39	-8	-24	-21	-12	-19
BrownieCorrector	24	104	0	1	9	12	0	8	14	19
Karect	19	82	-1	4	0	1	6	13	7	15
Reckoner	-15	15	-1	-21	-15	-3	-30	-9	-15	-10
BrownieCorrector+Karect	28	126	-1	8	5	11	8	15	23	25

(AC/GT) is high, and the assembler can itself handle the associated complexity. We highly suggest the user to use the poly A which is the default k-mer in the software.

5.3 Choice of the number of iterations

In order to find the stable cores of clusters we repeated the clustering multiple times. The default value for the number of iteration is set to 20 in the software. However, we further investigate the quality of assembly results (for D1) when it is set to 1, 5, 10 and 30 as well. Fig. 2 shows how NGA50 of contigs and scaffolds changes for different values of iteration. This picture indicates that using the stable cores after running the clustering multiple times improves the quality of assembly. However, it also shows the accuracy of BrownieCorrector is not much affected by changing this parameter in the range of (5 to 30).

Table 4: Two highly repetitive k-mers used in this study. The number of corrected and total number of reads in each dataset is compared.

highly repetitive k-mer	Number of corrected reads	Total number of reads
	R1	
AAAAAAAAAAAAAAA	264 608 (1.96%)	13 486 136
CCCCCCCCCCCCCCC	12 180 (0.09%)	13 486 136
ACACACACACACACA	96 542 (0.71%)	13 486 136
	R2	
AAAAAAAAAAAAAAA	620 500 (1.69%)	36 504 800
CCCCCCCCCCCCCCC	41 890 (0.11%)	36 504 800
ACACACACACACACA	202 770 (0.55%)	36 504 800
	R3	
AAAAAAAAAAAAAAA	198 598 (0.34%)	57 721 732
CCCCCCCCCCCCCCC	112 908 (0.19%)	57 721 732
ACACACACACACACA	72 848 (0.12%)	57 721 732
	R4	
AAAAAAAAAAAAAAA	576 552 (0.91%)	63 014 762
CCCCCCCCCCCCCCC	138 976 (0.22%)	63 014 762
ACACACACACACACA	477 950 (0.75%)	63 014 762
	R5	
AAAAAAAAAAAAAAA	653 028 (0.85%)	75 938 276
CCCCCCCCCCCCCCC	83 506 (0.10%)	75 938 276
ACACACACACACACA	486 066 (0.64%)	75 938 276
	R6	
AAAAAAAAAAAAAAA	571 806 (0.61%)	93 429 346
CCCCCCCCCCCCCCC	8 256 (0.01%)	93 429 346
ACACACACACACACA	32 320 (0.03%)	93 429 346

Table 5: NGA50 of respectively contigs (top) and scaffolds (bottom) assembled by SPAdes before and after error correction. Reads that contain a 15-mer poly (C/G) are corrected by BrownieCorrector.

Tools	D1	D2	D3	D4	D5	D6	D7	D8	D9
				Contig NGA50					
Uncorrected	10 876	**5 451**	6 325	**50 833**	35 924	40 802	**80 752**	85 003	65 138
BrownieCorrector	10 876	5 449	**6 438**	50 733	**36 177**	**40 805**	79 151	85 003	**65 469**
				Scaffold NGA50					
Uncorrected	11 377	5 668	6 419	**60 714**	59 591	41 833	**96 381**	109 785	84 659
BrownieCorrector	**11 385**	5 668	**6 525**	59 130	**60 500**	**41 836**	92 852	**110 560**	84 659

Table 6: NGA50 of respectively contigs (top) and scaffolds (bottom) assembled by SPAdes before and after error correction. Reads that contain a 15-mer poly (AC/TG) are corrected by BrownieCorrector.

Tools	D1	D2	D3	D4	D5	D6	D7	D8	D9
				Contig NGA50					
Uncorrected	**10 876**	**5 451**	**6 325**	50 833	**35 924**	**40 802**	**80 752**	**85 003**	**65 138**
BrownieCorrector	10 762	5 391	**6 325**	**50 834**	35 389	**40 802**	80 712	84 581	**65 138**
				Scaffold NGA50					
Uncorrected	**11 377**	**5 668**	**6 419**	**60 714**	59 591	41 833	**96 381**	**109 785**	**84 659**
BrownieCorrector	11 270	5 621	**6 419**	**60 714**	**59 827**	**41 836**	96 165	108 752	**84 659**

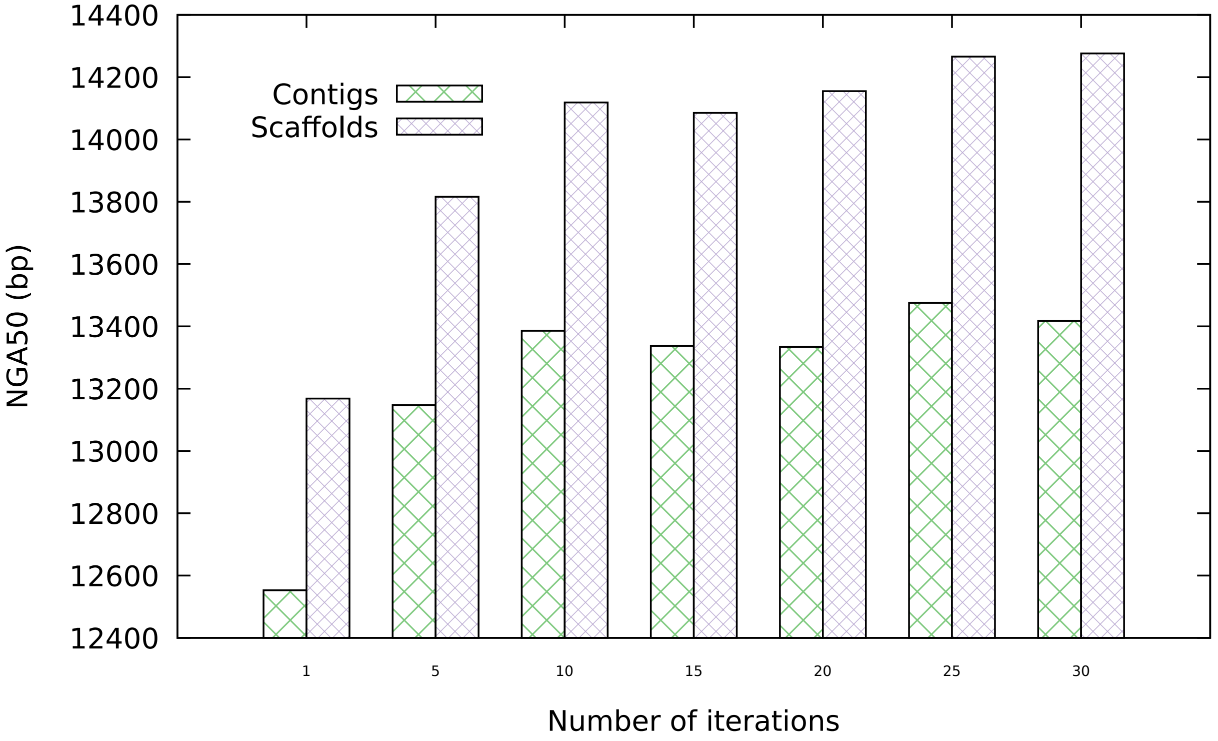

Figure 2: The impact of changing the number of iterations in reads clustering on the quality of assembly in D1(*Homo sapiens* chr. 21).

5.4 Full Quast report (contigs)

This section contains the Quast evaluation report of contigs after assembling each dataset with SPAdes. Error correction by ACE, BFC, BLESS2, Brownie, Karect and Reckoner is performed prior to assembling the reads. The Uncorrected column refers to the quality of contigs without any pre-correction process. The Hybrid column shows the quality of assembly of reads which are corrected jointly by BrownieCorrector and Karect. Default parameter settings are used for Quast, therefore all statistics are based on contigs of size $\geq$ 500 bp.

5.4.1 D1

Table 7 contains the Quast report after assembling dataset D1 (*Homo sapiens* Chr. 21) with SPAdes and Fig. 3 shows the corresponding NGAx plot.

Table 7: Assembly quality metrics for D1

Assembly	Uncorrected	ACE	BFC	BLESS2	Hybrid	Karect	BrownieCorrector	Reckoner
# contigs (≥ 0 bp)	15698	17387	15294	18776	16438	17247	14537	21465
# contigs (≥ 1000 bp)	3637	3501	3428	4065	3035	3269	3058	4218
# contigs (≥ 5000 bp)	1989	1953	1910	2065	1804	1898	1802	2060
# contigs (≥ 10000 bp)	1128	1115	1113	1064	1130	1126	1124	1082
# contigs (≥ 25000 bp)	239	254	271	189	314	281	321	181
# contigs (≥ 50000 bp)	24	24	28	11	46	34	43	11
Total length (≥ 0 bp)	34383421	34462802	34371704	34340929	34484783	34541537	34323631	**34746509**
Total length (≥ 1000 bp)	32610876	32607223	32648129	32311651	32677840	32667335	**32678726**	32480570
Total length (≥ 5000 bp)	28418343	28545565	28787065	27096397	**29533788**	29142408	29499002	26912697
Total length (≥ 10000 bp)	22265626	22534041	23076297	19919516	**24659630**	23596575	24593042	19861301
Total length (≥ 25000 bp)	8482791	9017654	9921318	6494787	11802375	10261128	**12015621**	6201468
Total length (≥ 50000 bp)	1439339	1504015	1767700	698377	**2815229**	2121583	2742260	674634
# contigs	4209	4018	3990	4674	3520	3776	3540	4874
Largest contig	82702	98568	**109124**	82889	88924	92853	98722	80324
Total length	33019875	32976721	**33047634**	32752359	33022607	33030548	33022956	32950991
Reference length	40988574	40988574	40988574	40988574	40988574	40988574	40988574	40988574
GC (%)	40.73	40.75	40.73	40.67	40.76	40.75	40.75	40.62
Reference GC (%)	40.93	40.93	40.93	40.93	40.93	40.93	40.93	40.93
N50	15054	15720	16310	12767	18515	17064	**18523**	12479
NG50	11384	11943	12348	9548	**13981**	12994	13969	9475
N75	7816	8068	8352	6681	**9864**	8990	9813	6589
NG75	2807	2990	2929	2365	**3519**	3269	3491	2377
L50	659	638	603	753	534	587	**532**	780
LG50	963	929	881	1123	781	854	**779**	1148
L75	1411	1361	1300	1629	**1141**	1251	1142	1679
LG75	2596	2509	2412	3101	**2093**	2291	2101	3146
# misassemblies	172	146	185	172	109	**100**	139	145
# misassembled contigs	163	134	168	163	102	**90**	130	140
Misassembled contigs length	2744756	2266007	3413239	2579081	2163845	2066353	2696928	**2019536**
# local misassemblies	117	**87**	100	93	89	90	114	93
# unaligned mis. contigs	0	0	0	0	0	0	0	0
# unaligned contigs	69 + 7 part	60 + 7 part	72 + 7 part	**47 + 6 part**	65 + 6 part	63 + 4 part	70 + 7 part	64 + 5 part
Unaligned length	75440	69319	81177	**52669**	70161	68430	76887	67237
Genome fraction (%)	80.057	79.980	80.098	79.544	**80.222**	80.191	80.200	79.862
Duplication ratio	1.004	1.004	1.004	1.003	**1.002**	1.003	**1.002**	1.005
# N's per 100 kbp	0.00	0.00	0.00	0.00	0.00	0.00	0.00	0.00
# mismatches per 100 kbp	166.59	163.62	161.90	162.96	**152.72**	155.00	157.31	158.16
# indels per 100 kbp	36.21	36.13	35.63	34.84	35.83	35.64	36.50	**33.08**
Largest alignment	82666	98563	96583	80972	85895	92848	**98722**	80324
Total aligned length	32887394	32846124	32913718	32658156	**32929390**	32922661	32921082	32800027
NA50	14357	15191	15483	12278	**17934**	16481	**17934**	12005
NGA50	10876	11375	11672	9183	**13526**	12507	13334	9154
NA75	7513	7758	7898	6399	**9446**	8584	9312	6292
NGA75	2672	2751	2743	2221	**3382**	3136	3344	2211
LA50	687	662	634	781	**552**	607	553	804
LGA50	1006	966	931	1168	**808**	883	812	1185
LA75	1477	1419	1375	1696	**1185**	1294	1194	1739
LGA75	2738	2629	2562	3245	**2178**	2376	2204	3280

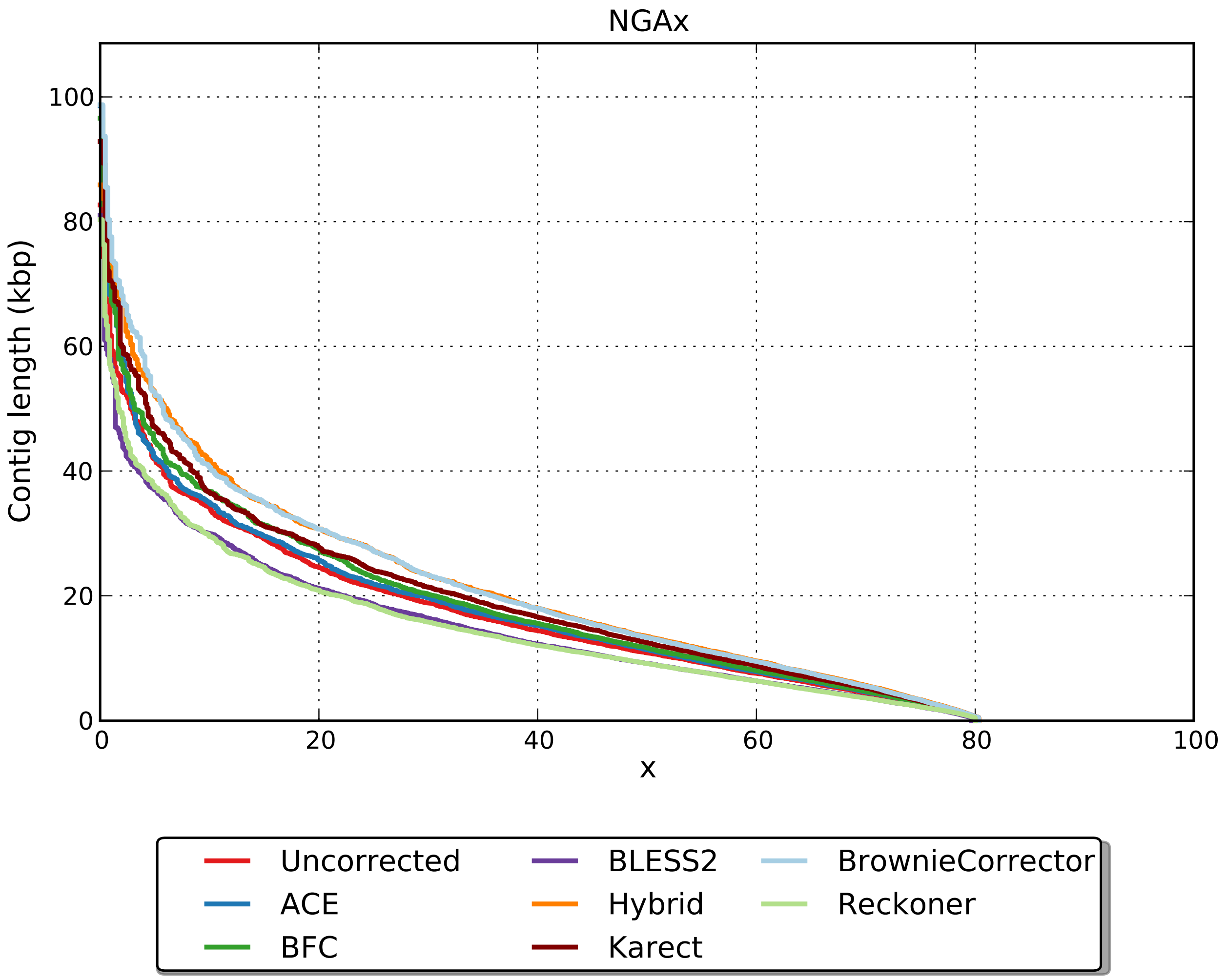

Figure 3: SPAdes assembly results for dataset D1 (*Homo sapiens* Chr. 21) for both uncorrected and corrected data. Contigs with length NGAx or larger produce x% of the genome.

5.4.2 D2

Table 8 contains the Quast report after assembling dataset D2 (*Homo sapiens* Chr. 14) with SPAdes. Fig. 4 shows the corresponding NGAx plot.

Table 8: Assembly quality metrics for D2

Assembly	Uncorrected	ACE	BFC	BLESS2	Hybrid	Karect	BrownieCorrector	Reckoner
# contigs (≥ 0 bp)	412807	64078	61489	65782	55134	59219	86196	83030
# contigs (≥ 1000 bp)	15216	10449	9716	11408	7780	9167	8834	13090
# contigs (≥ 5000 bp)	5457	5259	5117	5438	4672	5027	5035	5486
# contigs (≥ 10000 bp)	2048	2692	2771	2610	2797	2735	2785	2406
# contigs (≥ 25000 bp)	165	493	589	388	787	642	665	286
# contigs (≥ 50000 bp)	3	40	56	25	123	68	78	13
Total length (≥ 0 bp)	**111701232**	88494866	88685751	88477590	88259815	88449116	90604563	89660410
Total length (≥ 1000 bp)	81314165	82917065	83298061	82563788	**83582377**	83366588	83452630	82221160
Total length (≥ 5000 bp)	56548179	69077685	71125131	66852992	**75198734**	72219844	73292947	62595655
Total length (≥ 10000 bp)	32454389	50611835	54160580	46552182	**61611743**	55675021	57064546	40852265
Total length (≥ 25000 bp)	5145723	16921012	20768408	13067553	**30030862**	22904577	24067360	9419772
Total length (≥ 50000 bp)	174274	2388255	3302981	1588265	**7758002**	4107399	4809154	773905
# contigs	18340	11869	10998	13082	8702	10313	9907	15287
Largest contig	68123	85393	80374	97813	**102135**	94125	99626	81231
Total length	83591797	83953943	84236486	83787039	**84247240**	84198056	84227171	83825716
Reference length	107349540	107349540	107349540	107349540	107349540	107349540	107349540	107349540
GC (%)	40.66	40.72	40.71	40.68	40.73	40.72	40.73	40.48
Reference GC (%)	40.89	40.89	40.89	40.89	40.89	40.89	40.89	40.89
N50	7859	12805	14093	11414	**18281**	15129	15504	9663
NG50	5506	9159	10166	8182	**13099**	10719	11194	6836
N75	4000	6649	7403	5939	**9387**	7739	8062	4939
NG75	1167	1888	2116	1669	**2743**	2260	2380	1442
L50	3104	1927	1751	2173	**1355**	1633	1590	2514
LG50	4910	3012	2723	3395	**2103**	2542	2466	3958
L75	6828	4199	3815	4703	**2960**	3583	3463	5542
LG75	14473	8756	7888	9852	**6063**	7382	7039	11683
# misassemblies	119	820	640	716	353	496	**112**	689
# misassembled contigs	119	759	612	676	336	469	**110**	660
Misassembled contigs length	**985057**	10604473	9133094	8318230	6946313	7769701	2110750	6863900
# local misassemblies	44	41	49	54	40	42	**38**	44
# unaligned mis. contigs	0	0	0	0	0	0	0	0
# unaligned contigs	**13 + 6 part**	13 + 26 part	17 + 19 part	19 + 19 part	16 + 13 part	15 + 19 part	16 + 5 part	19 + 24 part
Unaligned length	16477	34851	30844	35033	26953	28976	**16274**	38607
Genome fraction (%)	77.424	77.785	78.122	77.703	**78.326**	78.150	78.312	77.521
Duplication ratio	1.006	1.005	1.004	1.004	**1.002**	1.003	**1.002**	1.007
# N's per 100 kbp	0.00	0.00	0.00	0.00	0.00	0.00	0.00	0.00
# mismatches per 100 kbp	112.22	126.99	119.91	125.01	105.67	114.90	**101.91**	118.25
# indels per 100 kbp	20.59	21.56	21.52	20.54	21.48	21.23	20.81	**17.42**
Largest alignment	68123	81510	80321	93310	**102135**	92098	99626	65458
Total aligned length	83425919	83704957	84047956	83625967	**84183013**	84039499	84171283	83422870
NA50	7792	11786	13177	10772	**17429**	14288	15290	9245
NGA50	5451	8475	9488	7737	**12409**	10103	11015	6440
NA75	3951	6058	6829	5537	**8948**	7315	7985	4624
NGA75	1121	1693	1929	1531	**2573**	2108	2345	1288
LA50	3124	2055	1841	2279	**1410**	1710	1610	2616
LGA50	4948	3230	2878	3569	**2192**	2675	2498	4141
LA75	6888	4522	4049	4968	**3090**	3776	3507	5822
LGA75	14685	9514	8458	10506	**6350**	7821	7138	12474

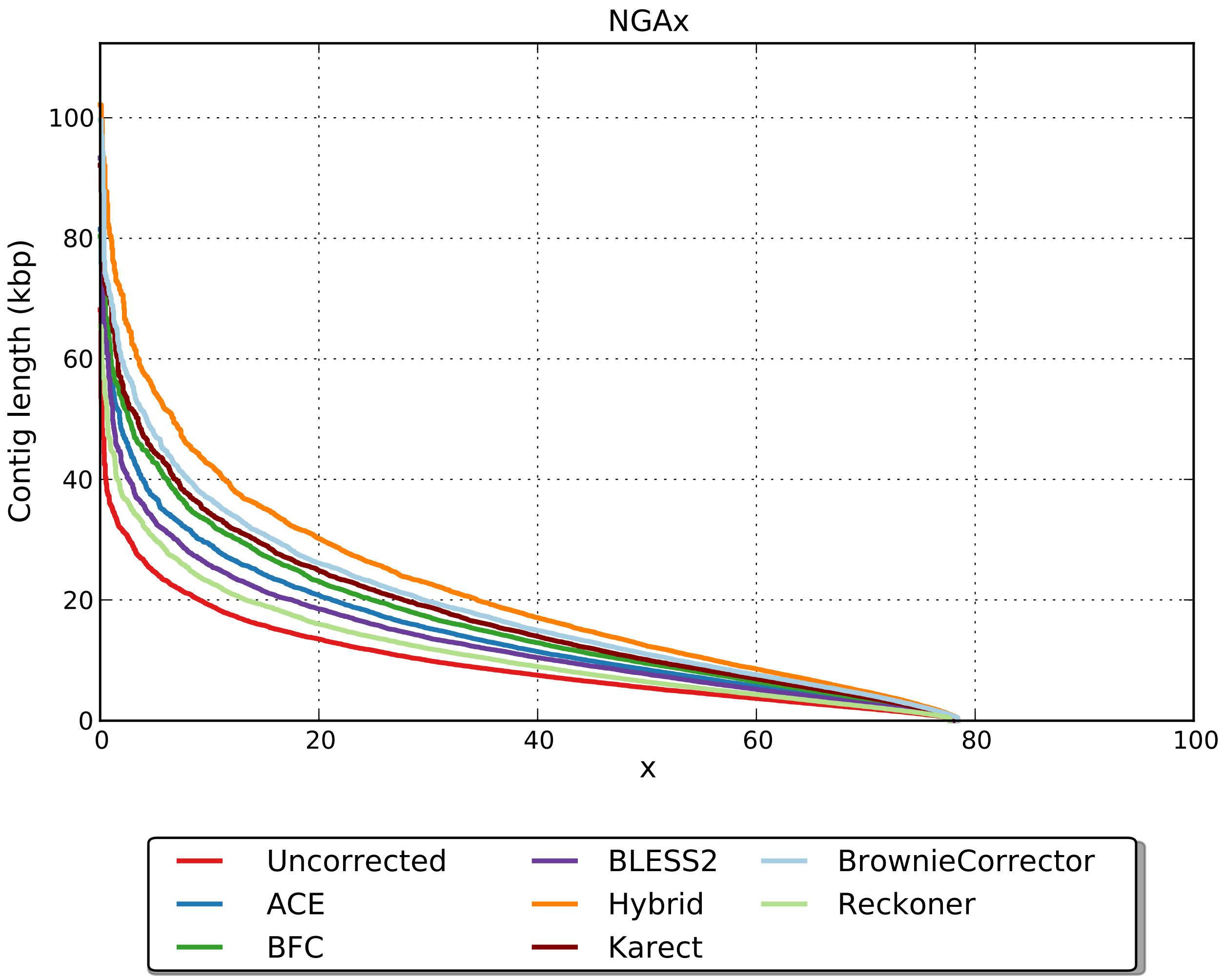

Figure 4: SPAdes assembly results for dataset D2 (*Homo sapiens* Chr. 14) for both uncorrected and corrected data. Contigs with length NGAx or larger produce x% of the genome.

5.4.3 D3

Table 9 contains the Quast report after assembling dataset D3 (*C. elegans*) with SPAdes. Fig. 5 shows the corresponding NGAx plot.

Table 9: Assembly quality metrics for D3

Assembly	Uncorrected	ACE	BFC	BLESS2	Hybrid	Karect	BrownieCorrector	Reckoner
# contigs (≥ 0 bp)	91054	108559	91618	117149	91976	91798	91152	93938
# contigs (≥ 1000 bp)	20133	26210	20179	28117	20190	20176	20128	20188
# contigs (≥ 5000 bp)	6202	5193	6215	4644	6253	6247	6201	6189
# contigs (≥ 10000 bp)	2336	1170	2330	909	2368	2364	2337	2333
# contigs (≥ 25000 bp)	280	120	296	70	300	302	280	286
# contigs (≥ 50000 bp)	65	22	65	7	56	56	65	63
Total length (≥ 0 bp)	116361844	112509995	116388634	112348944	116081137	116062821	116365458	**116425966**
Total length (≥ 1000 bp)	**103913389**	95599801	103773800	92664921	103437334	103440805	103912841	103655165
Total length (≥ 5000 bp)	70663972	46557489	70526082	38965131	70281028	70250754	**70666015**	70314693
Total length (≥ 10000 bp)	43594203	19178514	43288714	13815325	42981410	42929158	**43606770**	43257998
Total length (≥ 25000 bp)	13566746	5009175	**13613721**	2460232	12782149	12861875	13554231	13527463
Total length (≥ 50000 bp)	6657935	1656998	6061255	396865	4955692	4959945	**6657938**	6351558
# contigs	26593	36711	26767	40384	26788	26761	26589	26789
Largest contig	**244078**	128379	240394	66400	238991	238991	**244078**	240969
Total length	108572808	103180601	108528629	101465829	108202583	108197037	**108573218**	108420265
Reference length	100286070	100286070	100286070	100286070	100286070	100286070	100286070	100286070
GC (%)	38.47	38.30	38.46	38.20	38.41	38.41	38.47	38.46
Reference GC (%)	35.44	35.44	35.44	35.44	35.44	35.44	35.44	35.44
N50	7659	4466	7625	3834	7644	7654	**7663**	7620
NG50	8517	4608	8496	3887	8464	8474	**8522**	8483
N75	3546	2270	3525	1987	3516	3524	**3547**	3515
NG75	**4365**	2428	4325	2039	4295	4296	**4365**	4306
L50	3560	6259	3588	7341	3641	3641	**3559**	3588
LG50	3047	5940	3075	7189	3148	3149	**3046**	3082
L75	8761	14393	8811	16553	8852	8849	**8759**	8818
LG75	7178	13468	7224	16114	7320	7320	**7176**	7247
# misassemblies	1232	4500	1194	1484	**1190**	1220	1233	1219
# misassembled contigs	1178	4040	1142	1435	**1132**	1160	1180	1165
Misassembled contigs length	8966387	18042488	8729971	**5420189**	8771615	8913241	8958822	8888451
# local misassemblies	257	211	253	**202**	269	271	256	273
# unaligned mis. contigs	3	7	3	**2**	**2**	**2**	3	**2**
# unaligned contigs	4921 + 67 part	**4196 + 126 part**	4969 + 53 part	4590 + 57 part	5035 + 56 part	5035 + 57 part	4922 + 67 part	4955 + 64 part
Unaligned length	16560504	14110799	16496602	**13653429**	16286086	16288035	16560915	16494872
Genome fraction (%)	91.300	86.965	**91.318**	87.248	91.229	91.231	91.302	91.222
Duplication ratio	1.005	1.021	1.005	**1.004**	1.005	1.005	1.005	1.005
# N's per 100 kbp	0.00	0.00	0.00	0.00	0.00	0.00	0.00	0.00
# mismatches per 100 kbp	23.69	105.56	**23.11**	27.41	23.40	23.35	23.58	24.24
# indels per 100 kbp	5.88	18.20	**5.79**	6.64	5.90	5.94	5.86	5.94
Largest alignment	54032	27212	54032	27155	**64367**	**64367**	54032	54032
Total aligned length	91822498	87395766	**91852586**	87657662	91748458	91739544	91822652	91746223
NA50	5612	2996	5598	2925	**5613**	5609	5610	5561
NGA50	6325	3116	6307	2969	6297	6295	**6328**	6281
NA75	1916	1148	1915	1229	**1938**	**1938**	1918	1904
NGA75	**2713**	1300	2702	1282	2688	2688	**2713**	2674
LA50	5036	9332	5045	9570	**5018**	5019	5035	5066
LGA50	4341	8858	4351	9370	4352	4353	**4340**	4379
LA75	13065	22879	13084	22740	**12996**	**12996**	13062	13147
LGA75	10358	21103	10388	22035	10412	10415	**10356**	10463

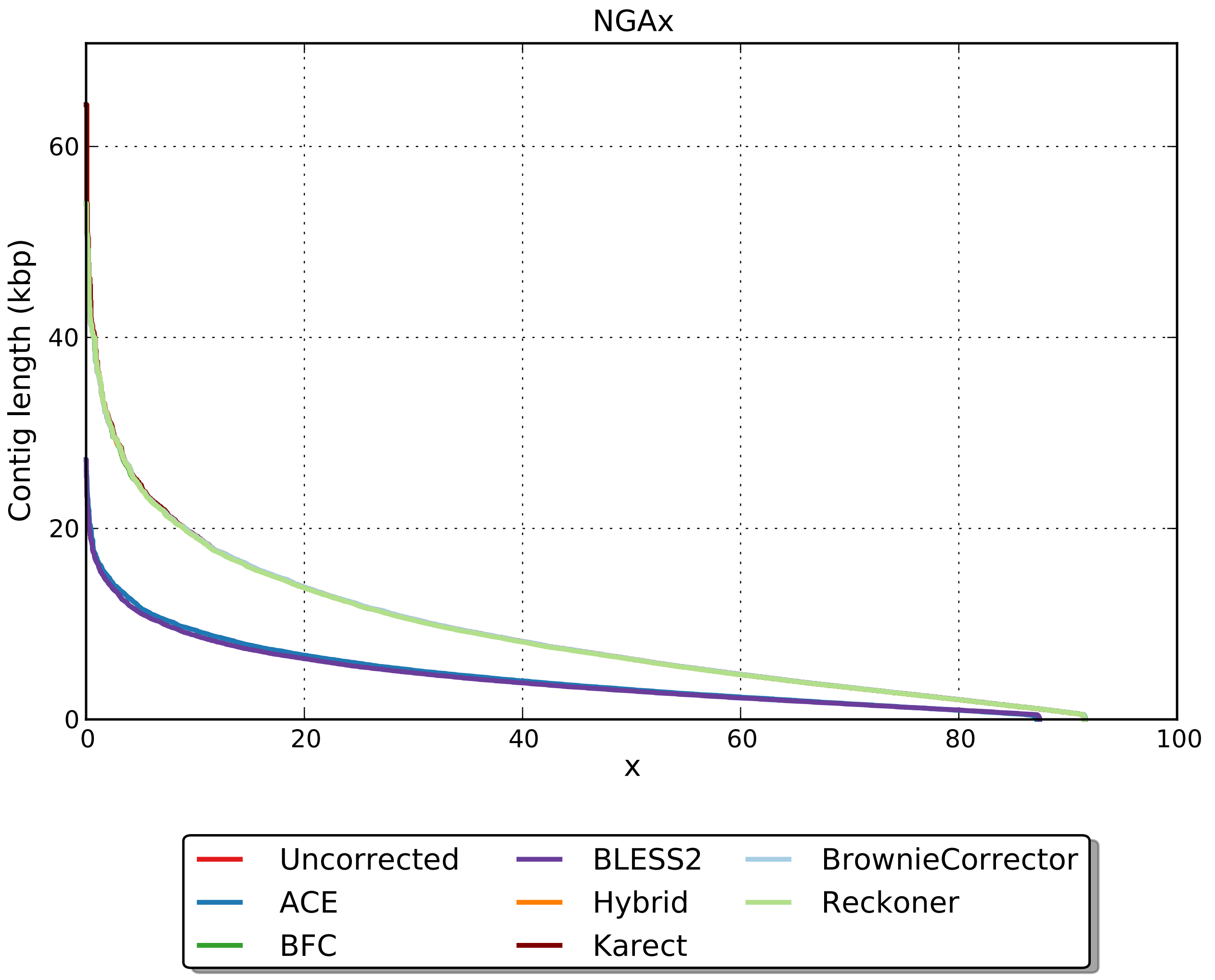

Figure 5: SPAdes assembly results for dataset D3 (*C. elegans*) for both uncorrected and corrected data. Contigs with length NGAx or larger produce x% of the genome.

5.4.4 D4

Table 10 contains the Quast report after assembling dataset D4 (*D. melanogaster*) with SPAdes. Fig. 6 shows the corresponding NGAx plot.

Table 10: Assembly quality metrics for D4

Assembly	Uncorrected	ACE	BFC	BLESS2	Hybrid	Karect	BrownieCorrector	Reckoner
# contigs (≥ 0 bp)	94428	98953	92673	98200	92770	93552	94263	102274
# contigs (≥ 1000 bp)	5744	7518	5816	8256	5478	5570	5641	6469
# contigs (≥ 5000 bp)	2927	4348	3019	4686	2685	2771	2859	3415
# contigs (≥ 10000 bp)	2228	3088	2286	3275	2064	2129	2166	2517
# contigs (≥ 25000 bp)	1289	1412	1321	1366	1250	1280	1272	1383
# contigs (≥ 50000 bp)	687	524	674	455	687	695	690	656
Total length (≥ 0 bp)	130002600	128372226	129827421	127011744	129810748	129881140	129985751	**130506403**
Total length (≥ 1000 bp)	119818544	118251920	119812405	116972812	119873419	**119877304**	119808428	119794237
Total length (≥ 5000 bp)	113581978	110643148	113604067	108274776	**113773726**	113762300	113688120	112852066
Total length (≥ 10000 bp)	108530954	101505638	108274117	98172191	**109260361**	109119112	108675878	106389897
Total length (≥ 25000 bp)	93186659	74193597	92453290	67343513	**95998464**	95254278	94026163	88095244
Total length (≥ 50000 bp)	71303295	42715781	69206434	35471350	**75528880**	73949504	72922308	62048435
# contigs	8394	9886	8486	10424	8037	8145	8281	9166
Largest contig	481990	333642	481989	287346	517988	**517989**	481989	454387
Total length	121670559	119897165	121671950	118480841	121661557	**121679265**	121652919	121676103
Reference length	120381546	120381546	120381546	120381546	120381546	120381546	120381546	120381546
GC (%)	42.57	42.53	42.57	42.52	42.58	42.58	42.57	42.55
Reference GC (%)	42.42	42.42	42.42	42.42	42.42	42.42	42.42	42.42
N50	65546	35232	61819	30326	**75991**	71129	69567	51134
NG50	66349	35003	62540	29666	**76873**	72000	70449	52030
N75	27402	16276	26503	14275	**31163**	29995	28393	21974
NG75	28467	16087	27640	13542	**32518**	31195	29583	22982
L50	505	935	524	1071	**446**	473	485	633
LG50	496	942	514	1103	**438**	464	476	620
L75	1216	2191	1275	2496	**1082**	1136	1169	1518
LG75	1181	2214	1239	2599	**1052**	1104	1136	1475
# misassemblies	814	964	829	997	804	**799**	805	879
# misassembled contigs	635	820	650	870	**623**	628	629	705
Misassembled contigs length	43763416	31185704	43389755	**29155670**	47975869	45770362	44692094	41789688
# local misassemblies	1310	**1248**	1307	1268	1292	1298	1300	1321
# unaligned mis. contigs	28	**19**	27	22	31	31	28	21
# unaligned contigs	3858 + 378 part	2942 + 430 part	3844 + 384 part	**2440 + 373 part**	3824 + 384 part	3840 + 373 part	3852 + 378 part	3853 + 373 part
Unaligned length	8132812	6534991	8141896	**5455940**	8172885	8173915	8132689	8117761
Genome fraction (%)	93.913	93.622	93.907	93.481	93.915	**93.916**	93.910	93.890
Duplication ratio	**1.004**	1.006	**1.004**	**1.004**	**1.004**	**1.004**	**1.004**	1.005
# N's per 100 kbp	0.00	0.00	0.00	0.00	0.00	0.00	0.00	0.00
# mismatches per 100 kbp	551.02	561.24	551.22	560.94	**548.74**	549.34	548.84	550.02
# indels per 100 kbp	131.61	133.42	131.69	132.59	131.78	131.70	131.41	**130.95**
Largest alignment	406513	188221	280215	167357	**428481**	**428481**	406512	367383
Total aligned length	**113196867**	112830607	113186685	112666190	113178942	113185629	113187949	113182400
NA50	50135	29320	48237	25498	**55055**	53564	51322	41401
NGA50	50833	29126	49089	25133	**56046**	54106	52152	41977
NA75	21271	13471	20789	12059	**23983**	23202	22189	17553
NGA75	22149	13335	21467	11383	**24872**	23983	22983	18369
LA50	656	1110	680	1263	**600**	623	639	800
LGA50	643	1119	667	1300	**588**	611	627	785
LA75	1573	2616	1635	2929	**1429**	1479	1527	1913
LGA75	1529	2643	1589	3050	**1390**	1437	1484	1859

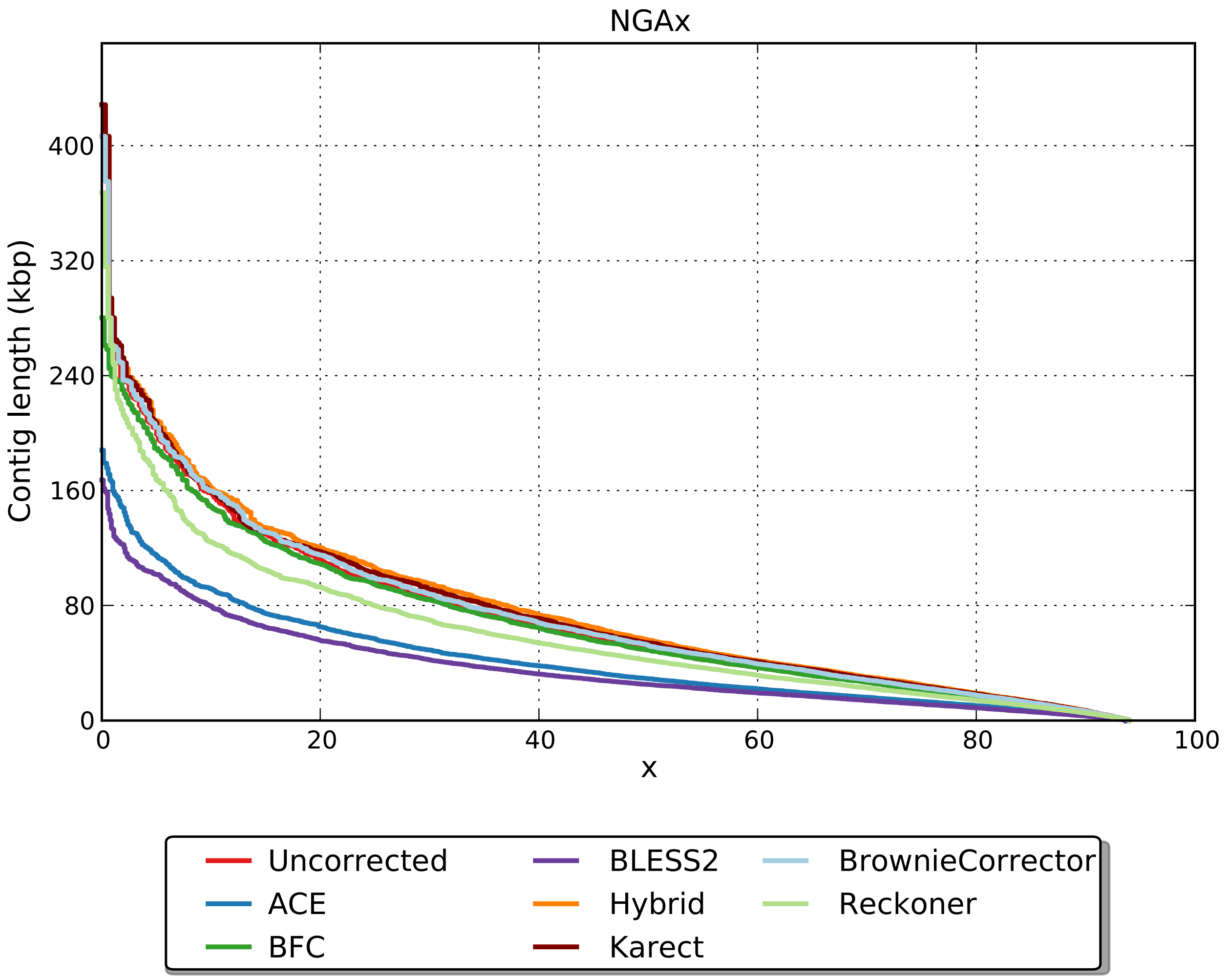

Figure 6: SPAdes assembly results for dataset D4 (*D. melanogaster*) for both uncorrected and corrected data. Contigs with length NGAx or larger produce x% of the genome.

5.4.5 D5

Table 11 contains the Quast report after assembling dataset D5 (*D. melanogaster*) with SPAdes. Fig. 7 shows the corresponding NGAx plot.

Table 11: Assembly quality metrics for D5

Assembly	Uncorrected	ACE	BFC	BLESS2	Hybrid	Karect	BrownieCorrector	Reckoner
# contigs (≥ 0 bp)	170491	218463	192792	204579	188520	189526	170354	224879
# contigs (≥ 1000 bp)	7103	10411	8931	11456	8433	8613	6826	9131
# contigs (≥ 5000 bp)	3782	5247	4591	5573	4294	4399	3605	4692
# contigs (≥ 10000 bp)	2659	3014	2901	3072	2741	2794	2557	2928
# contigs (≥ 25000 bp)	1336	1059	1226	958	1196	1203	1329	1217
# contigs (≥ 50000 bp)	575	353	469	321	505	502	595	462
Total length (≥ 0 bp)	136008271	136884556	137701577	135338694	137359979	137407270	136022594	**139938723**
Total length (≥ 1000 bp)	118770197	115965322	118413811	115640342	118358084	118349944	**118780111**	118408852
Total length (≥ 5000 bp)	110806731	102620615	107643770	100398087	108148059	107936808	**111106120**	107416341
Total length (≥ 10000 bp)	102774604	86665062	95540225	82570772	97010422	96455953	**103631979**	94709945
Total length (≥ 25000 bp)	81170209	55974685	68911764	49661452	72438499	71191324	**83690560**	67424844
Total length (≥ 50000 bp)	54276219	31966506	42454776	27765715	48170727	46747732	**57723562**	41149200
# contigs	9227	12936	11267	13893	10631	10821	8939	11501
Largest contig	535439	**579123**	479301	348825	579114	459363	579114	330198
Total length	120260659	117783200	120069086	117402937	119916284	119916237	**120263583**	120090128
Reference length	120381546	120381546	120381546	120381546	120381546	120381546	120381546	120381546
GC (%)	42.42	42.52	42.43	42.53	42.44	42.43	42.42	42.42
Reference GC (%)	42.42	42.42	42.42	42.42	42.42	42.42	42.42	42.42
N50	43681	23183	31663	19964	36541	34845	**47564**	30523
NG50	43568	22418	31484	19004	36446	34551	**47534**	30359
N75	18624	9438	12639	8321	13701	13265	**19903**	12158
NG75	18613	8787	12489	7717	13517	13027	**19868**	12052
L50	700	1181	910	1364	782	820	**645**	950
LG50	702	1238	915	1441	788	827	**646**	955
L75	1753	3186	2414	3675	2135	2226	**1620**	2509
LG75	1758	3400	2433	3953	2161	2253	**1624**	2527
# misassemblies	751	802	744	757	**733**	735	742	768
# misassembled contigs	624	715	633	687	615	630	**610**	664
Misassembled contigs length	34931702	25507758	28643451	**21355751**	31273118	30251303	37027268	28868487
# local misassemblies	1112	**1011**	1066	1012	1071	1069	1119	1071
# unaligned mis. contigs	27	30	28	**18**	28	31	26	31
# unaligned contigs	3151 + 365 part	2070 + 380 part	3080 + 369 part	**1892 + 344 part**	2967 + 347 part	2973 + 349 part	3150 + 362 part	3099 + 361 part
Unaligned length	6896001	4890803	6910766	**4596029**	6739536	6742165	6889509	6879138
Genome fraction (%)	93.781	93.282	93.578	93.194	93.585	93.595	**93.789**	93.582
Duplication ratio	**1.004**	1.005	1.005	1.006	1.005	**1.004**	**1.004**	1.005
# N's per 100 kbp	0.00	0.00	0.00	0.00	0.00	0.00	0.00	0.00
# mismatches per 100 kbp	501.29	499.27	498.06	501.93	**495.52**	496.83	498.78	498.23
# indels per 100 kbp	119.51	117.33	118.14	**117.24**	118.01	118.21	119.22	118.08
Largest alignment	402868	313000	334917	333479	334919	334338	**402877**	314918
Total aligned length	113092386	112548843	112881644	112507933	112929042	112914657	**113119011**	112930607
NA50	35938	20690	27488	17825	30759	29547	**38675**	26330
NGA50	35924	20032	27365	17133	30557	29286	**38670**	26296
NA75	15941	8353	11070	7535	11943	11648	**16869**	10658
NGA75	15889	7768	10966	6898	11792	11491	**16840**	10579
LA50	865	1373	1086	1556	956	990	**813**	1128
LGA50	866	1436	1092	1641	964	998	**815**	1134
LA75	2115	3624	2802	4096	2516	2602	**1979**	2917
LGA75	2120	3866	2823	4406	2546	2633	**1984**	2938

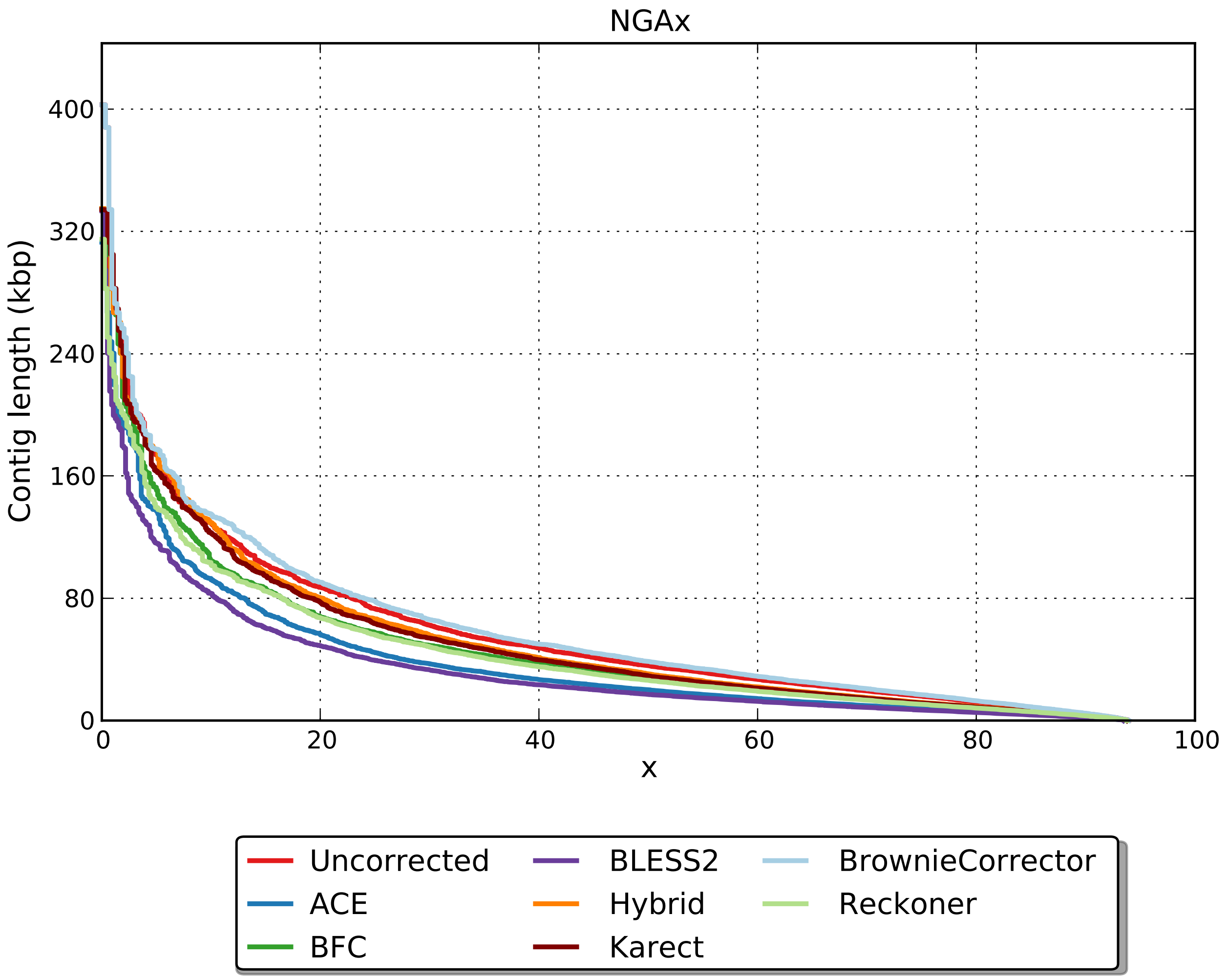

Figure 7: SPAdes assembly results for dataset D5 (*D. melanogaster*) for both uncorrected and corrected data. Contigs with length NGAx or larger produce x% of the genome.

5.4.6 D6

Table 12 contains the Quast report after assembling dataset D6 (*A. thaliana*) with SPAdes. Fig. 8 shows the corresponding NGAx plot.

Table 12: Assembly quality metrics for D6

Assembly	Uncorrected	ACE	BFC	BLESS2	Hybrid	Karect	BrownieCorrector	Reckoner
# contigs (≥ 0 bp)	153426	169680	152423	159228	156417	157208	153325	169100
# contigs (≥ 1000 bp)	5274	5881	5208	6509	4992	5200	5007	5377
# contigs (≥ 5000 bp)	3398	3767	3381	4038	3196	3344	3196	3452
# contigs (≥ 10000 bp)	2530	2730	2516	2841	2400	2501	2389	2565
# contigs (≥ 25000 bp)	1330	1302	1311	1313	1288	1307	1286	1307
# contigs (≥ 50000 bp)	589	523	588	484	603	584	612	591
Total length (≥ 0 bp)	122790524	123570159	122779397	122265471	123023997	123085625	122779928	**123950644**
Total length (≥ 1000 bp)	108694789	108396090	**108822375**	107698581	108735045	108743759	108694535	108777289
Total length (≥ 5000 bp)	104074942	103085055	**104321365**	101443382	104314160	104125163	104260540	103974120
Total length (≥ 10000 bp)	97777795	95556603	98031267	92759620	**98505752**	97989568	98403336	97527917
Total length (≥ 25000 bp)	78164544	72474598	78262483	68171653	80366270	78482167	**80445350**	76741954
Total length (≥ 50000 bp)	51919360	44842751	52621466	39368697	55944501	52808127	**56505536**	51275596
# contigs	6731	7363	6608	8051	6416	6647	6436	6809
Largest contig	378346	360514	337032	263399	**403164**	358676	360062	292751
Total length	109681747	109400465	**109771235**	108742773	109698307	109722386	109662957	109743868
Reference length	119668634	119668634	119668634	119668634	119668634	119668634	119668634	119668634
GC (%)	35.96	35.98	35.96	36.00	35.97	35.96	35.96	35.96
Reference GC (%)	36.06	36.06	36.06	36.06	36.06	36.06	36.06	36.06
N50	46858	40291	47899	34704	51194	48131	**52341**	46233
NG50	41749	35499	42189	30758	45986	42290	**46094**	40862
N75	21989	18318	22072	16335	23637	22196	**23725**	21324
NG75	15914	13442	16240	11682	**16977**	16014	16925	15642
L50	650	743	635	844	582	626	**580**	666
LG50	762	878	745	1012	684	736	**682**	780
L75	1504	1746	1484	1972	1367	1468	**1360**	1547
LG75	1903	2236	1873	2565	1737	1859	**1733**	1953
# misassemblies	149	138	157	174	**112**	129	118	137
# misassembled contigs	148	136	154	168	**111**	123	117	132
Misassembled contigs length	6392285	5198138	8078221	4928846	**4684101**	5431837	5236199	6091039
# local misassemblies	50	**43**	44	69	50	52	46	46
# unaligned mis. contigs	2	2	2	**1**	**1**	2	2	2
# unaligned contigs	238 + 21 part	159 + 23 part	223 + 24 part	**93 + 30 part**	231 + 27 part	235 + 27 part	237 + 22 part	232 + 25 part
Unaligned length	406289	241175	405558	**184809**	394256	396474	404121	406267
Genome fraction (%)	91.207	91.079	**91.283**	90.628	91.253	91.255	91.215	91.255
Duplication ratio	**1.001**	1.002	**1.001**	**1.001**	**1.001**	**1.001**	**1.001**	**1.001**
# N's per 100 kbp	0.00	0.00	0.00	0.00	0.00	0.00	0.00	0.00
# mismatches per 100 kbp	20.01	25.24	19.63	22.12	18.53	19.60	**18.22**	18.97
# indels per 100 kbp	5.00	7.27	4.94	6.27	4.81	4.83	**4.75**	4.85
Largest alignment	378346	360476	322214	263398	**402910**	358628	360023	292696
Total aligned length	109234355	109062189	**109324450**	108528876	109272392	109287962	109225834	109293988
NA50	45861	39070	45958	33942	50386	46858	**51386**	45215
NGA50	40802	34273	40910	29968	**45423**	41391	45400	39605
NA75	21247	17857	21475	15900	**23153**	21665	22895	20809
NGA75	15318	13110	15574	11412	**16504**	15526	16405	15190
LA50	665	760	658	862	**591**	638	**591**	683
LGA50	781	900	772	1033	695	751	**694**	801
LA75	1544	1793	1537	2019	**1388**	1501	**1388**	1590
LGA75	1958	2296	1942	2628	**1769**	1906	1773	2007

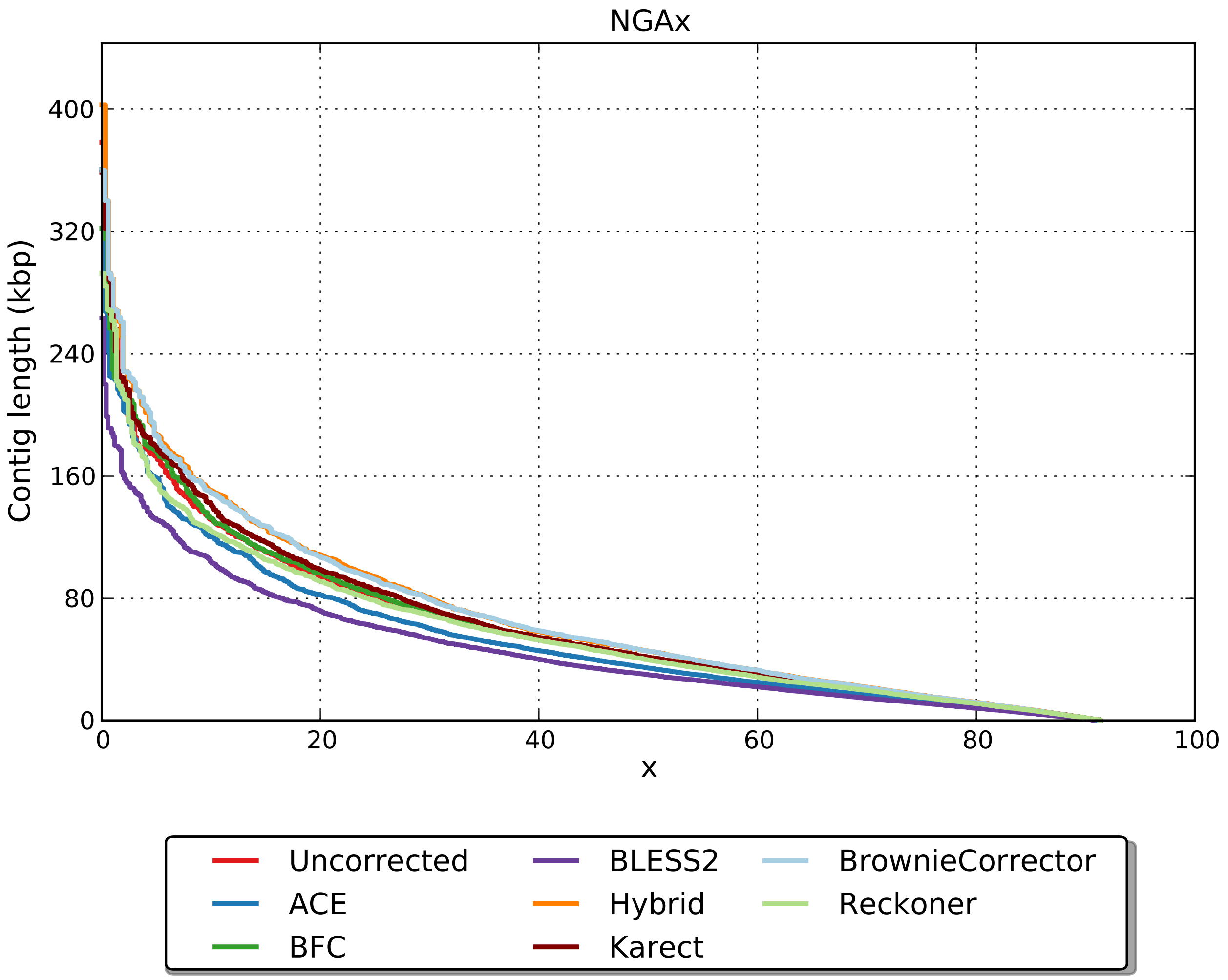

Figure 8: SPAdes assembly results for dataset D6 (*A. thaliana*) for both uncorrected and corrected data. Contigs with length NGAx or larger produce x% of the genome.

5.4.7 D7

Table 13 contains the Quast report after a hybrid assembly of dataset D7 (*D. melanogaster*) with SPAdes. This is a hybrid assembly in which the corrected (and uncorrected) Illumina reads (R4) are complemented with the Pacbio reads (P1). Fig. 9 shows the corresponding NGAx plot.

Table 13: Assembly quality metrics for D7

Assembly	Uncorrected	ACE	BFC	BLESS2	Hybrid	Karect	BrownieCorrector	Reckoner
# contigs (≥ 0 bp)	84104	87785	82976	85783	82832	83398	84087	92296
# contigs (≥ 1000 bp)	4269	4797	4408	4424	4195	4220	4221	5118
# contigs (≥ 5000 bp)	2128	2801	2230	2527	2012	2071	2099	2743
# contigs (≥ 10000 bp)	1600	2088	1665	1913	1486	1531	1566	2031
# contigs (≥ 25000 bp)	1013	1231	1045	1161	967	985	1004	1218
# contigs (≥ 50000 bp)	653	701	666	691	633	649	644	705
Total length (≥ 0 bp)	130506649	128784476	130252545	127655011	130240850	130280733	130480461	**130996905**
Total length (≥ 1000 bp)	**121778040**	120151535	121628181	119271937	121725554	121710656	121748117	121706993
Total length (≥ 5000 bp)	116980449	115355940	116748510	114676443	116909862	116920459	**117020950**	116233150
Total length (≥ 10000 bp)	113202715	110296487	112661638	110309610	113145023	113052834	**113234024**	111121525
Total length (≥ 25000 bp)	103664419	96387206	102536172	98158594	**104756954**	104285446	104149759	97981828
Total length (≥ 50000 bp)	90541399	77320552	88785758	81304930	**92631370**	92090502	91215132	79688544
# contigs	6250	6413	6427	5633	6104	6140	6209	7163
Largest contig	754587	545648	667406	602677	829779	829776	855558	**887190**
Total length	**123171040**	121267612	123042637	120101070	123066199	123060468	123143110	123138443
Reference length	120381546	120381546	120381546	120381546	120381546	120381546	120381546	120381546
GC (%)	42.55	42.49	42.54	42.46	42.55	42.55	42.54	42.52
Reference GC (%)	42.42	42.42	42.42	42.42	42.42	42.42	42.42	42.42
N50	120821	74655	113294	89691	**130622**	126171	124369	79327
NG50	123823	75089	114878	89541	**135170**	129017	128600	81109
N75	47031	32264	44284	37050	**50374**	49513	47556	32089
NG75	50427	33004	48111	36516	**54468**	53085	51344	34919
L50	284	431	303	369	**261**	274	276	413
LG50	273	425	291	371	**250**	263	265	396
L75	692	1041	740	896	**627**	654	668	1019
LG75	648	1021	697	902	**588**	614	626	957
# misassemblies	1048	1132	1050	1206	1014	**1001**	1029	1143
# misassembled contigs	668	809	665	791	**640**	642	661	769
Misassembled contigs length	66619419	57679056	64061457	60021320	68314711	66950147	66893425	**57223037**
# local misassemblies	1572	**1480**	1515	1548	1506	1536	1562	1655
# unaligned mis. contigs	135	127	127	**96**	118	123	120	122
# unaligned contigs	3288 + 593 part	2361 + 651 part	3294 + 629 part	**1838 + 541 part**	3296 + 640 part	3267 + 637 part	3286 + 598 part	3311 + 622 part
Unaligned length	8782559	7074733	8683828	**6035908**	8759389	8758466	8761387	8732208
Genome fraction (%)	**94.496**	94.284	94.491	94.241	94.491	94.468	94.494	94.444
Duplication ratio	1.006	1.006	**1.005**	**1.005**	**1.005**	**1.005**	1.006	1.006
# N's per 100 kbp	0.00	0.00	0.00	0.00	0.00	0.00	0.00	0.00
# mismatches per 100 kbp	558.01	567.44	558.79	565.53	556.90	**556.23**	556.57	558.35
# indels per 100 kbp	134.40	140.90	134.58	145.25	134.03	134.02	134.08	**133.68**
Largest alignment	486030	417035	428461	338930	**547379**	547363	447086	406192
Total aligned length	**114077574**	113784885	114050875	113749971	114018850	114005038	114071871	114037057
NA50	79151	54862	74311	61784	**86507**	82808	80392	56396
NGA50	80752	55391	77526	61609	**89065**	85226	83397	58176
NA75	32513	23228	30391	25843	**35224**	34107	33310	23753
NGA75	35171	23744	33021	25562	**37912**	36680	35880	25771
LA50	429	602	453	545	**406**	416	422	585
LGA50	412	593	435	547	**391**	400	405	561
LA75	1042	1443	1097	1291	**971**	997	1020	1417
LGA75	980	1415	1034	1299	**915**	940	961	1334

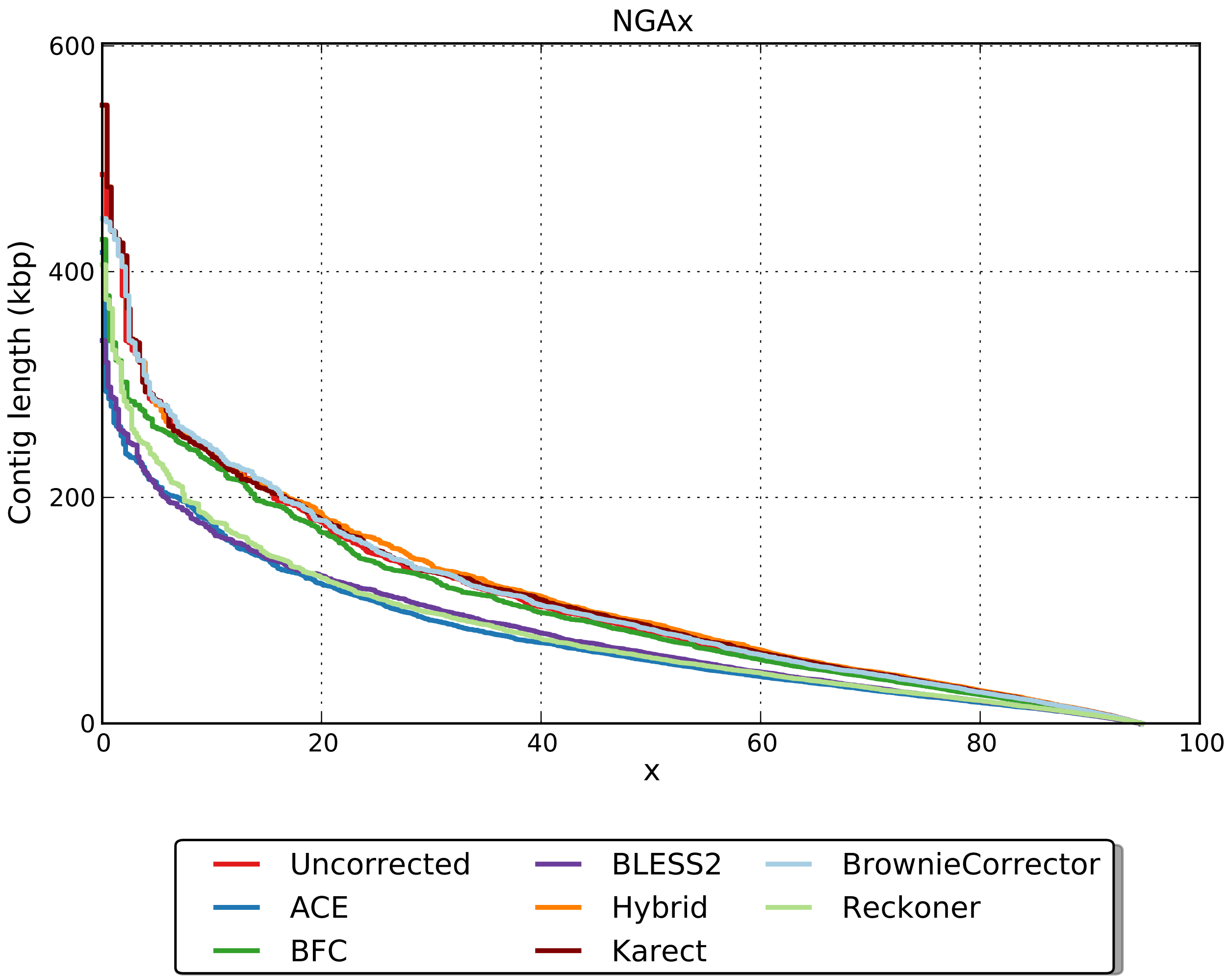

Figure 9: SPAdes assembly results for dataset D7 (*D. melanogaster*) for both uncorrected and corrected data. Contigs with length NGAx or larger produce x% of the genome.

5.4.8 D8

Table 14 contains the Quast report after assembling dataset D8 *D. melanogaster* with SPAdes. This is a hybrid assembly in which the corrected (and uncorrected) Illumina reads (R5) are complemented with the Pacbio reads (P1). Fig. 10 shows the corresponding NGAx plot.

Table 14: Assembly quality metrics for D8

Assembly	Uncorrected	ACE	BFC	BLESS2	Hybrid	Karect	BrownieCorrector	Reckoner
# contigs (≥ 0 bp)	153257	193843	171323	177917	167024	168273	153096	203849
# contigs (≥ 1000 bp)	4205	4162	4361	4367	4019	4123	3990	4569
# contigs (≥ 5000 bp)	2010	2450	2147	2596	1903	1960	1853	2326
# contigs (≥ 10000 bp)	1473	1845	1572	1909	1402	1450	1363	1701
# contigs (≥ 25000 bp)	1003	1153	1044	1206	975	992	944	1107
# contigs (≥ 50000 bp)	666	675	677	712	676	672	641	690
Total length (≥ 0 bp)	135887496	136343035	137423351	134765843	136961075	137049756	135884144	**139590516**
Total length (≥ 1000 bp)	120696663	118502898	120701123	118317880	120560238	120537455	**120706033**	120624736
Total length (≥ 5000 bp)	115654547	114306741	115613997	113904254	115684153	115538672	**115826462**	115470305
Total length (≥ 10000 bp)	111879129	109943429	111550471	109013641	112117292	111917113	**112393212**	111018421
Total length (≥ 25000 bp)	104315319	98751430	102975275	97606444	105279669	104552044	**105746772**	101450513
Total length (≥ 50000 bp)	92213430	81384118	89809178	79738854	94488203	92912592	**94775486**	86538407
# contigs	5706	5518	5821	5517	5350	5482	5466	6036
Largest contig	740414	714940	690671	676184	929948	829272	**949798**	715670
Total length	**121725596**	119424679	121699360	119105224	121471450	121469690	121719241	121630853
Reference length	120381546	120381546	120381546	120381546	120381546	120381546	120381546	120381546
GC (%)	42.38	42.47	42.38	42.47	42.39	42.39	42.38	42.38
Reference GC (%)	42.42	42.42	42.42	42.42	42.42	42.42	42.42	42.42
N50	125354	92982	112004	82650	127431	123975	**137513**	101811
NG50	126353	92254	113406	81120	129321	125108	**139117**	103109
N75	51706	38805	47534	36572	**57471**	54567	57390	41325
NG75	53948	37895	49131	35352	**59245**	55512	58973	42751
L50	270	355	297	397	261	271	**239**	333
LG50	265	360	291	405	257	267	**234**	327
L75	648	861	708	936	614	638	**577**	794
LG75	629	880	687	963	600	623	**559**	772
# misassemblies	892	919	876	**860**	870	864	894	936
# misassembled contigs	581	648	576	656	562	577	**561**	634
Misassembled contigs length	64530411	54903095	59072274	**50276098**	63420552	63011931	68069287	57826313
# local misassemblies	1487	1426	1526	1432	1451	**1422**	1493	1482
# unaligned mis. contigs	93	90	112	99	99	104	**89**	97
# unaligned contigs	2813 + 500 part	1718 + 495 part	2666 + 539 part	**1512 + 466 part**	2558 + 510 part	2563 + 512 part	2802 + 506 part	2694 + 510 part
Unaligned length	7569584	5423140	7538412	**5168064**	7336591	7325032	7588629	7445130
Genome fraction (%)	**94.400**	94.223	94.357	94.163	94.379	94.392	94.394	94.358
Duplication ratio	1.005	1.005	1.005	1.005	1.005	1.005	**1.004**	1.005
# N's per 100 kbp	0.00	0.00	0.00	0.00	0.00	0.00	0.00	0.00
# mismatches per 100 kbp	505.96	503.80	501.44	505.43	501.69	**501.23**	504.97	502.37
# indels per 100 kbp	120.43	119.21	**118.69**	121.82	118.72	118.84	120.22	118.87
Largest alignment	453436	453475	440801	402865	517576	**587714**	508703	453456
Total aligned length	113902949	113689117	113904771	113665384	113890437	113897046	113888974	**113906686**
NA50	83845	65926	78250	61557	86556	85122	**87799**	70604
NGA50	85003	65163	78985	60574	87822	85881	**88877**	71724
NA75	35457	29274	33797	28409	38951	37932	**39505**	30157
NGA75	36760	28366	34986	27330	40266	38829	**41434**	31616
LA50	419	507	433	529	399	403	**391**	470
LGA50	411	515	424	539	393	397	**384**	461
LA75	972	1183	1021	1234	911	936	**896**	1119
LGA75	944	1208	993	1268	890	915	**871**	1089

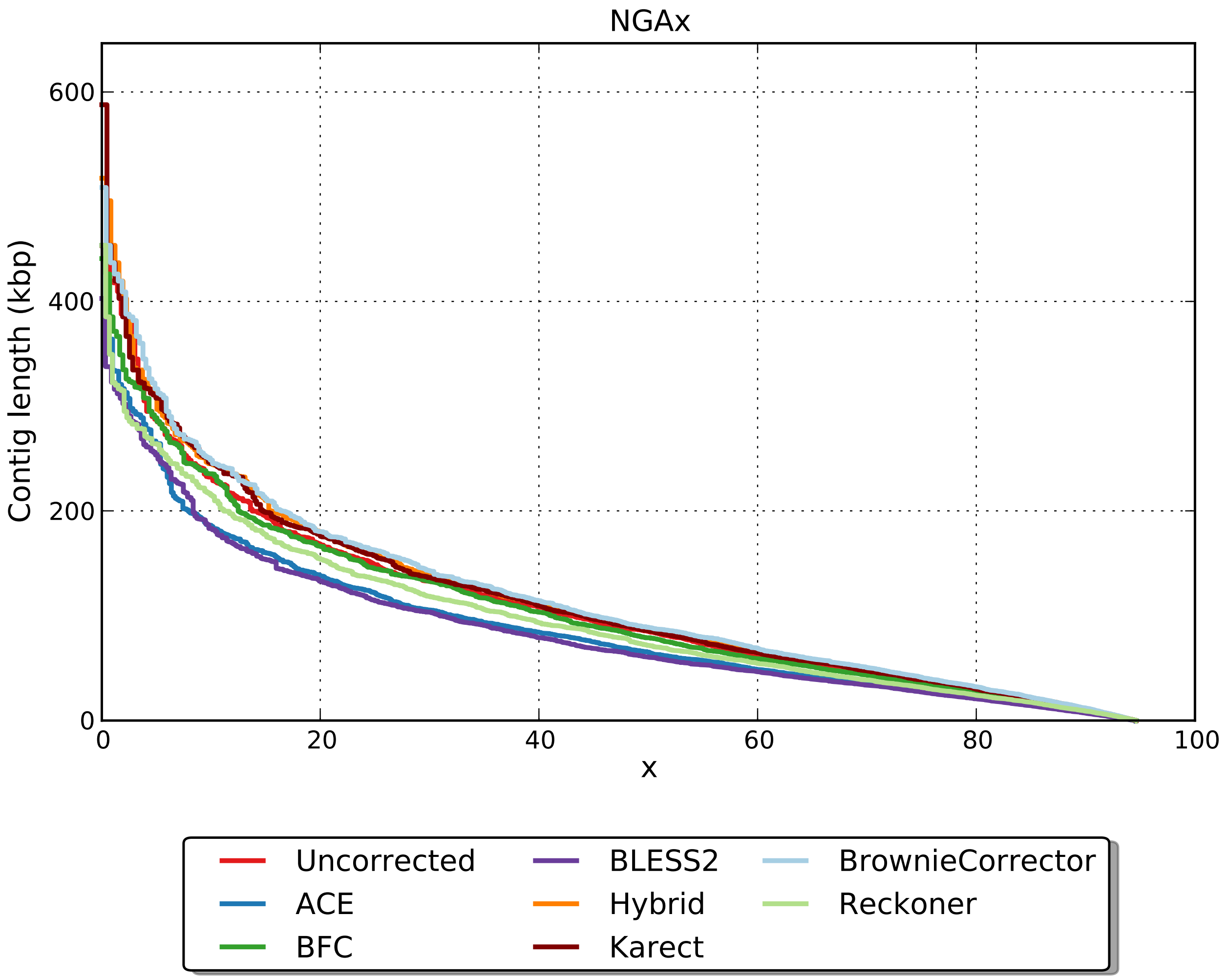

Figure 10: SPAdes assembly results for dataset D8 (*D. melanogaster*) for both uncorrected and corrected data. Contigs with length NGAx or larger produce x% of the genome.

5.4.9 D9

Table 15 contains the Quast report after assembling dataset D9 (*A. thaliana*) with SPAdes. This is a hybrid assembly in which the corrected (and uncorrected) Illumina reads (R6) are complemented with the Pacbio reads (P2). Fig. 11 shows the corresponding NGAx plot.

Table 15: Assembly quality metrics for D9

Assembly	Uncorrected	ACE	BFC	BLESS2	Hybrid	Karect	BrownieCorrector	Reckoner
# contigs (≥ 0 bp)	142998	158391	142712	148192	146017	147021	142974	158545
# contigs (≥ 1000 bp)	4033	4220	3993	4545	3926	4013	3896	4284
# contigs (≥ 5000 bp)	2412	2542	2430	2705	2300	2377	2292	2631
# contigs (≥ 10000 bp)	1802	1898	1808	1985	1717	1777	1711	1982
# contigs (≥ 25000 bp)	1109	1099	1113	1140	1048	1081	1062	1198
# contigs (≥ 50000 bp)	638	636	633	618	621	631	620	660
Total length (≥ 0 bp)	123218064	123981967	123224625	122885449	123518075	123539458	123245160	**124331702**
Total length (≥ 1000 bp)	110256731	109999806	110353964	109476185	**110361604**	110330888	110301171	110311214
Total length (≥ 5000 bp)	106152731	105686683	**106414045**	104796912	106244053	106169772	106242186	106088913
Total length (≥ 10000 bp)	101804805	101043252	101979115	99699235	102079432	101873514	**102085247**	101431478
Total length (≥ 25000 bp)	90414664	87963335	90363257	85957292	91028806	90379967	**91415021**	88457127
Total length (≥ 50000 bp)	73498519	71549764	73040202	67808341	**75725947**	74247235	75503677	69145241
# contigs	5138	5334	5059	5669	4997	5078	4983	5346
Largest contig	513726	548881	636585	608766	638980	604382	**638981**	568204
Total length	111015037	110759642	111083449	110246718	**111096537**	111062930	111043967	111041653
Reference length	119668634	119668634	119668634	119668634	119668634	119668634	119668634	119668634
GC (%)	35.94	35.96	35.95	35.96	35.95	35.95	35.94	35.94
Reference GC (%)	36.06	36.06	36.06	36.06	36.06	36.06	36.06	36.06
N50	82512	78856	83316	71534	**94701**	86750	93967	71963
NG50	75466	70566	74799	61723	**84439**	78085	82118	64130
N75	35434	31830	34731	28802	37344	35373	**37856**	31577
NG75	25874	23118	25874	20720	26758	25818	**27539**	23731
L50	359	380	363	403	325	345	**324**	433
LG50	414	439	417	474	**373**	397	374	496
L75	870	926	877	1018	**796**	845	800	1015
LG75	1083	1174	1089	1308	**999**	1057	**999**	1252
# misassemblies	617	597	596	617	574	**570**	572	603
# misassembled contigs	463	469	454	482	437	438	**428**	452
Misassembled contigs length	27054338	25914859	27793634	**22441602**	26444291	25929567	26374921	23718330
# local misassemblies	457	**432**	453	520	445	437	459	465
# unaligned mis. contigs	9	**7**	**7**	**7**	9	8	**7**	**7**
# unaligned contigs	270 + 142 part	190 + 127 part	254 + 126 part	**142 + 142 part**	257 + 130 part	271 + 121 part	264 + 135 part	265 + 122 part
Unaligned length	677416	**488901**	635261	494795	679750	651220	685268	634358
Genome fraction (%)	91.801	91.738	**91.908**	91.321	91.861	91.856	91.817	91.843
Duplication ratio	**1.004**	**1.004**	**1.004**	**1.004**	**1.004**	**1.004**	**1.004**	1.005
# N's per 100 kbp	0.00	0.00	0.00	0.00	0.00	0.00	0.00	0.00
# mismatches per 100 kbp	51.77	58.12	52.17	58.54	51.78	52.60	51.13	**50.56**
# indels per 100 kbp	10.92	15.70	10.85	21.11	10.38	10.59	10.35	**10.34**
Largest alignment	513722	527818	540557	608680	635802	604382	**635803**	567176
Total aligned length	110257880	110165936	**110362885**	109666875	110338430	110333035	110274459	110310731
NA50	74168	70306	72208	64696	**84946**	77951	82261	62281
NGA50	65138	62161	64709	55639	**74620**	68873	71788	56734
NA75	30088	27435	30288	25407	32098	31063	**32833**	27426
NGA75	22319	20022	22827	17956	23545	22847	**23679**	21034
LA50	398	418	409	440	**359**	384	361	479
LGA50	461	485	472	519	**413**	443	417	552
LA75	984	1047	1003	1137	**900**	958	906	1141
LGA75	1235	1331	1251	1469	**1136**	1202	1140	1410

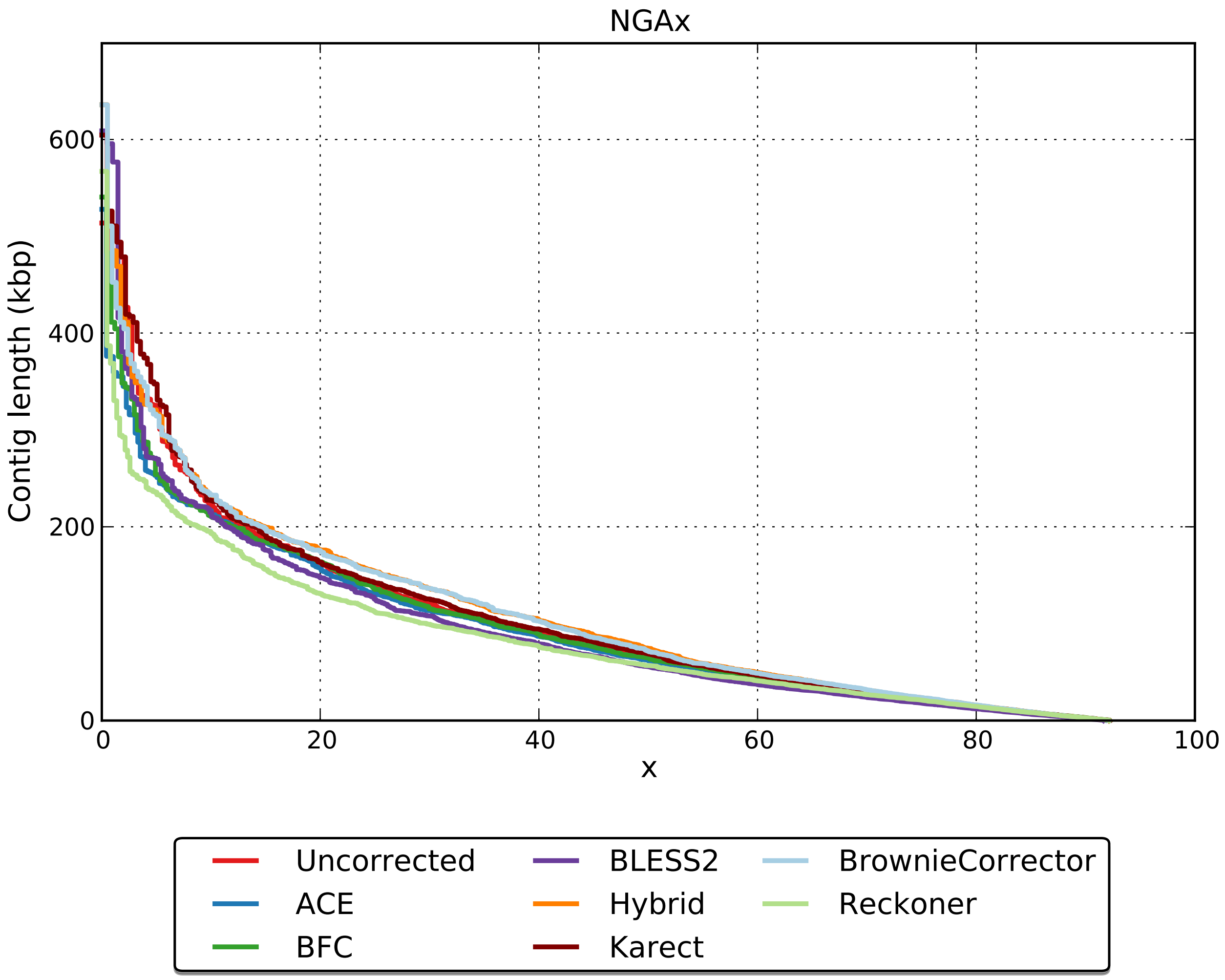

Figure 11: SPAdes assembly results for dataset D9 (*A. thaliana*) for both uncorrected and corrected data. Contigs with length NGAx or larger produce x% of the genome.

5.5 Full Quast report (scaffolds)

This section ontains the Quast evaluation report of scaffolds after assembling with each dataset with SPAdes. Error correction by ACE, BFC, BLESS2, Brownie, Karect and Reckoner are done before assembling the reads. The Uncorrected column refers to the quality of contigs without any cleaning process. The Hybrid column shows the quality of assembly of reads which are corrected jointly by BrownieCorrector and Karect. Default parameter settings are used for Quast, therefore all statistics are based on contigs of size $\geq$ 500 bp.

5.5.1 D1

Table 16 contains the Quast report after assembling dataset D1 (*Homo sapiens* Chr. 21) with SPAdes. Fig. 12 shows the corresponding NGAx plot.

Table 16: Assembly quality metrics for D1

Assembly	Uncorrected	ACE	BFC	BLESS2	Hybrid	Karect	BrownieCorrector	Reckoner
# contigs (≥ 0 bp)	15503	17167	15110	18380	16178	17003	14341	21252
# contigs (≥ 1000 bp)	3498	3309	3284	3749	2813	3063	2907	4052
# contigs (≥ 5000 bp)	1951	1912	1876	2014	1724	1826	1748	2036
# contigs (≥ 10000 bp)	1147	1130	1131	1096	1127	1133	1126	1105
# contigs (≥ 25000 bp)	251	281	281	222	344	313	338	193
# contigs (≥ 50000 bp)	25	29	32	14	56	38	52	12
Total length (≥ 0 bp)	34398184	34481028	34385863	34366932	34506906	34561810	34339292	**34763727**
Total length (≥ 1000 bp)	32641079	32638021	32675090	32371042	**32712198**	32700154	32706081	32510652
Total length (≥ 5000 bp)	28708375	28999697	29107505	27838280	**29942475**	29527995	29774056	27339748
Total length (≥ 10000 bp)	22963404	23400423	23768650	21227019	**25630161**	24533233	25273512	20609468
Total length (≥ 25000 bp)	9022632	10086964	10372635	7729872	**13215874**	11547907	12926905	6697434
Total length (≥ 50000 bp)	1506449	1830857	1990598	877864	**3485559**	2401241	3333176	726268
# contigs	4066	3815	3837	4331	3290	3561	3388	4700
Largest contig	82702	98568	**109124**	82889	105146	92853	105053	80324
Total length	33047367	32998806	**33069170**	32792092	33051550	33057202	33049826	32976233
Reference length	40988574	40988574	40988574	40988574	40988574	40988574	40988574	40988574
GC (%)	40.73	40.75	40.73	40.68	40.76	40.75	40.75	40.62
Reference GC (%)	40.93	40.93	40.93	40.93	40.93	40.93	40.93	40.93
N50	15702	16575	17050	14101	**20504**	18195	19684	13097
NG50	11992	12721	13085	10604	**15454**	14089	14798	10037
N75	8222	8669	8917	7445	**10923**	9729	10416	6938
NG75	2972	3263	3143	2600	**3857**	3531	3671	2464
L50	633	601	581	689	**492**	548	502	746
LG50	922	874	844	1025	**715**	794	734	1094
L75	1348	1276	1241	1486	**1047**	1160	1079	1599
LG75	2470	2339	2284	2806	**1907**	2114	1975	2987
# misassemblies	173	149	186	174	111	**102**	141	146
# misassembled contigs	164	137	169	165	104	**92**	132	141
Misassembled contigs length	2890519	2407563	3535938	2710908	2289710	**2152215**	2871345	2157464
# local misassemblies	219	228	**209**	247	262	248	226	228
# unaligned mis. contigs	0	0	0	0	0	0	0	0
# unaligned contigs	79 + 8 part	59 + 8 part	76 + 8 part	**47 + 9 part**	66 + 8 part	64 + 6 part	77 + 9 part	67 + 5 part
Unaligned length	86668	69281	88409	**55386**	75372	73641	86573	73305
Genome fraction (%)	80.055	79.987	80.096	79.572	**80.221**	80.188	80.201	79.865
Duplication ratio	1.004	1.004	1.005	1.004	**1.003**	1.004	**1.003**	1.005
# N's per 100 kbp	49.01	60.56	**48.55**	83.12	72.77	67.20	51.37	57.92
# mismatches per 100 kbp	166.81	163.82	162.11	164.19	**152.47**	155.03	157.12	158.29
# indels per 100 kbp	36.34	36.37	35.73	35.69	35.94	35.79	36.58	**33.18**
Largest alignment	82666	98563	96583	80972	104946	92848	**104953**	80324
Total aligned length	32885932	32847613	32911743	32670138	**32927101**	32919824	32920529	32799489
NA50	14909	15985	16161	13519	**19844**	17626	18884	12630
NGA50	11377	12135	12294	10034	**14613**	13528	14155	9670
NA75	7740	8245	8290	7020	**10287**	9290	9838	6609
NGA75	2737	2944	2867	2411	**3622**	3345	3485	2297
LA50	661	625	613	716	**509**	567	523	769
LGA50	965	912	894	1068	**742**	823	766	1131
LA75	1416	1336	1317	1553	**1090**	1204	1130	1659
LGA75	2617	2463	2438	2959	**1999**	2206	2083	3127

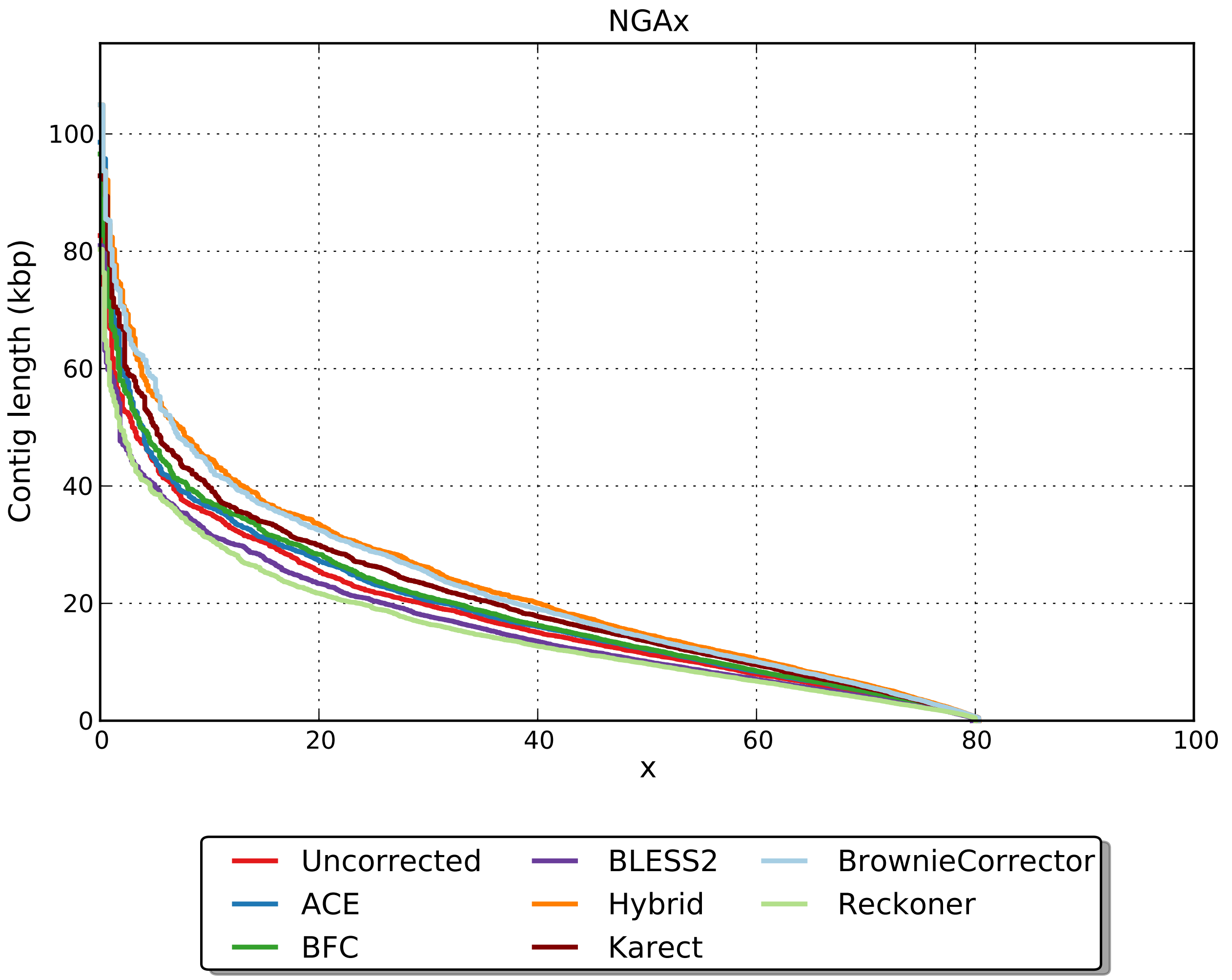

Figure 12: SPAdes assembly results for dataset D1 (*Homo sapiens* Chr. 21) for both uncorrected and corrected data. Contigs with length NGAx or larger produce x% of the genome.

5.5.2 D2

Table 17 contains the Quast report after assembling dataset D2 (*Homo sapiens* Chr. 14) with SPAdes. Fig. 13 shows the corresponding NGAx plot.

Table 17: Assembly quality metrics for D2

Assembly	Uncorrected	ACE	BFC	BLESS2	Hybrid	Karect	BrownieCorrector	Reckoner
# contigs (≥ 0 bp)	412300	63904	61280	65498	54926	59015	85814	82873
# contigs (≥ 1000 bp)	14739	10310	9552	11193	7617	9004	8477	12975
# contigs (≥ 5000 bp)	5460	5223	5064	5399	4603	4971	4930	5473
# contigs (≥ 10000 bp)	2137	2714	2776	2629	2798	2742	2792	2424
# contigs (≥ 25000 bp)	184	503	600	408	804	655	710	294
# contigs (≥ 50000 bp)	4	42	65	27	134	76	95	14
Total length (≥ 0 bp)	**111749252**	88521825	88702085	88492512	88275916	88464779	90639963	89672578
Total length (≥ 1000 bp)	81372306	82954897	83332150	82608365	**83614800**	83395425	83495313	82247586
Total length (≥ 5000 bp)	57788314	69389231	71436004	67364079	**75477644**	72512288	74019938	62869914
Total length (≥ 10000 bp)	34244755	51349355	54876683	47470113	**62391757**	56434088	58583750	41338299
Total length (≥ 25000 bp)	5779031	17392790	21535238	13906135	**31052540**	23670662	26095142	9724112
Total length (≥ 50000 bp)	224901	2538630	3934138	1701829	**8524714**	4647418	5858991	830500
# contigs	17855	11720	10815	12831	8522	10136	9543	15158
Largest contig	68123	100666	**126928**	97813	102135	100683	100685	81231
Total length	83643950	83985139	84256551	83807676	**84267170**	84217172	84265213	83842223
Reference length	107349540	107349540	107349540	107349540	107349540	107349540	107349540	107349540
GC (%)	40.66	40.71	40.71	40.68	40.73	40.72	40.73	40.48
Reference GC (%)	40.89	40.89	40.89	40.89	40.89	40.89	40.89	40.89
N50	8250	13067	14341	11696	**18672**	15461	16361	9825
NG50	5758	9330	10372	8410	**13471**	10946	11777	6921
N75	4159	6750	7542	6069	**9688**	7877	8480	4998
NG75	1185	1918	2157	1702	**2806**	2303	2481	1455
L50	2974	1894	1708	2114	**1317**	1593	1510	2483
LG50	4693	2955	2659	3304	**2045**	2479	2341	3909
L75	6542	4125	3729	4583	**2881**	3499	3293	5476
LG75	13948	8606	7721	9619	**5908**	7222	6700	11552
# misassemblies	119	822	640	720	354	496	**112**	689
# misassembled contigs	119	760	612	679	337	469	**110**	660
Misassembled contigs length	**997983**	10744993	9204509	8534721	7092262	7896311	2186146	6895634
# local misassemblies	480	150	186	165	176	176	349	**132**
# unaligned mis. contigs	**0**	1	**0**	**0**	**0**	**0**	**0**	**0**
# unaligned contigs	**11 + 8 part**	12 + 27 part	14 + 22 part	19 + 19 part	14 + 16 part	13 + 22 part	16 + 5 part	17 + 26 part
Unaligned length	16677	35163	31344	35033	27906	29729	**16274**	38932
Genome fraction (%)	77.427	77.786	78.123	77.707	**78.327**	78.150	78.313	77.523
Duplication ratio	1.006	1.005	1.004	1.004	**1.002**	1.004	**1.002**	1.007
# N's per 100 kbp	59.38	19.79	23.88	20.83	23.75	23.18	44.74	**17.91**
# mismatches per 100 kbp	112.08	127.07	119.92	125.10	105.69	114.90	**101.65**	118.33
# indels per 100 kbp	20.59	21.61	21.57	20.78	21.54	21.28	20.80	**17.48**
Largest alignment	68123	100531	**126784**	93310	102135	100548	100547	70727
Total aligned length	83424246	83718057	84046502	83630149	**84181506**	84037475	84168916	83423730
NA50	8159	12005	13492	11050	**17885**	14695	16172	9345
NGA50	5668	8597	9698	7909	**12795**	10298	11570	6509
NA75	4106	6173	6949	5671	**9168**	7437	8356	4663
NGA75	1126	1714	1953	1553	**2629**	2138	2413	1293
LA50	2996	2021	1796	2222	**1372**	1669	1530	2584
LGA50	4738	3173	2810	3480	**2133**	2612	2374	4091
LA75	6614	4448	3962	4848	**3010**	3693	3339	5757
LGA75	14206	9372	8300	10283	**6202**	7670	6814	12352

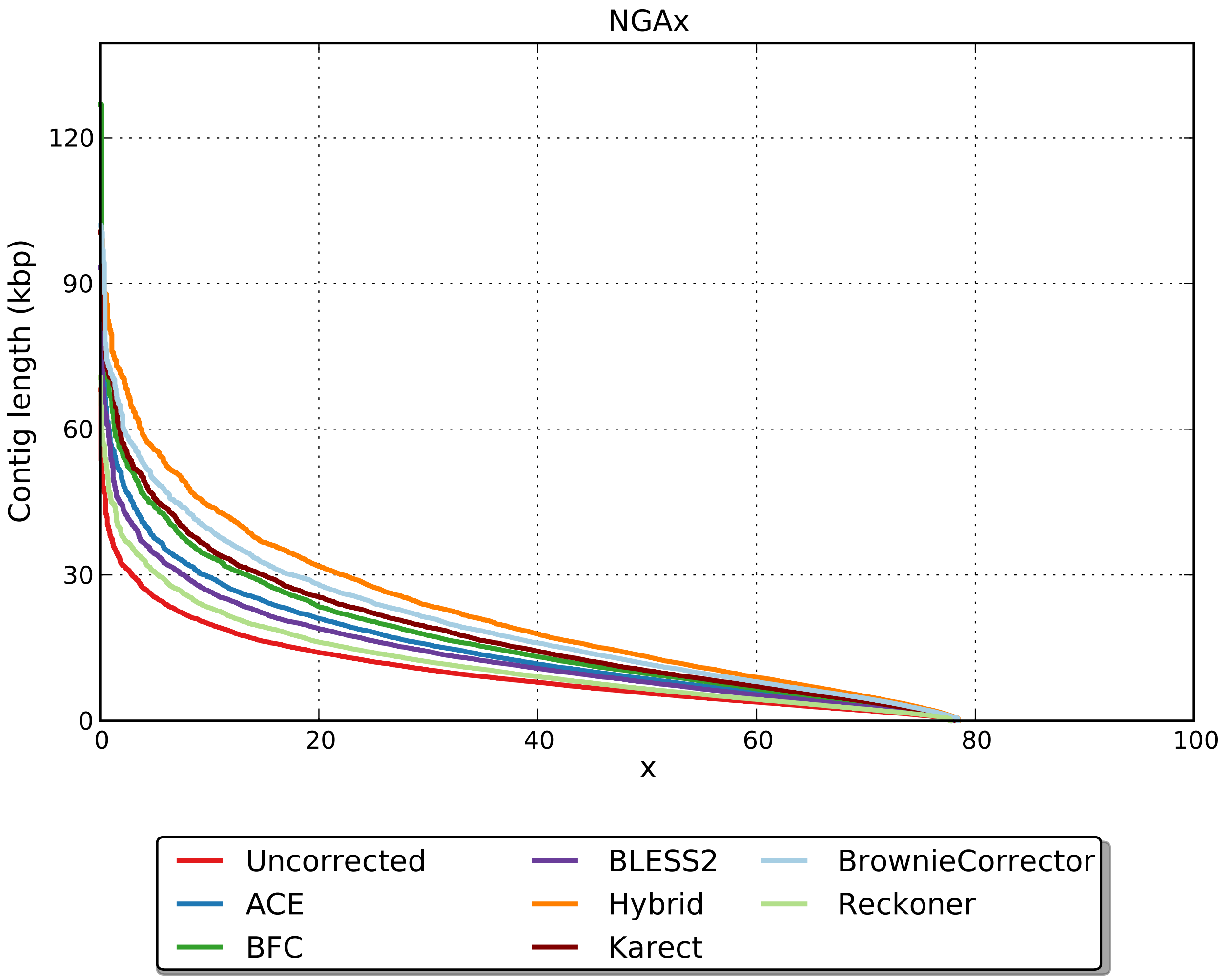

Figure 13: SPAdes assembly results for dataset D2 (*Homo sapiens* Chr. 14) for both uncorrected and corrected data. Contigs with length NGAx or larger produce x% of the genome.

5.5.3 D3

Table 18 contains the Quast report after assembling dataset D3 (*C. elegans*) with SPAdes. Fig. 14 shows the corresponding NGAx plot.

Table 18: Assembly quality metrics for D3

Assembly	Uncorrected	ACE	BFC	BLESS2	Hybrid	Karect	BrownieCorrector	Reckoner
# contigs (≥ 0 bp)	90335	107935	90891	116183	91250	91070	90434	93218
# contigs (≥ 1000 bp)	19533	25744	19588	27553	19590	19574	19528	19596
# contigs (≥ 5000 bp)	6275	5301	6295	4769	6332	6328	6274	6262
# contigs (≥ 10000 bp)	2407	1217	2398	974	2435	2431	2408	2399
# contigs (≥ 25000 bp)	287	122	302	74	308	310	287	293
# contigs (≥ 50000 bp)	68	23	68	8	59	59	68	66
Total length (≥ 0 bp)	116427720	112565978	116454662	112409711	116147212	116129035	116431324	**116491367**
Total length (≥ 1000 bp)	**104025257**	95726723	103891921	92919344	103553564	103556644	104024612	103767981
Total length (≥ 5000 bp)	72097772	47758221	71982022	40520190	71740186	71720794	**72099815**	71747596
Total length (≥ 10000 bp)	44967590	19948617	44604367	14894822	44301236	44247396	**44980157**	44564052
Total length (≥ 25000 bp)	13938243	5143754	**13939203**	2681884	13170145	13249871	13925728	13879239
Total length (≥ 50000 bp)	6929307	1749408	6340634	478678	5235685	5239938	**6929310**	6619857
# contigs	25948	36175	26126	39647	26140	26112	25944	26150
Largest contig	**244078**	128379	240394	80553	238991	238991	**244078**	240969
Total length	108653017	103255711	108611564	101595435	108285144	108279807	**108653330**	108501463
Reference length	100286070	100286070	100286070	100286070	100286070	100286070	100286070	100286070
GC (%)	38.47	38.30	38.46	38.20	38.41	38.41	38.47	38.46
Reference GC (%)	35.44	35.44	35.44	35.44	35.44	35.44	35.44	35.44
N50	7969	4582	7916	3962	7923	7932	**7971**	7922
NG50	**8827**	4746	8788	4025	8738	8752	**8827**	8779
N75	3702	2326	3682	2037	3674	3679	**3704**	3670
NG75	**4553**	2491	4521	2100	4481	4484	**4553**	4491
L50	3458	6109	3489	7085	3541	3541	**3457**	3488
LG50	**2958**	5791	2990	6921	3061	3062	**2958**	2995
L75	8459	14033	8509	16035	8547	8544	**8457**	8514
LG75	6929	13108	6975	15560	7067	7067	**6928**	6995
# misassemblies	1250	4509	1212	1497	**1207**	1237	1251	1235
# misassembled contigs	1193	4044	1158	1445	**1147**	1174	1195	1178
Misassembled contigs length	9261752	18237042	9018821	**5602595**	9086566	9223611	9254187	9177693
# local misassemblies	402	328	393	**307**	410	412	401	411
# unaligned mis. contigs	4	9	4	3	**2**	**2**	4	3
# unaligned contigs	4505 + 68 part	**3840 + 129 part**	4553 + 54 part	4189 + 58 part	4615 + 58 part	4615 + 59 part	4506 + 68 part	4538 + 65 part
Unaligned length	16604720	14149412	16541258	**13702622**	16331635	16333588	16605131	16539717
Genome fraction (%)	91.309	86.978	**91.329**	87.304	91.240	91.241	91.311	91.232
Duplication ratio	1.005	1.022	1.005	**1.004**	1.005	1.005	1.005	1.005
# N's per 100 kbp	64.44	**58.26**	65.02	63.88	65.48	65.58	64.43	64.62
# mismatches per 100 kbp	23.77	105.67	**23.20**	27.49	23.49	23.43	23.67	24.32
# indels per 100 kbp	6.02	18.39	**5.94**	7.10	6.04	6.09	6.01	6.09
Largest alignment	54032	27212	54032	27155	**64367**	**64367**	54032	54032
Total aligned length	91830875	87408611	**91862827**	87715060	91758254	91749370	91831029	91755248
NA50	5684	3013	5674	2959	**5687**	5681	5681	5643
NGA50	6419	3143	6392	3012	6380	6377	**6420**	6354
NA75	1935	1150	1933	1238	1960	**1962**	1937	1917
NGA75	2753	1306	2739	1299	2729	2729	**2754**	2711
LA50	4987	9285	4997	9468	**4974**	4975	4986	5018
LGA50	4294	8803	4305	9249	4310	4311	**4293**	4332
LA75	12939	22780	12960	22529	**12871**	**12871**	12936	13022
LGA75	10236	20964	10265	21754	10291	10293	**10234**	10340

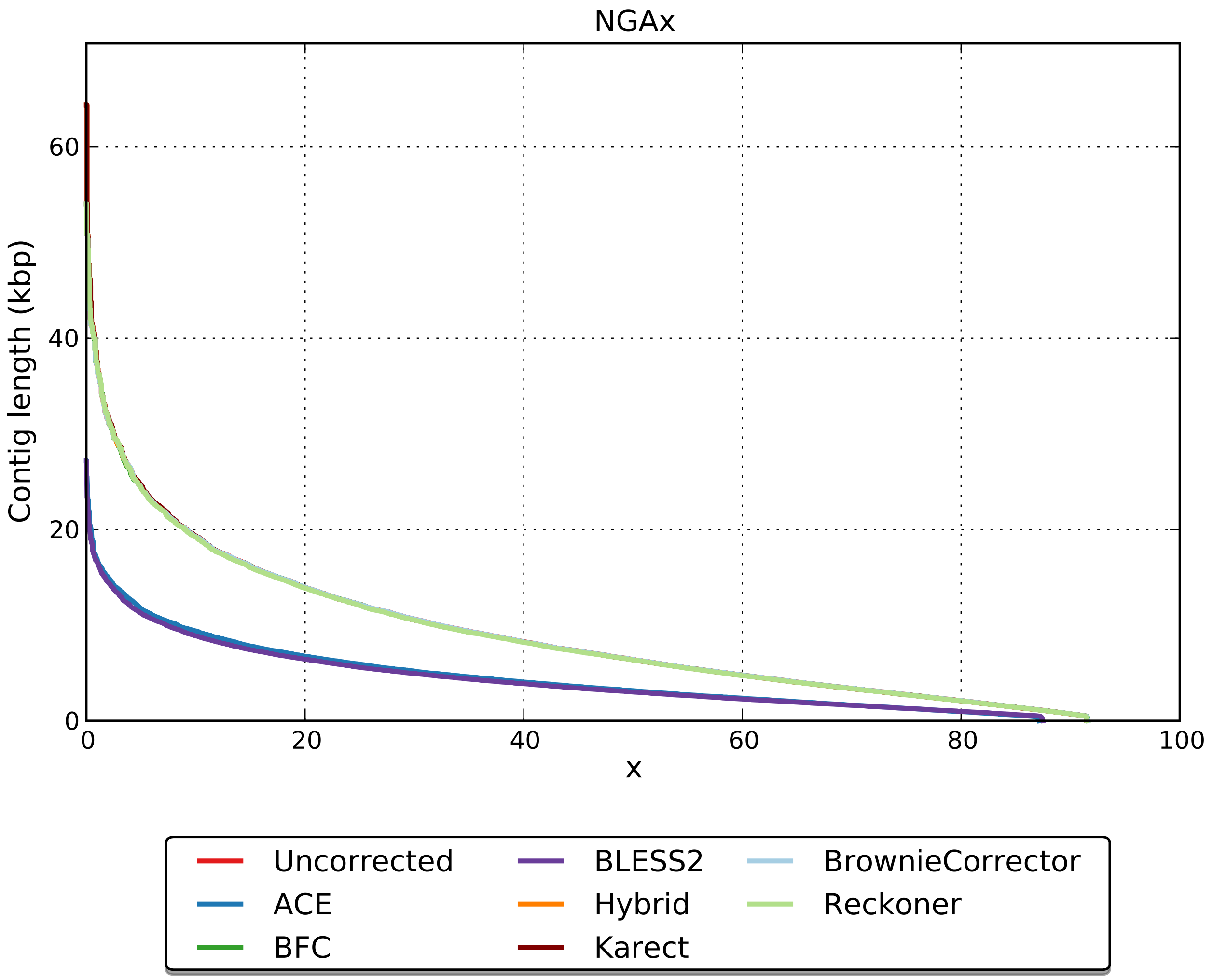

Figure 14: SPAdes assembly results for dataset D3 (*C. elegans*) for both uncorrected and corrected data. Contigs with length NGAx or larger produce x% of the genome.

5.5.4 D4

Table 19 contains the Quast report after assembling dataset D4 (*D. melanogaster*) with SPAdes. Fig. 15 shows the corresponding NGAx plot.

Table 19: Assembly quality metrics for D4

Assembly	Uncorrected	ACE	BFC	BLESS2	Hybrid	Karect	BrownieCorrector	Reckoner
# contigs (≥ 0 bp)	93227	97585	91434	95653	91669	92410	93123	101162
# contigs (≥ 1000 bp)	5191	6530	5222	6405	5061	5110	5139	6011
# contigs (≥ 5000 bp)	2504	3776	2571	3704	2366	2422	2467	3104
# contigs (≥ 10000 bp)	1928	2775	1971	2737	1812	1849	1889	2305
# contigs (≥ 25000 bp)	1208	1435	1220	1402	1162	1177	1202	1331
# contigs (≥ 50000 bp)	704	624	689	608	682	692	706	692
Total length (≥ 0 bp)	130032928	128403487	129857535	127084174	129839690	129910273	130015593	**130535397**
Total length (≥ 1000 bp)	120113313	118405134	120103149	117350466	120183371	**120186079**	120097364	120089504
Total length (≥ 5000 bp)	114298074	111900442	114332150	110932876	114383380	**114416230**	114335355	113588596
Total length (≥ 10000 bp)	110173410	104680036	110024527	104013656	**110404451**	110319450	110191218	107874824
Total length (≥ 25000 bp)	98369482	82466621	97678502	82061737	**99836443**	99349015	98927274	92069860
Total length (≥ 50000 bp)	79975935	53718772	78310043	54010198	**82227505**	81465937	80907051	69202110
# contigs	7662	8866	7718	8367	7424	7491	7606	8532
Largest contig	518264	333642	518140	439905	517988	517989	518142	**557849**
Total length	121842729	120028744	121845718	118703348	121839229	**121857032**	121823758	121851136
Reference length	120381546	120381546	120381546	120381546	120381546	120381546	120381546	120381546
GC (%)	42.58	42.53	42.58	42.52	42.59	42.59	42.58	42.56
Reference GC (%)	42.42	42.42	42.42	42.42	42.42	42.42	42.42	42.42
N50	83828	43422	80542	44025	**91990**	89577	85463	60794
NG50	85463	43357	82483	43192	**93214**	90787	86717	62202
N75	35616	20093	33969	20060	**37814**	37416	35886	25734
NG75	36690	19951	35304	19140	**39194**	38652	37394	26815
L50	410	759	413	722	**373**	385	401	542
LG50	401	763	404	741	**365**	377	393	530
L75	975	1772	1005	1712	**892**	921	952	1305
LG75	945	1785	974	1777	**864**	892	923	1263
# misassemblies	832	974	844	1006	818	**814**	822	895
# misassembled contigs	622	799	628	823	**610**	616	619	695
Misassembled contigs length	50774126	**37019339**	50754826	38660485	54140750	52300325	51539311	46615389
# local misassemblies	1884	1748	1931	2074	**1732**	1784	1817	1795
# unaligned mis. contigs	29	**21**	28	23	33	32	29	23
# unaligned contigs	3774 + 386 part	2980 + 439 part	3762 + 392 part	**2371 + 381 part**	3721 + 387 part	3739 + 375 part	3772 + 386 part	3774 + 380 part
Unaligned length	8286358	6648237	8296140	**5569650**	8335405	8336511	8284422	8273018
Genome fraction (%)	93.917	93.629	93.912	93.537	93.916	**93.918**	93.914	93.893
Duplication ratio	**1.004**	1.006	**1.004**	1.005	**1.004**	**1.004**	**1.004**	1.005
# N's per 100 kbp	26.10	27.40	25.99	62.44	**24.89**	25.09	25.66	24.99
# mismatches per 100 kbp	547.86	562.17	547.37	559.26	**545.82**	546.09	546.03	546.99
# indels per 100 kbp	130.96	137.03	130.85	134.63	131.23	131.10	130.85	**130.38**
Largest alignment	**446262**	237405	428080	285020	446086	446075	446204	375337
Total aligned length	**113196227**	112863314	113184786	112744813	113175593	113182356	113188396	113182400
NA50	59641	35523	57984	35534	**64663**	62025	60667	46786
NGA50	60714	35425	59124	34856	**65857**	63400	61474	47781
NA75	25472	16277	24546	16539	**27792**	27188	26490	19823
NGA75	26604	16100	25824	15878	**29067**	28124	27588	20635
LA50	560	939	570	906	**524**	533	554	712
LGA50	548	944	557	930	**513**	521	543	697
LA75	1335	2191	1370	2133	**1243**	1265	1314	1707
LGA75	1293	2207	1326	2211	**1205**	1225	1274	1653

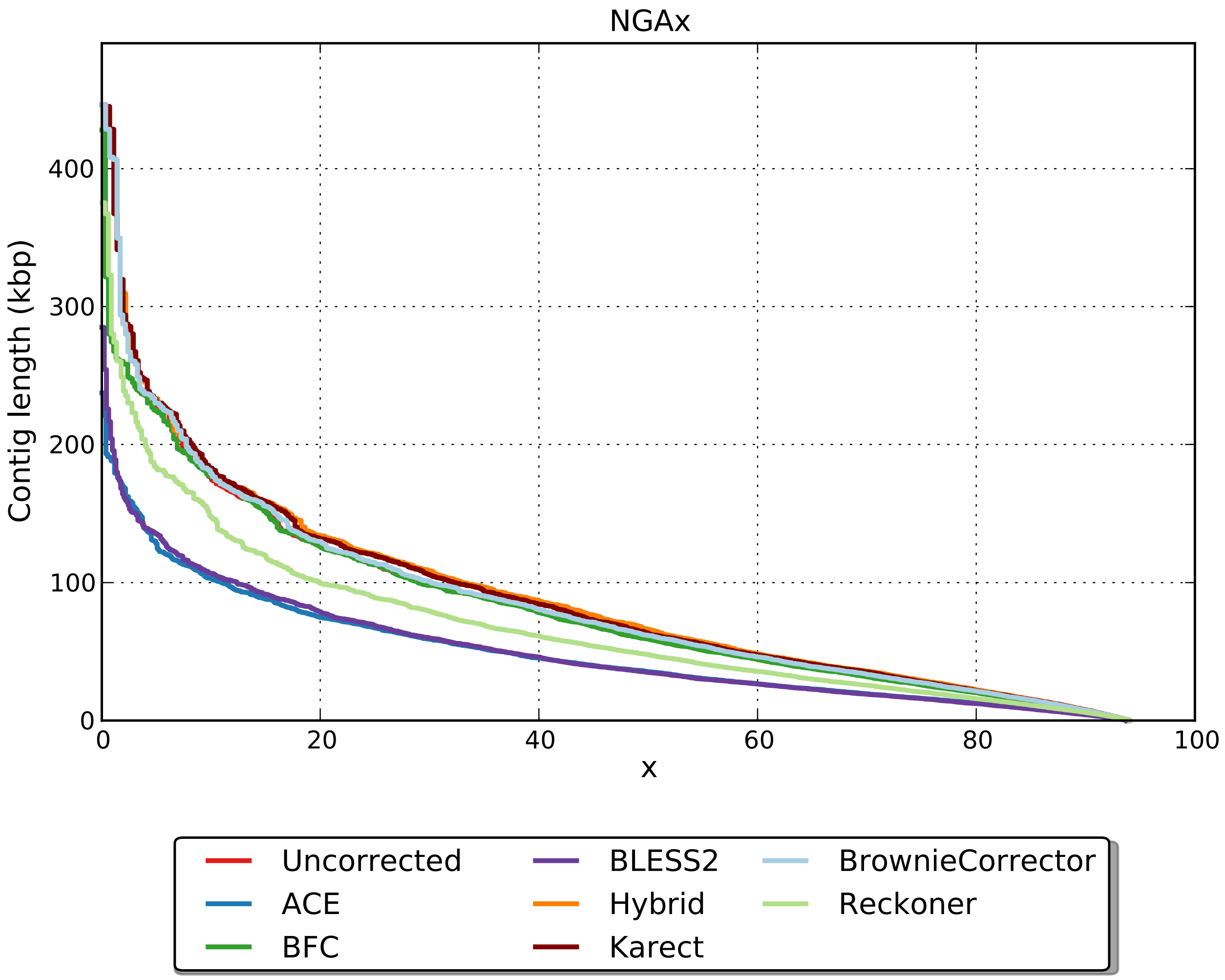

Figure 15: SPAdes assembly results for dataset D4 (*D. melanogaster*) for both uncorrected and corrected data. Contigs with length NGAx or larger produce x% of the genome.

5.5.5 D5

Table 20 contains the Quast report after assembling dataset D5 (*D. melanogaster*) with SPAdes. Fig. 16 shows the corresponding NGAx plot.

Table 20: Assembly quality metrics for D5

Assembly	Uncorrected	ACE	BFC	BLESS2	Hybrid	Karect	BrownieCorrector	Reckoner
# contigs (≥ 0 bp)	165126	212433	185876	197317	181524	182557	164967	217940
# contigs (≥ 1000 bp)	4789	5594	5055	6310	4560	4762	4482	5243
# contigs (≥ 5000 bp)	2621	3264	2845	3636	2467	2619	2394	2970
# contigs (≥ 10000 bp)	1936	2476	2100	2698	1833	1937	1778	2193
# contigs (≥ 25000 bp)	1238	1412	1300	1401	1206	1243	1178	1334
# contigs (≥ 50000 bp)	729	660	724	620	742	737	731	724
Total length (≥ 0 bp)	136727305	137556107	138577682	136206452	138250189	138294938	136743703	**140818635**
Total length (≥ 1000 bp)	120037979	116819984	119820170	117054224	119768316	119758171	**120045889**	119817827
Total length (≥ 5000 bp)	114951585	111441304	114683614	110797759	114967414	114815029	**115192459**	114525105
Total length (≥ 10000 bp)	110058903	105831032	109406428	104056408	110451440	109964263	**110829083**	108944878
Total length (≥ 25000 bp)	98578883	88160152	96224991	82586917	100140538	98566386	**101001837**	94721840
Total length (≥ 50000 bp)	80201757	61195626	75734377	54589201	83311554	80477513	**84855860**	72897465
# contigs	7564	8407	8070	8848	7512	7720	7250	8305
Largest contig	635766	633553	593823	382830	693535	573416	**790574**	615528
Total length	122075582	118882582	122043781	118934797	121947926	121943277	**122079245**	122072919
Reference length	120381546	120381546	120381546	120381546	120381546	120381546	120381546	120381546
GC (%)	42.47	42.53	42.49	42.50	42.51	42.50	42.48	42.48
Reference GC (%)	42.42	42.42	42.42	42.42	42.42	42.42	42.42	42.42
N50	79091	52325	68696	45242	83768	79386	**90787**	65116
NG50	81525	51206	70464	44409	85018	80160	**92829**	65909
N75	34445	24360	30032	20786	38515	34907	**39744**	28474
NG75	36491	23546	31238	19878	39914	36153	**41573**	29856
L50	421	626	474	723	397	425	**375**	516
LG50	411	641	462	740	388	416	**366**	503
L75	1001	1453	1130	1692	929	1001	**882**	1216
LG75	965	1500	1089	1746	899	968	**850**	1173
# misassemblies	762	831	766	780	756	757	**754**	789
# misassembled contigs	588	698	601	671	**570**	587	575	631
Misassembled contigs length	49650199	40548248	45881007	**34458785**	50816213	49036345	54113900	45207902
# local misassemblies	**2588**	4259	3458	4442	3421	3420	**2588**	3510
# unaligned mis. contigs	27	31	28	**18**	28	31	26	31
# unaligned contigs	3627 + 370 part	2541 + 408 part	3559 + 384 part	**2410 + 375 part**	3531 + 359 part	3530 + 361 part	3629 + 365 part	3591 + 373 part
Unaligned length	8517557	**5527503**	8554387	5579538	8439808	8436001	8508422	8522280
Genome fraction (%)	93.780	93.289	93.578	93.214	93.584	93.594	**93.788**	93.581
Duplication ratio	**1.006**	1.009	1.007	1.010	1.008	1.007	**1.006**	1.008
# N's per 100 kbp	589.55	**565.60**	718.48	729.81	730.60	728.54	591.23	721.68
# mismatches per 100 kbp	500.54	500.48	497.06	502.65	**494.14**	495.59	497.83	496.90
# indels per 100 kbp	119.70	119.15	118.61	118.81	118.54	118.77	119.42	**118.52**
Largest alignment	402868	365720	385170	333479	385163	385170	**402877**	385169
Total aligned length	113080516	112542712	112866314	112513234	112914086	112899477	**113107293**	112915266
NA50	58252	41363	53544	36812	61763	58806	**63652**	50153
NGA50	59591	40860	54093	36316	62706	59526	**65174**	50834
NA75	25864	19605	23565	16968	27787	25860	**28871**	21977
NGA75	27153	18756	24911	16471	29033	26852	**30231**	23124
LA50	574	786	629	877	549	577	**527**	667
LGA50	559	805	613	897	536	564	**513**	651
LA75	1346	1814	1479	2056	1268	1345	**1218**	1579
LGA75	1298	1872	1427	2121	1226	1301	**1175**	1523

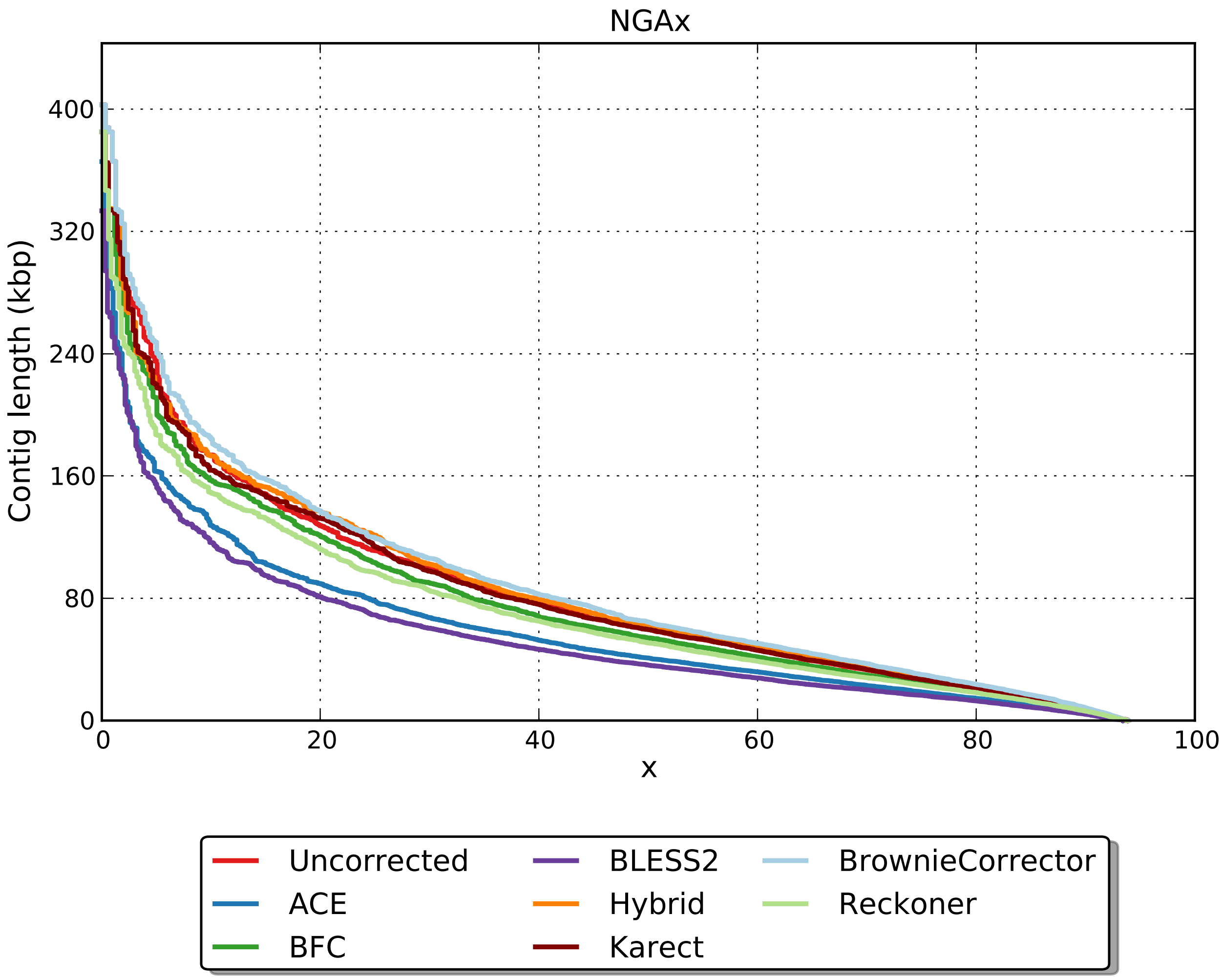

Figure 16: SPAdes assembly results for dataset D5 (*D. melanogaster*) for both uncorrected and corrected data. Contigs with length NGAx or larger produce x% of the genome.

5.5.6 D6

Table 21 contains the Quast report after assembling dataset D6 (*A. thaliana*) with SPAdes. Fig. 17 shows the corresponding NGAx plot.

Table 21: Assembly quality metrics for D6

Assembly	Uncorrected	ACE	BFC	BLESS2	Hybrid	Karect	BrownieCorrector	Reckoner
# contigs (≥ 0 bp)	153290	168992	152296	157691	156284	157079	153194	168975
# contigs (≥ 1000 bp)	5162	5322	5106	5472	4889	5100	4900	5279
# contigs (≥ 5000 bp)	3342	3431	3329	3432	3145	3295	3141	3402
# contigs (≥ 10000 bp)	2489	2529	2478	2497	2360	2462	2349	2529
# contigs (≥ 25000 bp)	1322	1269	1302	1282	1275	1294	1277	1300
# contigs (≥ 50000 bp)	598	575	596	565	611	593	615	599
Total length (≥ 0 bp)	122801905	123596492	122790209	122366708	123037135	123098372	122790637	**123961124**
Total length (≥ 1000 bp)	108714303	108474907	**108842601**	107970602	108759997	108767495	108713396	108797092
Total length (≥ 5000 bp)	104203084	103753669	**104453011**	102836224	104451806	104264416	104377293	104095523
Total length (≥ 10000 bp)	98015647	97195298	98261766	96043789	**98726415**	98203437	98629627	97749838
Total length (≥ 25000 bp)	79007005	76716663	78935185	76403311	80964898	79112155	**81203481**	77458191
Total length (≥ 50000 bp)	53364388	52047704	53919340	51174509	57276588	54231397	**57675590**	52534096
# contigs	6616	6774	6501	6946	6304	6539	6326	6708
Largest contig	378346	360514	337032	340077	**403164**	358676	360062	292751
Total length	109698912	109456349	**109787184**	108964952	109717059	109740727	109679469	109761199
Reference length	119668634	119668634	119668634	119668634	119668634	119668634	119668634	119668634
GC (%)	35.96	35.98	35.96	35.99	35.97	35.96	35.96	35.96
Reference GC (%)	36.06	36.06	36.06	36.06	36.06	36.06	36.06	36.06
N50	48674	47051	49223	46312	52900	49483	**53537**	47973
NG50	43039	41281	43661	40388	47318	43958	**47965**	41999
N75	22586	21015	22561	20636	24149	22647	**24323**	21691
NG75	16229	15179	16601	14482	**17410**	16410	17316	15906
L50	629	631	616	634	565	606	**561**	647
LG50	737	747	723	758	664	713	**660**	758
L75	1460	1503	1446	1517	1329	1428	**1320**	1508
LG75	1850	1934	1827	1979	1691	1811	**1685**	1906
# misassemblies	157	151	167	194	**123**	140	126	143
# misassembled contigs	156	148	163	186	**122**	134	125	138
Misassembled contigs length	7195789	6683457	8790317	7948697	**5512136**	6420888	5809898	6525648
# local misassemblies	91	95	**83**	107	90	89	86	**83**
# unaligned mis. contigs	2	2	2	**1**	**1**	2	2	2
# unaligned contigs	234 + 21 part	161 + 24 part	218 + 24 part	**104 + 34 part**	225 + 27 part	229 + 27 part	233 + 22 part	231 + 25 part
Unaligned length	409655	245951	410530	**200169**	400587	402363	407487	411574
Genome fraction (%)	91.209	91.115	**91.285**	90.734	91.256	91.258	91.219	91.257
Duplication ratio	**1.001**	1.002	**1.001**	1.002	**1.001**	**1.001**	**1.001**	**1.001**
# N's per 100 kbp	11.00	24.65	10.48	93.43	12.59	12.23	10.39	**10.08**
# mismatches per 100 kbp	20.15	31.85	19.74	22.56	18.61	19.74	**18.31**	19.02
# indels per 100 kbp	5.08	9.70	5.01	7.65	4.87	4.89	**4.84**	4.92
Largest alignment	378346	360476	322214	340077	**402910**	358628	360023	292696
Total aligned length	109238189	109114747	**109327444**	108666168	109275728	109291520	109231090	109296620
NA50	47023	45327	47171	44605	51586	48129	**52551**	46233
NGA50	41833	39895	41818	38431	46332	42256	**46678**	40779
NA75	21722	20139	21736	19707	23677	21873	**23679**	21161
NGA75	15581	14730	15864	13950	**16845**	15760	16687	15390
LA50	647	650	642	657	575	621	**573**	666
LGA50	759	770	752	786	677	731	**674**	781
LA75	1503	1556	1503	1577	1356	1467	**1351**	1554
LGA75	1909	2001	1900	2058	**1728**	1865	**1728**	1963

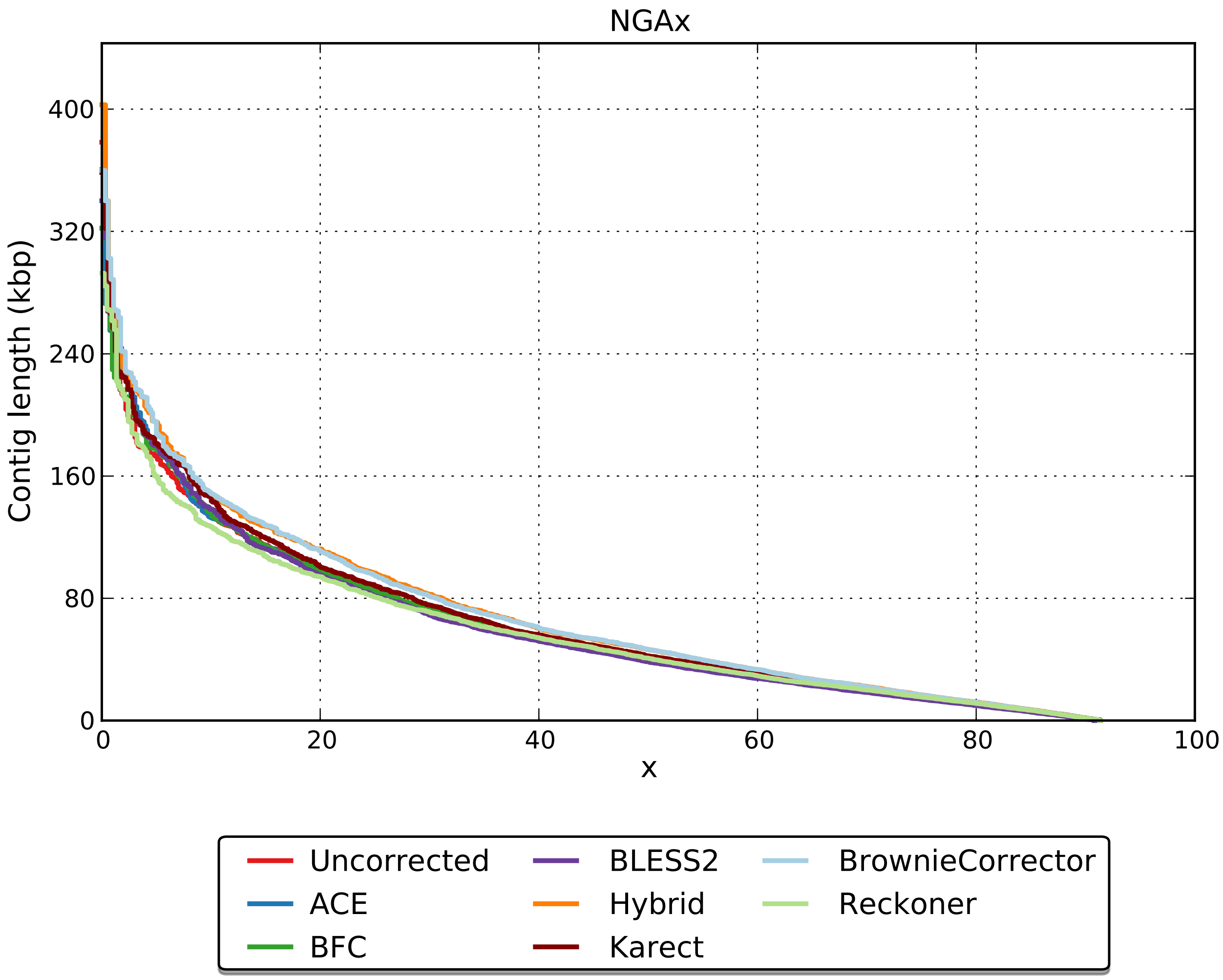

Figure 17: SPAdes assembly results for dataset D6 (*D. melanogaster*) for both uncorrected and corrected data. Contigs with length NGAx or larger produce x% of the genome.

5.5.7 D7

Table 22 contains the Quast report after a hybrid assembly of dataset D7 (*D. melanogaster*) with SPAdes. This is a hybrid assembly in which the corrected (and uncorrected) Illumina reads (R4) are complemented with the Pacbio reads (P1). Fig. 18 shows the corresponding NGAx plot.

Table 22: Assembly quality metrics for D7

Assembly	Uncorrected	ACE	BFC	BLESS2	Hybrid	Karect	BrownieCorrector	Reckoner
# contigs (≥ 0 bp)	82728	86644	81601	84454	81455	82018	82726	90888
# contigs (≥ 1000 bp)	3570	4035	3705	3493	3545	3566	3538	4403
# contigs (≥ 5000 bp)	1704	2416	1799	2032	1614	1658	1694	2334
# contigs (≥ 10000 bp)	1314	1857	1369	1571	1202	1229	1285	1751
# contigs (≥ 25000 bp)	853	1133	904	1024	798	818	854	1104
# contigs (≥ 50000 bp)	579	694	605	658	554	570	574	678
Total length (≥ 0 bp)	131187203	129316221	130842102	128194970	130823725	130846045	131147292	**131587165**
Total length (≥ 1000 bp)	**122759330**	120824487	122515719	119969315	122632419	122599419	122716522	122605532
Total length (≥ 5000 bp)	118723698	116992547	118417293	116381572	118516483	118516474	**118760696**	118026807
Total length (≥ 10000 bp)	**115939340**	113005485	115325687	113095422	115563382	115456277	115866004	113848935
Total length (≥ 25000 bp)	108413703	101200875	107777428	104320159	**108945714**	108786024	108922843	103351617
Total length (≥ 50000 bp)	98491977	85458576	96979047	91176824	**100102272**	99794613	98871234	88326978
# contigs	5317	5576	5498	4611	5199	5232	5291	6207
Largest contig	**1199604**	670790	907732	602677	1037537	908356	1016904	887190
Total length	**123986725**	121887615	123769629	120729772	123793924	123771242	123945141	123866970
Reference length	120381546	120381546	120381546	120381546	120381546	120381546	120381546	120381546
GC (%)	42.55	42.50	42.55	42.46	42.56	42.56	42.55	42.53
Reference GC (%)	42.42	42.42	42.42	42.42	42.42	42.42	42.42	42.42
N50	169610	97526	159663	125811	**184981**	174552	173854	104119
NG50	179224	99802	165472	126462	**189080**	179261	179844	108234
N75	65173	39922	58324	50582	**73720**	70434	68958	41468
NG75	74095	41210	65771	51453	**86411**	80784	79734	46078
L50	198	343	214	262	**183**	188	198	314
LG50	187	335	203	260	**174**	178	187	298
L75	483	829	529	646	**434**	453	472	779
LG75	444	801	487	641	**402**	419	436	719
# misassemblies	1093	1198	1095	1254	1058	**1049**	1073	1190
# misassembled contigs	576	751	568	703	**548**	549	570	691
Misassembled contigs length	77195891	**65434373**	74113978	72211756	79124616	77842226	77576813	65900369
# local misassemblies	1912	**1762**	1886	1911	1802	1851	1871	2032
# unaligned mis. contigs	140	127	135	**105**	125	132	129	137
# unaligned contigs	3051 + 699 part	2271 + 771 part	3086 + 724 part	**1719 + 662 part**	3061 + 730 part	3047 + 735 part	3057 + 702 part	3103 + 746 part
Unaligned length	9623179	7729395	9471622	**6671029**	9531780	9527862	9589547	9494184
Genome fraction (%)	94.484	94.275	94.474	94.248	94.478	94.456	**94.486**	94.431
Duplication ratio	**1.005**	1.006	**1.005**	**1.005**	**1.005**	**1.005**	**1.005**	1.006
# N's per 100 kbp	483.18	**408.89**	460.28	422.82	447.36	439.87	475.04	449.96
# mismatches per 100 kbp	556.84	567.59	556.70	562.67	554.80	**553.80**	555.55	556.33
# indels per 100 kbp	134.33	141.80	134.28	145.24	133.75	133.66	134.05	**133.40**
Largest alignment	570650	428847	443438	338930	570790	**570959**	570669	406192
Total aligned length	**114102548**	113780243	114027727	113763228	114012129	113990788	114097579	114030717
NA50	92022	61834	88388	73292	**99412**	97727	92238	64742
NGA50	96381	62981	91577	73377	**103872**	101753	96385	67061
NA75	37711	26304	35621	32030	**42031**	40768	38469	27161
NGA75	41779	27692	39269	32305	**45314**	44500	42580	29658
LA50	373	538	390	462	**352**	358	369	514
LGA50	354	526	371	460	**335**	341	350	487
LA75	894	1281	940	1079	**829**	848	883	1243
LGA75	826	1240	872	1071	**771**	789	817	1151

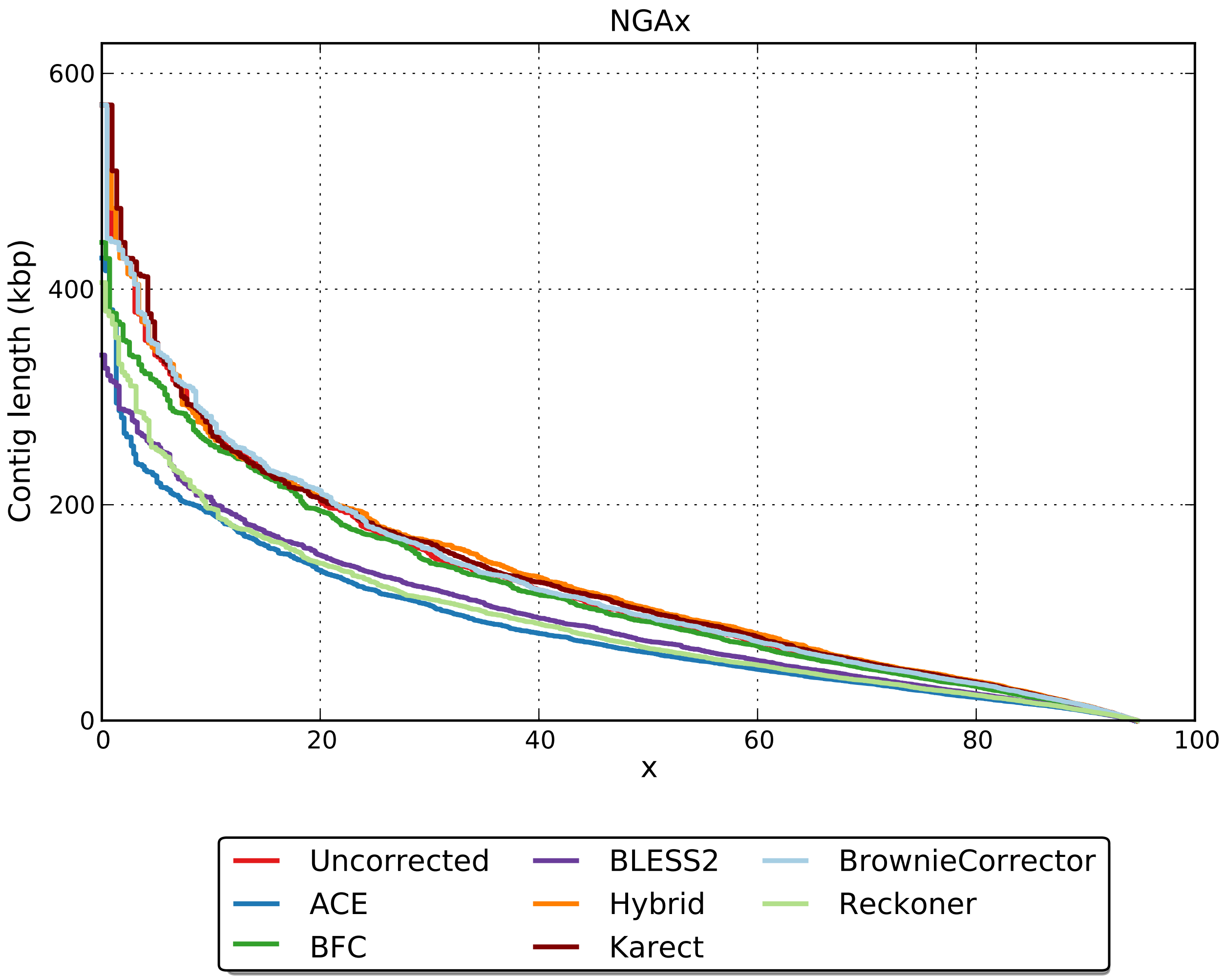

Figure 18: SPAdes assembly results for dataset D7 (*D. melanogaster*) for both uncorrected and corrected data. Contigs with length NGAx or larger produce x% of the genome.

5.5.8 D8

Table 23 contains the Quast report after assembling dataset D8 *D. melanogaster* with SPAdes. This is a hybrid assembly in which the corrected (and uncorrected) Illumina reads (R5) are complemented with the Pacbio reads (P1). Fig. 19 shows the corresponding NGAx plot.

Table 23: Assembly quality metrics for D8

Assembly	Uncorrected	ACE	BFC	BLESS2	Hybrid	Karect	BrownieCorrector	Reckoner
# contigs (≥ 0 bp)	149228	191379	166969	174905	162558	163826	149066	199481
# contigs (≥ 1000 bp)	3216	2996	3107	3393	2749	2859	3001	3290
# contigs (≥ 5000 bp)	1718	1706	1683	1903	1399	1483	1547	1855
# contigs (≥ 10000 bp)	1201	1308	1177	1441	984	1053	1068	1340
# contigs (≥ 25000 bp)	779	854	759	956	627	663	697	862
# contigs (≥ 50000 bp)	536	560	529	637	459	474	489	572
Total length (≥ 0 bp)	137006538	137208923	138629176	135737931	138182929	138240801	137010216	**140815351**
Total length (≥ 1000 bp)	122386145	119633304	**122499150**	119842489	122364837	122306783	122404017	122425002
Total length (≥ 5000 bp)	118896712	116646454	119167322	116397113	**119223919**	119086628	119025946	119068877
Total length (≥ 10000 bp)	115233784	113711648	115580814	113066925	**116307772**	116071704	115635740	115382004
Total length (≥ 25000 bp)	108540040	106412774	108957987	105232289	**110727634**	110012601	109875005	107869724
Total length (≥ 50000 bp)	99903980	95839747	100902805	93696395	**104904218**	103401295	102484228	97699745
# contigs	5319	4525	5155	4582	4748	4889	5076	5379
Largest contig	1078505	1097937	1329229	873186	**1577991**	1472989	1315292	834563
Total length	123920625	120711602	**123995028**	120697350	123832426	123797326	123920633	123949799
Reference length	120381546	120381546	120381546	120381546	120381546	120381546	120381546	120381546
GC (%)	42.44	42.48	42.44	42.45	42.46	42.46	42.44	42.44
Reference GC (%)	42.42	42.42	42.42	42.42	42.42	42.42	42.42	42.42
N50	195145	173427	209236	141236	**278523**	252831	240349	164454
NG50	202478	173448	217832	141956	**293584**	264260	250321	175352
N75	73641	66031	77292	56469	**102606**	93760	88826	64991
NG75	83516	66621	85145	56958	**113846**	104548	101313	71933
L50	170	196	156	241	**115**	126	140	196
LG50	161	195	148	240	**109**	120	133	185
L75	422	468	402	578	**294**	324	348	489
LG75	388	464	368	574	**270**	298	319	449
# misassemblies	936	999	966	**933**	952	944	934	998
# misassembled contigs	497	557	490	588	**438**	456	467	558
Misassembled contigs length	79855723	76678055	81266528	**67931475**	88449521	86859421	85282570	78755301
# local misassemblies	1646	1697	1741	1685	1646	**1619**	1658	1690
# unaligned mis. contigs	101	101	116	107	107	113	**97**	108
# unaligned contigs	3150 + 604 part	2051 + 605 part	3015 + 629 part	**1916 + 581 part**	2976 + 597 part	2979 + 602 part	3137 + 599 part	3061 + 610 part
Unaligned length	9788711	**6681807**	9833294	6696692	9705532	9659228	9818310	9743468
Genome fraction (%)	**94.396**	94.239	94.368	94.186	94.387	**94.396**	94.391	94.363
Duplication ratio	**1.004**	1.005	1.005	1.005	**1.004**	**1.004**	**1.004**	1.005
# N's per 100 kbp	883.63	**691.64**	948.10	791.18	962.03	945.40	887.88	951.17
# mismatches per 100 kbp	505.84	504.41	501.43	504.88	501.19	**500.63**	505.00	502.12
# indels per 100 kbp	120.65	119.95	118.99	122.14	**118.96**	119.05	120.48	119.18
Largest alignment	562955	488967	779743	508037	630299	**824208**	630640	562981
Total aligned length	113900519	113715614	113916864	113699300	113906488	113910162	113887441	**113924772**
NA50	106095	92928	106110	86055	**121172**	119421	113318	95924
NGA50	109785	93602	110748	86526	**126449**	124215	118192	99419
NA75	41483	39367	43182	37065	**51555**	48231	47013	37077
NGA75	45799	40003	47338	37379	**57604**	54004	52514	41418
LA50	338	362	322	388	**280**	291	307	360
LGA50	322	360	305	386	**266**	277	291	341
LA75	798	848	778	913	**663**	691	720	871
LGA75	737	842	718	907	**615**	641	667	803

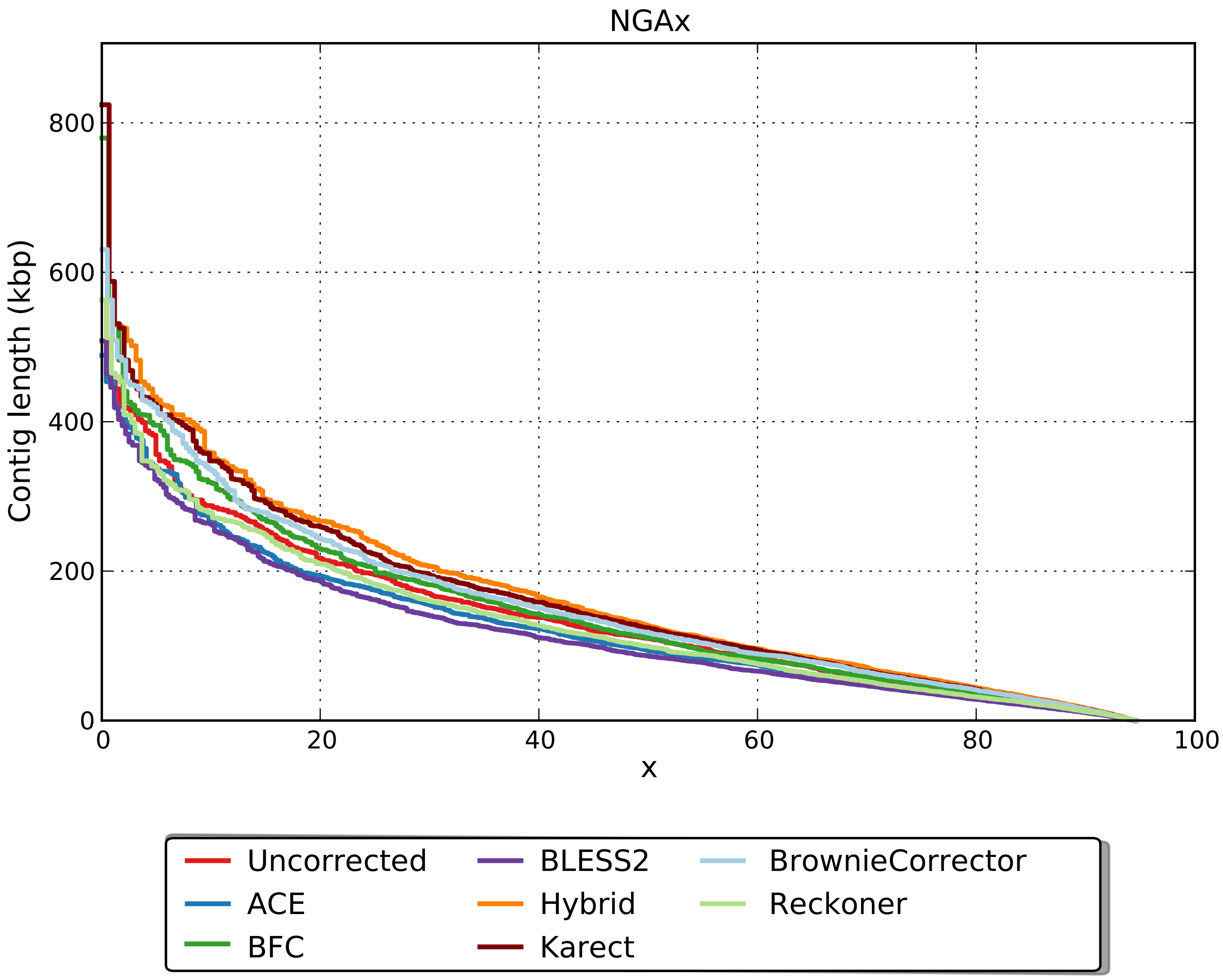

Figure 19: SPAdes assembly results for dataset D8 (*D. melanogaster*) for both uncorrected and corrected data. Contigs with length NGAx or larger produce x% of the genome.

5.5.9 D9

Table 24 contains the Quast report after assembling dataset D9 (*A. thaliana*) with SPAdes. This is a hybrid assembly in which the corrected (and uncorrected) Illumina reads (R6) are complemented with the Pacbio reads (P2). Fig. 20 shows the corresponding NGAx plot.

Table 24: Assembly quality metrics for D9

Assembly	Uncorrected	ACE	BFC	BLESS2	Hybrid	Karect	BrownieCorrector	Reckoner
# contigs (≥ 0 bp)	142089	157367	141808	146982	145087	146112	142070	157568
# contigs (≥ 1000 bp)	3227	3340	3189	3629	3097	3197	3090	3412
# contigs (≥ 5000 bp)	1915	1998	1926	2157	1777	1864	1791	2077
# contigs (≥ 10000 bp)	1460	1528	1455	1629	1365	1427	1365	1612
# contigs (≥ 25000 bp)	954	947	966	1011	857	908	877	1054
# contigs (≥ 50000 bp)	615	604	613	588	559	587	565	654
Total length (≥ 0 bp)	123813843	124547852	123778880	123452408	124109646	124097329	123825918	**124894825**
Total length (≥ 1000 bp)	110920359	110650660	110973623	110165631	**111016302**	110947707	110946362	110941841
Total length (≥ 5000 bp)	107630324	107241203	**107824566**	106411218	107715793	107603069	107704107	107587644
Total length (≥ 10000 bp)	104366913	103820948	104426193	102640491	**104764365**	104467517	104638648	104241224
Total length (≥ 25000 bp)	96121787	94226910	96285383	92636453	96331162	95891626	**96638802**	95024283
Total length (≥ 50000 bp)	83940356	82224621	83679724	77761041	**85845567**	84494501	85456925	80760253
# contigs	4250	4359	4177	4656	4095	4195	4100	4398
Largest contig	692396	548881	692397	664835	706587	**799938**	673567	568204
Total length	111616529	111339614	111643887	110865743	**111696039**	111628269	111630438	111613165
Reference length	119668634	119668634	119668634	119668634	119668634	119668634	119668634	119668634
GC (%)	35.94	35.96	35.95	35.96	35.95	35.95	35.94	35.94
Reference GC (%)	36.06	36.06	36.06	36.06	36.06	36.06	36.06	36.06
N50	122106	116453	117977	101712	**150944**	130668	143649	100710
NG50	108902	107645	104580	92073	**132808**	117943	129072	93533
N75	50880	46312	49848	39472	**56026**	52705	54148	44144
NG75	39074	33291	36446	29040	39395	37369	**40588**	33255
L50	254	265	254	279	**212**	237	218	308
LG50	289	302	289	324	**240**	270	248	350
L75	611	631	615	710	**520**	572	532	717
LG75	748	791	754	904	**648**	708	661	873
# misassemblies	837	836	814	829	793	**775**	800	830
# misassembled contigs	554	582	552	581	526	528	**523**	555
Misassembled contigs length	43095471	42734679	44104032	**36877771**	45541239	43327594	45333670	38686180
# local misassemblies	644	641	648	686	**638**	**638**	640	723
# unaligned mis. contigs	13	8	10	12	8	**7**	9	8
# unaligned contigs	266 + 425 part	191 + 409 part	245 + 402 part	**147 + 388 part**	248 + 400 part	263 + 379 part	259 + 411 part	262 + 407 part
Unaligned length	1229535	1018600	1151096	**999954**	1227225	1154999	1226425	1152483
Genome fraction (%)	91.805	91.747	**91.912**	91.363	91.876	91.866	91.824	91.849
Duplication ratio	1.005	1.005	1.005	1.005	1.005	1.005	1.005	1.005
# N's per 100 kbp	511.05	490.09	475.62	493.59	505.76	**471.17**	501.25	477.68
# mismatches per 100 kbp	52.02	59.49	52.33	59.19	52.05	52.67	51.50	**51.00**
# indels per 100 kbp	11.29	16.44	11.22	21.71	10.74	10.92	**10.73**	**10.73**
Largest alignment	559314	527818	619363	663821	635802	648971	**671915**	567176
Total aligned length	110285821	110197159	**110387175**	109731628	110371573	110368712	110301676	110342848
NA50	95741	93437	94016	85059	**114187**	101700	110521	80931
NGA50	84659	83138	82101	74447	**104037**	90661	96916	71646
NA75	38622	34585	37278	31270	40532	38578	**40986**	34410
NGA75	27591	25059	27698	22761	28609	28222	**29163**	25910
LA50	315	322	322	336	**268**	292	274	376
LGA50	360	369	367	391	**304**	334	312	429
LA75	779	805	793	884	**678**	734	694	903
LGA75	965	1019	980	1131	**853**	916	868	1104

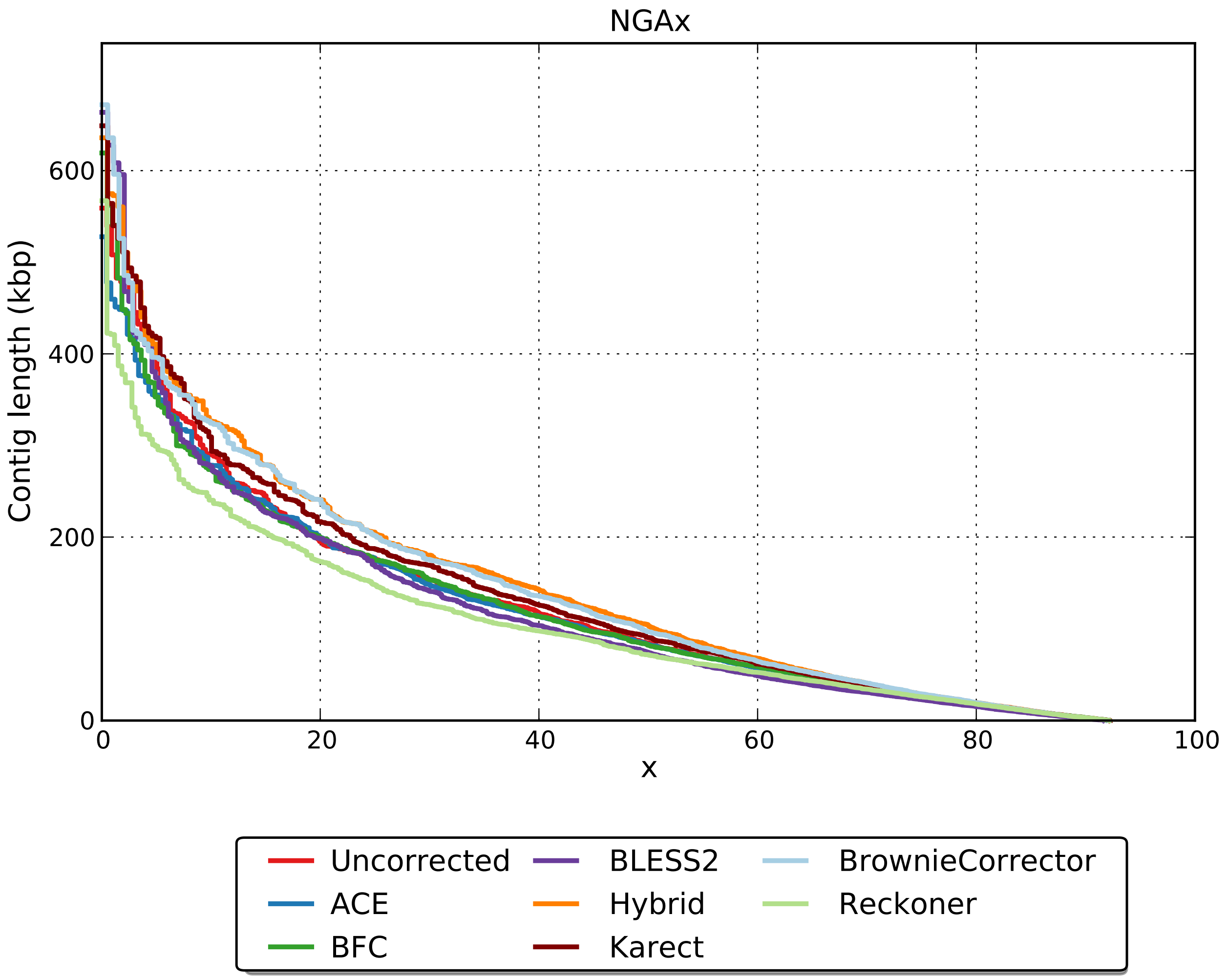

Figure 20: SPAdes assembly results for dataset D9 (*A. thaliana*) for both uncorrected and corrected data. Contigs with length NGAx or larger produce x% of the genome.

5.6 Runtime and memory usage

Table 25 and 26 provide the detail numbers of peak memory usage and the runtime (wall time) of EC tools on datasets respectively.

Table 25: Peak memory (GB) usage of the aligners on real data.

Tools	D1	D2	D3	D4	D5	D6
ACE	6.81	27.34	30.41	31.18	39.15	30.73
BFC	**2.43**	4.73	5.15	5.21	5.23	5.29
BLESS2	3.90	**3.90**	**3.90**	**3.90**	**3.90**	**3.89**
BrownieCorrector	2.64	20.66	17.19	4.70	7.11	7.21
Karect	29.76	86.99	136.64	145.25	171.37	188.59
Reckoner	3.75	3.94	3.95	3.94	3.97	3.96

Table 26: Run time (min) of the aligners on real data

Tools	D1	D2	D3	D4	D5	D6
ACE	67.03	182.62	325.02	329.82	370.43	355.25
BFC	0.91	**2.71**	3.41	4.20	5.41	6.50
BLESS2	1.25	4.49	4.83	6.31	8.85	8.51
BrownieCorrector	27.72	96.40	48.85	46.23	57.95	40.11
Karect	6.96	31.44	55.03	62.78	69.68	52.35
Reckoner	**0.70**	3.44	**3.05**	**2.97**	**4.61**	**3.45**

References

[1] Heydari, M., Miclotte, G., Van de Peer, Y., Fostier, J.: BrownieAligner: accurate alignment of illumina sequencing data to de bruijn graphs. BMC Bioinformatics **19**(1) (2018)
